# A Four-Biomarker Blood Signature Discriminates Systemic Inflammation Due to Viral Infection Versus Other Etiologies

**DOI:** 10.1038/s41598-017-02325-8

**Published:** 2017-06-06

**Authors:** D. L. Sampson, B. A. Fox, T. D. Yager, S. Bhide, S. Cermelli, L. C. McHugh, T. A. Seldon, R. A. Brandon, E. Sullivan, J. J. Zimmerman, M. Noursadeghi, R. B. Brandon

**Affiliations:** 1 Immunexpress Inc., Seattle, WA 98109 USA; 2 Division of Pediatric Critical Care Medicine, Seattle Children’s Hospital, Seattle, WA 98105 USA; 3 Department of Pediatrics, University of Washington School of Medicine, Seattle, WA 98195 USA; 4 Division of Infection & Immunity, University College London, Cruciform Building, 90 Gower Street, London, WC1E 6BT United Kingdom; 5 National Institute for Health Research Biomedical Research Centre, University College London Hospitals, 149 Tottenham Court Road, London, W1T 7DN United Kingdom

## Abstract

The innate immune system of humans and other mammals responds to pathogen-associated molecular patterns (PAMPs) that are conserved across broad classes of infectious agents such as bacteria and viruses. We hypothesized that a blood-based transcriptional signature could be discovered indicating a host systemic response to viral infection. Previous work identified host transcriptional signatures to individual viruses including influenza, respiratory syncytial virus and dengue, but the generality of these signatures across all viral infection types has not been established. Based on 44 publicly available datasets and two clinical studies of our own design, we discovered and validated a four-gene expression signature in whole blood, indicative of a general host systemic response to many types of viral infection. The signature’s genes are: Interferon Stimulated Gene 15 (*ISG15*), Interleukin 16 (*IL16*), 2′,5′-Oligoadenylate Synthetase Like (*OASL*), and Adhesion G Protein Coupled Receptor E5 (*ADGRE5*). In each of 13 validation datasets encompassing human, macaque, chimpanzee, pig, mouse, rat and all seven Baltimore virus classification groups, the signature provides statistically significant (p < 0.05) discrimination between viral and non-viral conditions. The signature may have clinical utility for differentiating host systemic inflammation (SI) due to viral versus bacterial or non-infectious causes.

## Introduction

Systemic inflammation (SI), as indicated by clinical signs such as fever and increased respiratory and heart rates, can be due to a variety of underlying non-infectious or infectious causes including trauma, thermal burns, surgery, ischemia-reperfusion events and viral or bacterial infections. Patients presenting with SI can pose a diagnostic challenge for clinicians in determining the underlying etiology; consequently it can be difficult to select the most appropriate options for treatment and patient management^1–5^. There is a clinical need for rapid diagnostic tests that can help clinicians distinguish between non-infectious, viral and bacterial etiologies of SI in (critically ill) patients. Without such tests, patients may be over-prescribed antibiotics when there is little clinical evidence of infection^4, 6^. Reducing inappropriate and unnecessary use of antibiotics, the concept of antibiotic stewardship, is essential in slowing the spread of resistant bacteria^7^.

Traditional reference methods for determining bacterial or viral causes of SI involve the culture, isolation and identification of causative pathogens from multiple specimens from a patient. Such an approach, however, has several limitations: (i) the causative pathogen might not be present in the specimens taken for examination; (ii) the specimens may become contaminated by organisms unrelated to the cause of infection; (iii) multiple organisms may be present in the specimens (*e.g*. due to contamination or non-harmful microbiota) and it can be difficult to determine which organism is the cause of the presenting clinical signs^8–10^. Furthermore, (iv) some sampling techniques (e.g. bronchoalveolar lavage, lumbar puncture) are relatively invasive. Finally, (v) some pathogens are not easily cultured. Although traditional culture-based methods are steadily being supplemented or displaced by immunological and molecular methods such as rapid immunoassays and polymerase chain reaction (PCR)^11, 12^, these newer methods also suffer from limitations, for example: (i) an inability to detect organisms not represented in an immunoassay or PCR panel; (ii) an inability to discriminate between live and dead organisms in a specimen; and (iii) a tendency to detect low levels of virus that may not be clinically relevant^13^.

Given these limitations, increasing attention is being paid to an alternative approach: that of identifying biomarkers that reflect the differential host response to underlying non-infectious, bacterial, or viral conditions^14–23^. Our current investigation builds upon and extends previous host biomarker studies by identifying a molecular signature that is demonstrably specific to SI caused by a broad range of pathogenic viruses that represent all seven Baltimore virus classification groups and that cause infection in different tissues in multiple mammalian species. We used, as a discriminating function, the Area Under Curve (AUC) in Receiver Operating Characteristic Curve (ROC) analysis, and boosted specificity by employing a filtering step in our discovery process whereby biomarkers with high AUCs for non-viral causes of SI were removed. Independent validation of the signature in adult and pediatric cohorts demonstrated a strong discrimination of viral vs. non-viral causes of SI. Notably, this viral signature relies on only four biomarkers, and this high degree of parsimony should help to ensure the performance robustness necessary for effective translation to a rapid point-of-care format.

## Results

### Discovery of the pan-viral signature

An initial search was conducted across 13 Gene Expression Omnibus (GEO) datasets (Table 1) from human adult and pediatric subjects, and one GEO dataset from macaques. These 14 discovery datasets (comprising 417 cases and 182 controls) spanned three Baltimore Group I viruses (cytomegalovirus, human herpesvirus 6, enterovirus), one Group III virus (rotavirus), two Group IV viruses (Dengue, hepatitis C), and six Group V viruses (influenza, Lassa virus, rhinovirus, lymphocytic choriomeningitis virus, respiratory syncytial virus, and measles virus). Next, a comprehensive, stepwise filtering approach was applied to 19 additional GEO datasets comprising a total of 1337 cases and 1106 controls (Table 1), to exclude genes that were differentially expressed in conditions that may present as SI but appear unrelated to viral systemic inflammation. The end result, after the filtering step was applied, was a “pan-viral” signature based on the expression levels of four genes: Interferon Stimulated Gene 15 (*ISG15*), Interleukin 16 (IL16), 2′,5′-Oligoadenylate Synthetase Like (*OASL*), and Adhesion G Protein Coupled Receptor E5 (*ADGRE5*). Table 2 summarizes what is known about the role, function and tissue expression of these four genes. Three of the genes (*ISG15, OASL, IL16*) have previously been reported to be associated with host response to viral infection, although they are not entirely specific to such a response. The four genes are all strongly expressed in whole blood and white blood cells, and to a lesser degree in most other tissues.

**Table 1:** Discovery datasets used in this study.

Study (Reference)	Species	Virus/Baltimore Group	Cohorts/(Samples)	Gender (M/F)	Use	AUC
GSE40366 (Reference #^88^)	Human	CMV/I	Titer 0 (17) vs. Titer >20,000 (69)	0: 5/12 > 20,000: 12/57	Discovery (Core)	0.84
GSE51808 (Reference #^89^)	Human	Dengue/IV	Healthy (9) vs. Acute (28)	Healthy: 2/7 Acute: 20/8	Discovery (Core)	1.00
GSE52428 (Reference #^18^)	Human	Influenza/V	Pre-challenge (37) vs. Symptomatics, early post-challenge (57)	Pre: 11/9 Post: 11/9	Discovery (Core)	0.93
GSE41752 (Reference #^90^)	Macaque	Lassa/V	Pre-challenge (11) vs. Post-challenge (9), Days 2, 3, 6	Male and female	Discovery (Core)	1.00
GSE6269 (Reference #^70^)	Human (pediatric)	Influenza/V	Healthy (6) vs. Influenza (18)	Healthy: 5/1 Influenza: 10/8	Discovery (Sensitivity)	0.98
GSE40396 (Reference #^17^)	Human	HHV6/I Enterovirus/I Rhinovirus/V	Control (18) vs. Infected (35)	Control: 13/5 Infected: 19/16	Discovery (Sensitivity)	0.82
GSE40012 (Reference #^16^)	Human	Influenza/V	Healthy (18) vs. Influenza (8)	Healthy: 6/12 Influenza: 3/5	Discovery (Sensitivity)	1.00
GSE18090 (Reference #^91^)	Human	Dengue/IV	Uninfected febrile (8) vs. Infected febrile (17)	Uninfected: 4/4 Infected: 7/10	Discovery (Sensitivity)	0.79
GSE30550 (Reference #^92^)	Human	Influenza/V	Pre-challenge (8) vs. Post-challenge (61) Note: only the symptomatic patients were analyzed. For the post-challenge timepoints, only the timepoints between 21–69 hours were considered.	Information not available	Discovery (Sensitivity)	0.95
GSE40224 (Reference #^93^)	Human	Hepatitis C/IV	Healthy (6) vs. Infected (10)	Healthy: 5/1 Infected: 10/0	Discovery (Sensitivity)	0.86
GSE5790 (Reference #^94^)	Macaque	LCMV/V	Pre-challenge (11) vs. Post-challenge (11) Time course	Not stated	Discovery (Sensitivity)	0.64
GSE34205 (Reference #^95^)	Human (pediatric)	Influenza/V RSV/V	Healthy (22) vs. Influenza (28) RSV (51)	Healthy: 14/8 Influenza: 13/15 RSV: 24/27	Discovery (Sensitivity)	0.83
GSE5808 (Reference #^96^)	Human (pediatric)	Measles/V	Healthy (3) vs. Infected (5)	Healthy: 2/1 Infected: 3/2	Discovery (Sensitivity)	0.87
GSE2729 (Reference #^97^)	Human (pediatric)	Rotavirus/III	Healthy (8) vs. Infected (10)	Healthy: not stated Infected: 3/5	Discovery (Sensitivity)	0.99
GSE33341 (Reference #^98^)	Human	N/A	Healthy (43) vs. Bacteremia (49)	Healthy: 23/20 Bacteremia: 28/21	Discovery (Specificity)	0.79
GSE40366 (Reference #^88^)	Human	N/A	Nonagenarian (6) vs. Young (11)	Nonagenarian: 2/4 Young: 3/8	Discovery (Specificity)	0.71
GSE42834 (Reference #^99^)	Human	N/A	Healthy (113) vs. TB (35) Active sarcoidosis (39) Lung cancer (16) Bacterial pneumonia (14)	Healthy: (41/72) TB: (19/16) Sarcoidosis: (15/22, with 2 not stated) Lung cancer: (10/6) Pneumonia: (9/5)	Discovery (Specificity)	0.76
GSE25504 (Reference #^100^)	Human (neonate)	N/A	Healthy (26) vs. Sepsis (25)	Healthy: 17/9 Sepsis: 13/12	Discovery (Specificity)	0.63
GSE30119 (Reference #^101^)	Human (pediatric)	N/A	Healthy (44) vs. Sepsis (99)	Healthy: 23/21 Sepsis: 54/45	Discovery (Specificity)	0.52
GSE17755 (Reference #^102^)	Human	N/A	Healthy (53) vs. Autoimmunity (191)	Healthy: 29/24 Autoimmunity: 39/152	Discovery (Specificity)	0.76
GSE19301 (Reference #^103^)	Human	N/A	Asthma quiet (292) vs. Asthma exacerbation (117)	64.4% female	Discovery (Specificity)	0.63
GSE38485 (Reference #^104^)	Human	N/A	Healthy (22) vs. Schizophrenia (15)	Healthy: 16/6 Schizophrenia: 11/4	Discovery (Specificity)	0.676
GSE36809 (Reference #^105^)	Human	N/A	Healthy (37) vs. Blunt trauma (first 12 hours (150))	Healthy: 20/17 Trauma: 94/56	Discovery (Specificity)	0.53
GSE46743 Unpublished	Human	N/A	Dexamethazone Pre-dose (160) vs. Post-dose (160)	All males	Discovery (Specificity)	0.58
GSE61672 (Reference #^106^)	Human	N/A	Anxiety Controls (179) vs. Patients (157)	Controls: 70/109 Patients: 43/114	Discovery (Specificity)	0.57
GSE16129 (Reference #^107^)	Human	N/A	Healthy (10) vs. S aureus sepsis (46)	Healthy: 5/5 Sepsis: 29/17	Discovery (Specificity)	0.54
GSE40012 (Reference #^16^)	Human	N/A	Healthy (18) vs. SIRS (12)	Healthy: 6/12 SIRS: 10/2	Discovery (Specificity)	0.57
GSE40396 (Reference #^17^)	Human (pediatric)	N/A	Controls (22) vs. Bacteremia (8)	Controls: 15/7 Bacteremia: 6/2	Discovery (Specificity)	0.49
GSE6269 (Reference #^70^)	Human (pediatric)	N/A	Healthy (6) vs. Bacterial infection (73)	Healthy: 1/5 Sepsis: 41/32	Discovery (Specificity)	0.61
GSE35846 (Reference #^108^)	Human	N/A	Gender: Men (65) vs. Women (124)	65/124	Discovery (Specificity)	0.57
GSE35846 (Reference #^108^)	Human	N/A	Race: Caucasian (140) vs. African American (37)	Caucasian: 54/86 African-American: 3/34	Discovery (Specificity)	0.50
GSE35846 (Reference #^108^)	Human	N/A	Body fat: 9–30% (80) vs. 31–53% (109)	53/27 vs. 12/97	Discovery (Specificity)	0.47
GSE35846 (Reference #^108^)	Human	N/A	Age: 26–60 (161) vs. 61–79 (28)	54/107 vs. 11/17	Discovery (Specificity)	0.50

Details of datasets used for discovery of the pan-viral signature are listed including: an associated reference (if available), species studied, virus types represented, cohorts compared and the number of samples in each, gender numbers, how the dataset was used, and the performance (AUC) of the pan-viral signature in the cohorts described. Sample type analyzed was blood for all datasets in this table.

**Table 2:** RNA transcripts comprising the pan-viral signature.

Gene	Role/Function/Tissue Expression	References
*ISG15* Interferon-stimulated gene 15; Ubiquitin-like modifier	Key role in innate immune response to viruses including influenza, HIV-1 and Ebola. Induces gamma interferon and natural killer cell proliferation. Chemotactic for neutrophils. Strongly expressed in EBV-transformed lymphocytes.	HGNC Symbol: *ISG15* OMIM: 14751, #616126 References #^109^–^112^
*IL16* Interleukin-16	Pleiotropic cytokine that functions as a chemoattractant, a modulator of T cell activation, and an inhibitor of HIV replication. Ligand for CD4. Strongly expressed in whole blood, brain, spleen and EBV-transformed lymphocytes. Upregulated in viral infections.	HGNC Symbol: *IL16* OMIM: 603035 References #^59^, ^113^, ^114^
*OASL* 2′–5′-Oligoadenylate Synthetase-Like	Displays antiviral activity against encephalomyocarditis virus (EMCV) and hepatitis C virus (HCV) via an alternative antiviral pathway independent of RNase L. Can bind double stranded RNA. Strongly expressed in whole blood and EBV-transformed lymphocytes.	HGNC Symbol: *OASL* OMIM: 603281 References #^52^, ^115^, ^116^
*ADGRE5* (*CD97*) Adhesion G Protein-Coupled Receptor E5	May play a role in cell adhesion as well as leukocyte recruitment, activation and migration. Contains multiple extracellular EGF-like repeats which mediate binding to chondroitin sulfate and the cell surface complement regulatory protein CD55. Strongly expressed in whole blood, spleen and arterial tissue.	HGNC Symbol: *ADGRE5* OMIM: 601211 References #^117^, ^118^

Details of the role, function and tissue expression of the underlying genes are provided along with associated references. *ISG15*, *IL16* and *OASL* have previously been directly linked to an immune response to a virus infection.

### Validation of the pan-viral signature in independent GEO datasets

To ensure the resulting pan-viral signature was not overfit to the discovery datasets and was generalizable across different viruses and mammalian species, we next validated its performance in 13 human and non-human mammalian datasets (11 from GEO and 2 from clinical trials, comprising a total of 332 cases and 302 controls). Importantly, these datasets represented a completely independent set of observations to those used during the discovery process. The validation datasets were chosen on the basis of (i) coverage of all seven of the Baltimore virus classification groups, and (ii) the potential impact of each virus on human health. In the case of the human datasets, the subjects had either naturally acquired viral infections, or had been vaccinated with attenuated viral vaccines (see Table 3 for details). The AUCs for performance of the pan-viral signature in the validation datasets ranged from 0.90 to 0.98.

**Table 3:** Validation datasets used in this study.

Study (Reference)	Species	Virus/Baltimore Group	Cohorts/(Samples)	Gender (M/F)	Use	AUC or p-value
GSE4128 (Reference #^28^)	Mouse^1^ (liver)	Adenovirus/I	Mock (6 knockout, 5 wild-type) vs. Vector-injected (11 knockout, 5 wild-type)	Not stated	Validation	1.00
GSE14790 (Reference #^30^)	Pig	Porcine circovirus, type 2	Uninfected (4) vs. Infected (4)	Not stated	Validation	0.94 (day 7 vs. day 0); 1.00 (days 14, 21, 29 vs. day 0)
E-GEOD-50628 (Reference #^32^)	Human	Rotavirus/III	Acute (6) vs. Recovery (6)	4/2	Validation	p < 0.05^2^
GSE13699 (Reference #^35^)	Human	Yellow fever (attenuated)/IV	Pre-vaccination (26) vs. Post-vaccination Days 3/7 (51) Time course	14/12	Validation	0.98
GSE69606 (Reference #^37^)	Human (pediatric)	RSV/V	Mild (9), Moderate (9), Severe (8) vs. Follow-up (17)	Mild: 6/3 Moderate: 8/1 Severe: 6/2 Follow-up: 14/3	Validation	0.90
GSE29429_GPL6947 GSE29429_GPL10558 (Reference #^38^)	Human	HIV/VI	Healthy (55) vs. Infected (58) at study entry	Healthy:31/24 Infected: 41/17	Validation	0.91
GSE68112 (Reference #^39^)	Rat (primary hepatocytes)	Adenovirus/I Hepatitis B/VII	Uninfected (6) vs. Infected with adenovirus construct (3) vs. infected with adenovirus/hepatitis B construct (3) at 48 and 72 hours; Time course	Tissue culture	Validation	p > 0.05 at 48 hours; p < 0.02 at 72 hours^3^
GSE67059 (Reference #^21^)	Human (pediatric)	HRV/IV	HRV- (37) vs. HRV + (114)	HRV−: 21/16 HRV+: 76/38	Validation	0.90
GSE57384 (Reference #^119^)	Mouse^1^	Influenza/V	Pre-challenge (30) vs. Post-challenge (30) Time course	Not stated	Validation	1.00^4^
GSE22160 (Reference #^14^)	Chimpanzee (liver biopsy)	Hepatitis E/IV Hepatitis C/IV	Pre-HEV (3) vs. Post-HEV (3) Time course; Pre-HCV (4) vs. Post-HCV (4) Time course	Not stated	Validation	1.00; 1.00
GSE58287 (References #^120^, ^121^)	Macaque	Marburg/V	Pre-challenge (15) vs. Post-challenge (15) Time course	All female	Validation	0.98
FEVER (This paper)	Human (adults)	Varicella/I Epstein-Barr/I CMV/I Influenza/V Dengue/IV	Bacterial infection (55) vs. Viral infection (15); Viral infection (15) vs. Uninfected (22); Bacterial infection (55) vs. Uninfected (22)	44/48	Validation	0.93; 0.85; 0.58
GAPPSS (This paper)	Human (pediatric)	Rhinovirus/IV Enterovirus/I Coronavirus/IV Parainfluenza/V RSV/V	Sepsis (25) vs. Viral SI (5); Sepsis + Viral SI (10) vs. Viral SI (5); Sepsis + Viral SI (10) vs. Sterile SIRS (29); Sterile SIRS (29) vs. Sepsis ± Viral SI (35); Sterile SIRS (29) vs. Viral SI (5); Sepsis vs. sterile SIRS	25/26	Validation	0.76; 0.70; 0.64; 0.62; 0.91; 0.60

Details of datasets used for validation of the pan-viral signature are listed including: an associated reference (if available), species studied, virus types represented, cohorts compared and the number of samples in each, gender numbers, how the dataset was used, and the performance (AUC) of the pan-viral signature in the cohorts described. Sample type analyzed was blood unless otherwise noted in the Species column. ^1^The mouse ortholog OASL1 of the human gene OASL was used in the analysis of mouse GEO datasets. ^2^Mann-Whitney U test. ^3^One-tailed t-test, assuming unequal variances between the two comparison groups. ^4^Pan-viral signature AUC evaluated over days 2–6 post-infection, compared to pre-infection state (GSE57384).

#### GEO Validation Dataset #1: Adenovirus (Baltimore Group I, double-stranded DNA)

**Figure 1:**
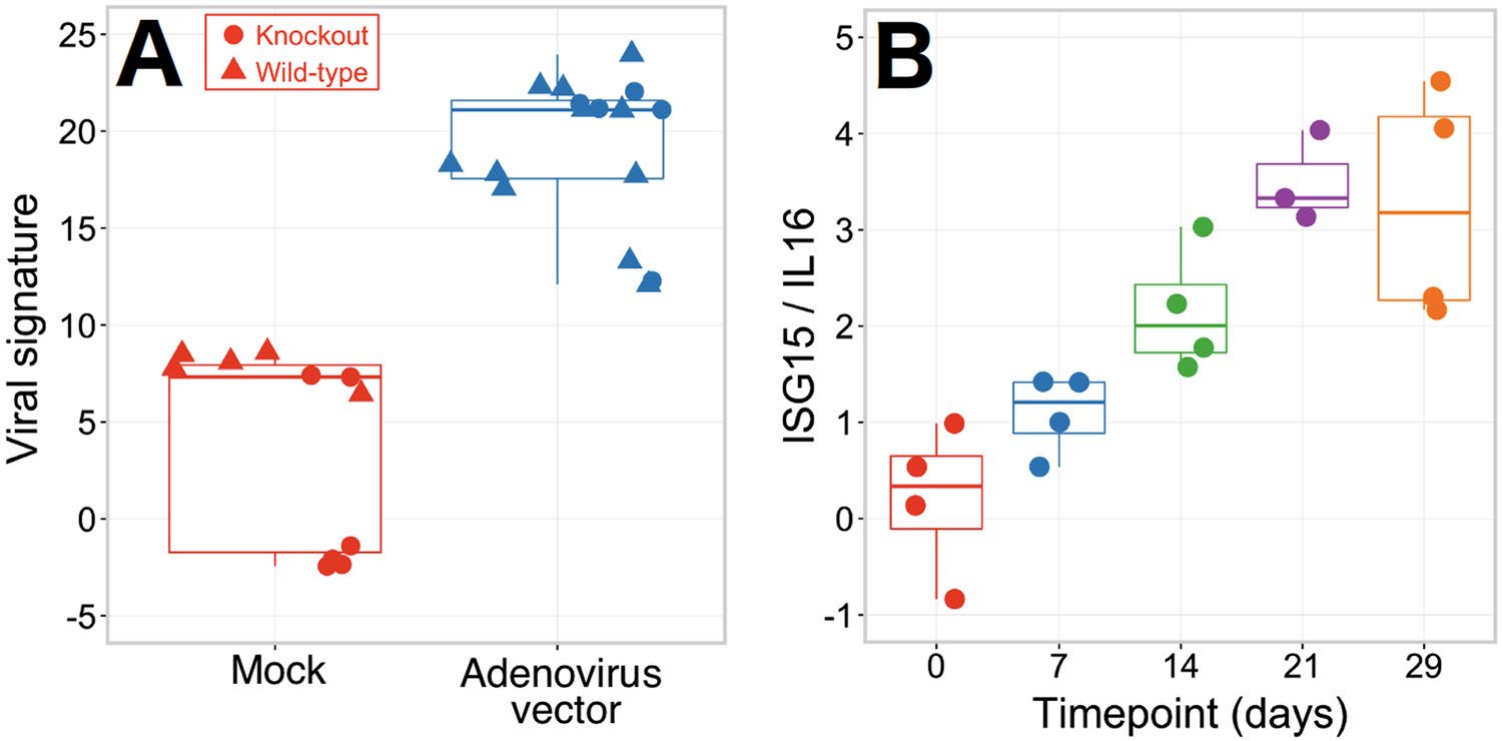
Pan-viral signature in models of infection involving DNA viruses. Panel (A): Adenovirus (double-stranded DNA; Baltimore group I). Pan-viral signature measured in liver tissue derived from wild-type or C3-knockout mice injected with phosphate buffered saline (mock) or adenovirus type 5 capsids (vector), from dataset GSE4128. The box and whisker plots show the median and interquartile range for each group. The pan-viral signature produced AUC = 1.00. Panel (B): Porcine circovirus (single-stranded DNA; Baltimore group II). Dataset GSE14790 was generated from samples of peripheral blood, drawn weekly from four Landrace CDCD piglets infected with subclinical doses of PCV2 (Burgos isolate) at day 7 post-gestational age and followed for 29 days. The OASL gene was not available on the microarray, so only the ISG15/IL16 component of the pan-viral signature is shown here, in a box-and-whisker plot of median and interquartile range for four individual piglets at different days post-inoculation. The ISG15/IL16 component of the pan-viral signature produced AUC = 0.94 for day 7 vs. day 0 comparison, and AUC = 1.00 for days 7, 14, 21, 29 vs. day 0 comparison.

Fifty-one different serotypes of adenovirus are known to infect humans, and serotypes 1, 2, 3, 4, 5, 7, 21 in particular are significant causes of upper respiratory tract infections, especially in children^24–27^. For evaluation of the performance of the pan-viral signature in Baltimore Group I viral infections, we chose GEO dataset GSE4128 which was derived from a study^28^ of mice injected with adenovirus type 5 capsids (“vector”) or phosphate buffered saline (“mock”) (Fig. 1A). Adenovirus capsids are known to induce an innate inflammatory response^28^. Gene expression analyses were performed on liver samples taken six hours post-infection for both wild type mice, and mice rendered deficient for complement component 3 (C3) by gene targeting. We observed a clear difference (AUC = 1.00) in pan-viral signature values between infected and mock-infected mice. Whilst the authors found a “blunted” immune response to adenovirus injection in the C3-deficient mice, we found little overall difference in pan-viral signature response, suggesting that the absence of C3 does not affect the pan-viral signature value. Note that for analysis of dataset GSE4128, our pan-viral signature incorporated the mouse gene 2′–5′ Oligoadenylate Synthetase-Like 1 (OASL1), which is the ortholog of human OASL^29^. Also, two samples were omitted from our analysis because the study authors labeled each sample as both ‘mock’ and ‘virus-infected’ in the phenotypic table associated with GSE4128.

#### GEO Validation Dataset #2: Porcine Circovirus PCV2 (Baltimore Group II, single-stranded DNA)

There are few publicly available datasets, in either humans or other species, that describe host gene expression in response to infection by pathogenic Baltimore Group II viruses. Some example viruses in this group include parvoviruses (B19, canine parvovirus, bocavirus, adeno-associated virus) and circoviruses (porcine circovirus, chicken anemia virus). Porcine circovirus, type 2 (PCV2) is the primary cause of post-weaning multi-systemic wasting syndrome in pigs, which has had a large economic impact in the food production industry^30^. We analyzed a time-course dataset (GSE14790) derived from peripheral blood samples from Landrace cesarian-derived colostrum-deprived (CDCD) piglets infected, at post-gestation day 7, with subclinical doses of porcine circovirus 2 (PCV2, Burgos isolate). This study^30^ used an Affymetrix 24 K Genechip Porcine Genome Array to generate gene expression data. This microarray unfortunately did not include the OASL gene. We therefore were limited to analyzing this dataset using a linear combination of just two of the four genes, ISG15 and IL16, which carries most of the diagnostic power of the signature. Figure 1B shows box and whisker plots for ISG15/IL16 performance on weekly whole blood samples out to 29 days post-inoculation in piglets infected with PCV2. The ISG15/IL16 component of the pan-viral signature produced AUC = 0.94 for day 7 vs. day 0 comparison, and AUC = 1.00 for days 14, 21, 29 vs. day 0 comparison.

#### GEO Validation Dataset #3: Rotavirus (Baltimore Group III, double-stranded RNA)

**Figure 2:**
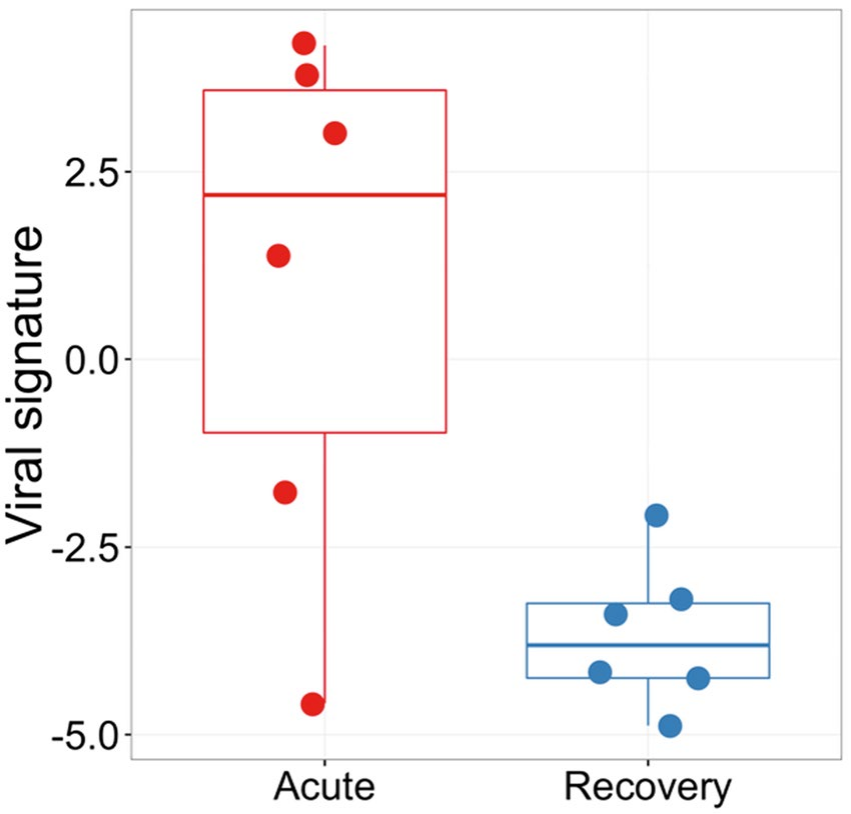
Pan-viral signature scores for children with rotavirus infection. Box and whisker plots showing pan-viral signature scores in peripheral blood for six children in the acute versus recovery stages of rotavirus infection (E-GEOD-50628). The pan-viral signature produced AUC = 0.86.

Rotaviruses are the most common cause of gastroenteritis worldwide in children less than five years of age, resulting in over 2 million hospitalizations annually^31^. Despite the main clinical signs of rotavirus infection being related to gastroenteritis, peripheral blood gene expression changes associated with infection have been reported^32^. We analyzed dataset E-GEOD-50628, generated from peripheral blood samples from six children with rotavirus infections in the acute phase (2–4 days from disease onset) versus recovery phase (7–11 days from disease onset)^32^. Figure 2 shows a box and whisker plot demonstrating that the pan-viral signature can be used to differentiate between children acutely infected with rotavirus from those in recovery (p < 0.05 by Mann-Whitney-U test).

#### GEO Validation Dataset #4: Yellow Fever Virus (Baltimore Group IV, positive-sense single-stranded RNA)

**Figure 3:**
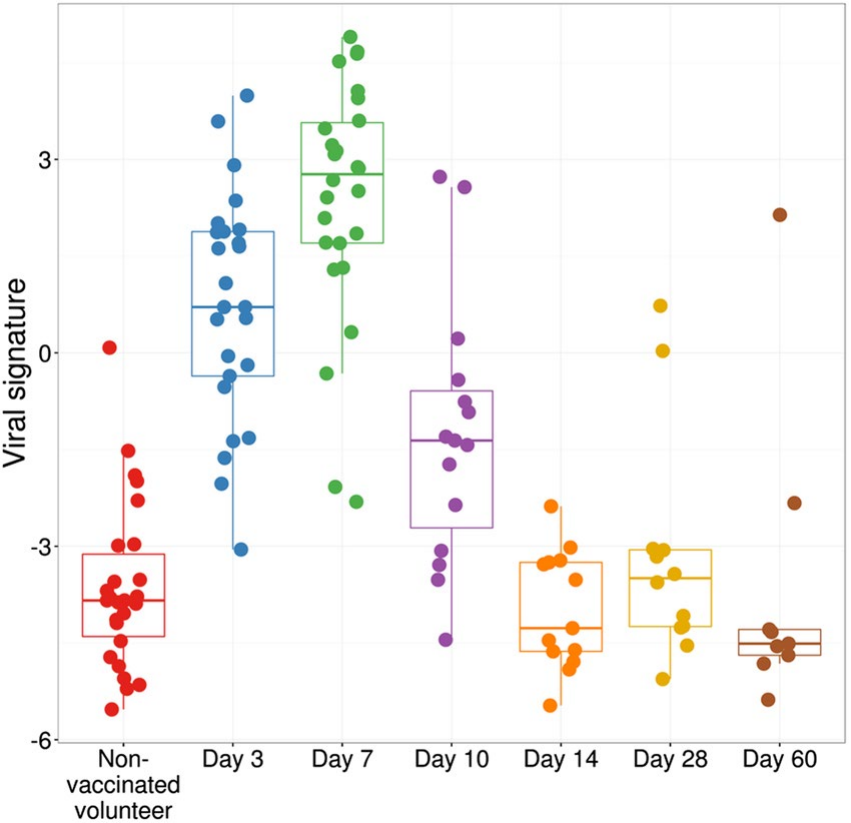
Time-course of pan-viral signature score for human volunteers vaccinated with live attenuated yellow fever vaccine. Box and whisker plot of the pan-viral signature values for 26 human volunteers vaccinated with Stamaril and followed for up to 60 days (GEO dataset GSE13699). The pan-viral value increased from day 3 post-vaccination and peaked on day 7. By day 14 the value had returned to pre-vaccination levels.

The flaviviridae family includes yellow fever, dengue, hepatitis C, Japanese encephalitis and Zika virus which together impact the lives of millions of people^33^. Yellow fever virus is considered to be a prototypical flavivirus, for which single-dose vaccination with a live attenuated virus is an effective protection^34^. We analyzed GEO dataset GSE13699 from a yellow fever vaccination study^35^ in which two geographically separated groups of volunteers (Lausanne, n = 11; Montreal, n = 15) were vaccinated subcutaneously on day 0 with Stamaril vaccine (Sanofi-Pasteur YF17D-204 YF-VAX), a vaccine containing live attenuated yellow fever virus that confers protection from 10 days following vaccination. Whole blood samples were collected on days 0, 3 and 7 for the Lausanne cohort and on days 0, 3, 7, 10, 14, 28 and 60 for the Montreal cohort. The pan-viral signature value peaked on day 7 following vaccination and dropped to pre-vaccination levels by day 14 (Fig. 3). The temporal behavior of the pan-viral signature suggests that the vaccine engenders an immune response that peaks on day 7 but does not persist beyond day 14 (as might be expected for the response to an attenuated vaccine).

#### GEO Validation Dataset #5: Respiratory Syncytial Virus (Baltimore Group V; negative-sense single-stranded RNA)

**Figure 4:**
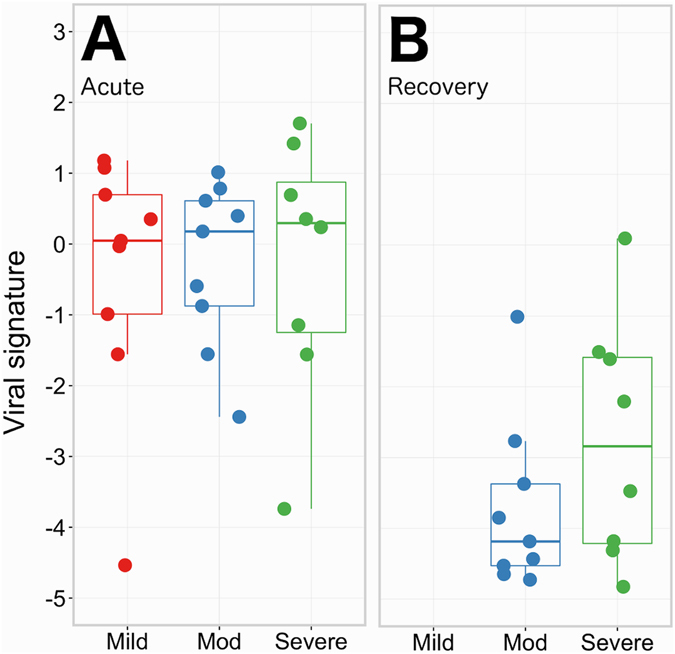
Pan-viral signature score for children with acute RSV infection and following recovery. Box and whisker plots for dataset GSE69606. Panel (A): pan-viral signature for children with acute RSV infection. Panel (B): pan-viral signature for moderately and severely affected children in recovery (4–6 weeks later). AUC = 0.903 for the difference between acute and recovery datasets. Abbreviation: Mod, moderate.

The most common cause of acute lower respiratory infection in children less than five years of age is respiratory syncytial virus (RSV), with an estimated 3.4 million infected children requiring hospitalization each year worldwide^36^. We analyzed GEO dataset GSE69606, which was generated in a study designed to identify biomarkers of RSV infection severity in children^37^. In this study, peripheral blood samples were collected from children with mild (n = 9), moderate (n = 9) or severe (n = 8) clinical signs during the acute stage of infection. An additional set of samples was collected 4–6 weeks later from recovered children who originally displayed moderate or severe clinical signs. The pan-viral signature score showed a clear difference between acute and recovery stages (AUC = 0.903), but was invariant in the acute stage regardless of RSV infection severity (Fig. 4).

#### GEO Validation Dataset #6: HIV-1 Virus (Baltimore Group VI, positive-sense single-stranded RNA virus, replicating through a DNA intermediate)

**Figure 5:**
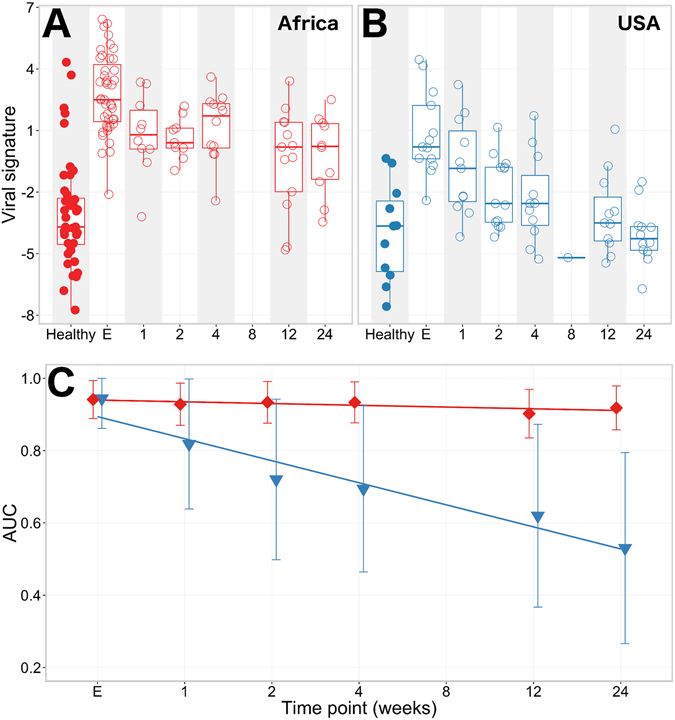
Pan-viral signature AUCs for patients with acute HIV-1 infection compared to matched uninfected healthy subjects (GEO dataset GSE29429). Panel (A): Pan-viral signature score for healthy African controls (solid points) versus HIV-1 -positive untreated African subjects (open points). Panel (B): Pan-viral signature score for healthy American controls (solid points) versus HIV-1 -positive ART-treated American subjects (open points). Panel (C): When untreated African patients were compared to the corresponding healthy African controls, the pan-viral signature AUC (±95% CI) remained at or above 0.9 for all timepoints (red diamonds). In contrast, when American patients receiving ART-treatment were compared to the corresponding American controls, the pan-viral signature AUC dropped from above 0.9 at enrolment to less than 0.5 by week 24 (blue triangles). Abbreviations: ROC, receiver operating characteristic curve; ART, anti-retroviral therapy; AUC, area under curve.

The initial clinical signs of acute HIV-1 infection are relatively non-specific, involving fever and influenza-like illness, which bear a clinical resemblance to other types of infection including bacterial sepsis. We analyzed GEO dataset GSE29429 which was generated from a time-course study^38^ comparing (A) HIV-1 infected adults who first presented in the acute stage of infection but who did not receive antiretroviral therapy (ART; African, n = 43), versus (B) HIV-1 infected adults who presented similarly but did receive ART (USA, n = 15). The study also included two sets of matched healthy controls (n = 55). Blood samples were collected at study enrollment when the patients had a confirmed acute infection, and at post-enrollment weeks 1, 2, 4, 8, 12 and 24. Figure 5 shows AUCs over time for the pan-viral signature when comparing the healthy controls to either the untreated African patients (panel A) or the treated American patients (panel B). The pan-viral signature AUC when comparing the untreated African patients to the corresponding healthy African controls remained at or above 0.9 at all time points; in contrast, the AUC when comparing the treated American patients to the corresponding healthy American controls dropped from above 0.9 at enrolment to less than 0.5 by Week 24 (panel C). The decrease in pan-viral signature values in the treated American patients also reflected a corresponding decrease in mean HIV-1 viral loads from ~800,000 virus particles/mL blood at study entry to ~2,000 virus particles/mL blood by week 24.

#### GEO Validation Dataset #7: Hepatitis B (Baltimore Group VII, double-stranded DNA virus, replicating through a single-stranded RNA intermediate)

**Figure 6:**
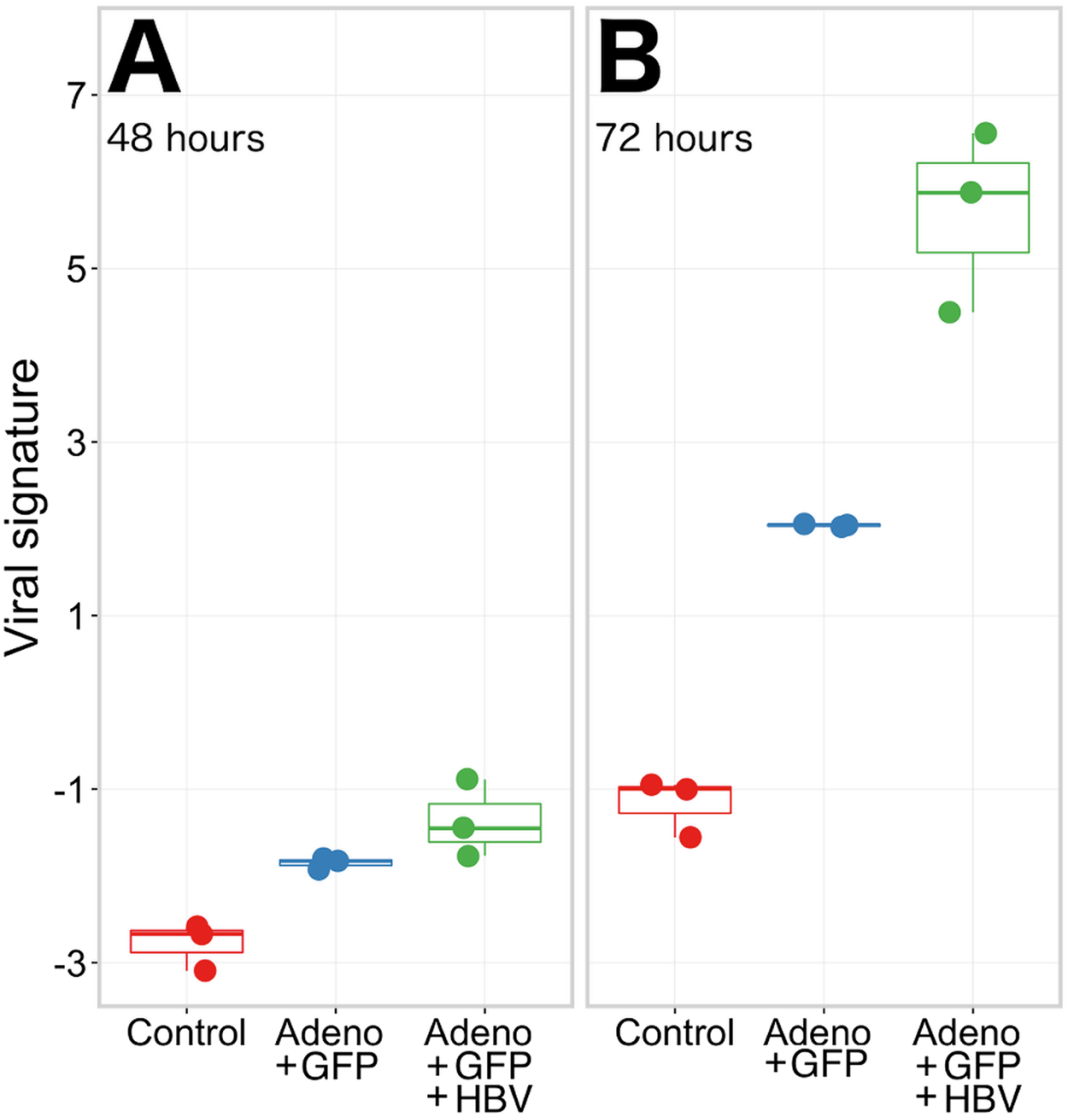
Pan-viral signature scores in primary rat hepatocytes infected with an adenovirus vector containing GFP, or GFP plus Hepatitis B virus. Box-and-whisker plots for the pan-viral signature score over time course of infection in primary rat hepatocytes (GEO dataset GSE68112). Primary rat hepatocytes were plated at 0 hours, and then infected with an adenovirus vector (Control), the adenovirus vector fused to a gene for Green Fluorescent Protein (Adeno+GFP), or the adenovirus vector fused to both the Green Fluorescent Protein gene and a copy of the Hepatitis B Virus genome (Adeno+GFP+HBV). Panel (A): response after 48 hours. Panel (B): response after 72 hours.

We analyzed GEO dataset GSE68112 which was generated from a study of HBV infection of primary rat hepatocytes^39^. Figure 6 shows pan-viral signature scores over a 72-hour period in primary rat hepatocytes. In this study, primary rat hepatocytes were plated at 0 hours, then infected with an adenovirus-based construct containing either the gene for Green Fluorescent Protein (GFP) alone, or a copy of the Hepatitis B Virus (HBV) genome in combination with the GFP gene. Post-infection, an increase in the pan-viral signature score was observed in rat hepatocytes infected with the adenovirus + GFP + HBV construct, compared to infection with the adenovirus + GFP construct lacking HBV. At the 48 hour timepoint, this increase was small and not statistically significant (p > 0.05 by one-tailed t-test, unequal variances assumed). However, at 72 hours post-infection, the increase was much larger and statistically significant (p < 0.02 by one-tailed t-test, unequal variances assumed). The results at 72 hours post-infection indicate that the pan-viral signature responds to acute infection by HBV in rats, in tissues other than blood, in an *in vitro* study.

In Figs 1–6 we have presented validation data representing all seven Baltimore viral classification groups. In Supplementary Figures S1–S5 we discuss additional GEO datasets, derived from human and animal peripheral blood samples, which were used to further validate the pan-viral signature. Human studies included rhinovirus (HRV) infection in children (Figure S1; Baltimore group IV; AUC 0.81-0.90); and a time-course study in which adult volunteers were inoculated with influenza virus (Figure S2; Baltimore Group IV; AUC up to 1.00). Animal studies included a time-course study of influenza in mice (Figure S3; Baltimore group IV; AUC up to 1.00), which parallels the aforementioned human study; inoculation of Hepatitis C and Hepatitis E in chimpanzees (Figure S4; Baltimore group IV; AUC 0.96-1.00); and infection of macaque monkeys with Marburg virus (Figure S5; Baltimore group V; AUC 0.98). Performance of the pan-viral signature was strong in all of these additional validation datasets, as indicated in Table 3 and in the Supplementary Figures.

### Additional validation from clinical studies

The pan-viral signature was also tested in two clinical studies that were conducted to determine the signature’s ability to differentiate patients with virus-associated SI from those with SI due to other etiologies, including bacterially- and surgically-induced SI. Gene expression levels were inferred from RNA sequencing (RNA-seq) data obtained from whole blood samples collected in PAXgene blood RNA tubes.

#### Internal Validation Dataset #1: FEVER study

**Figure 7:**
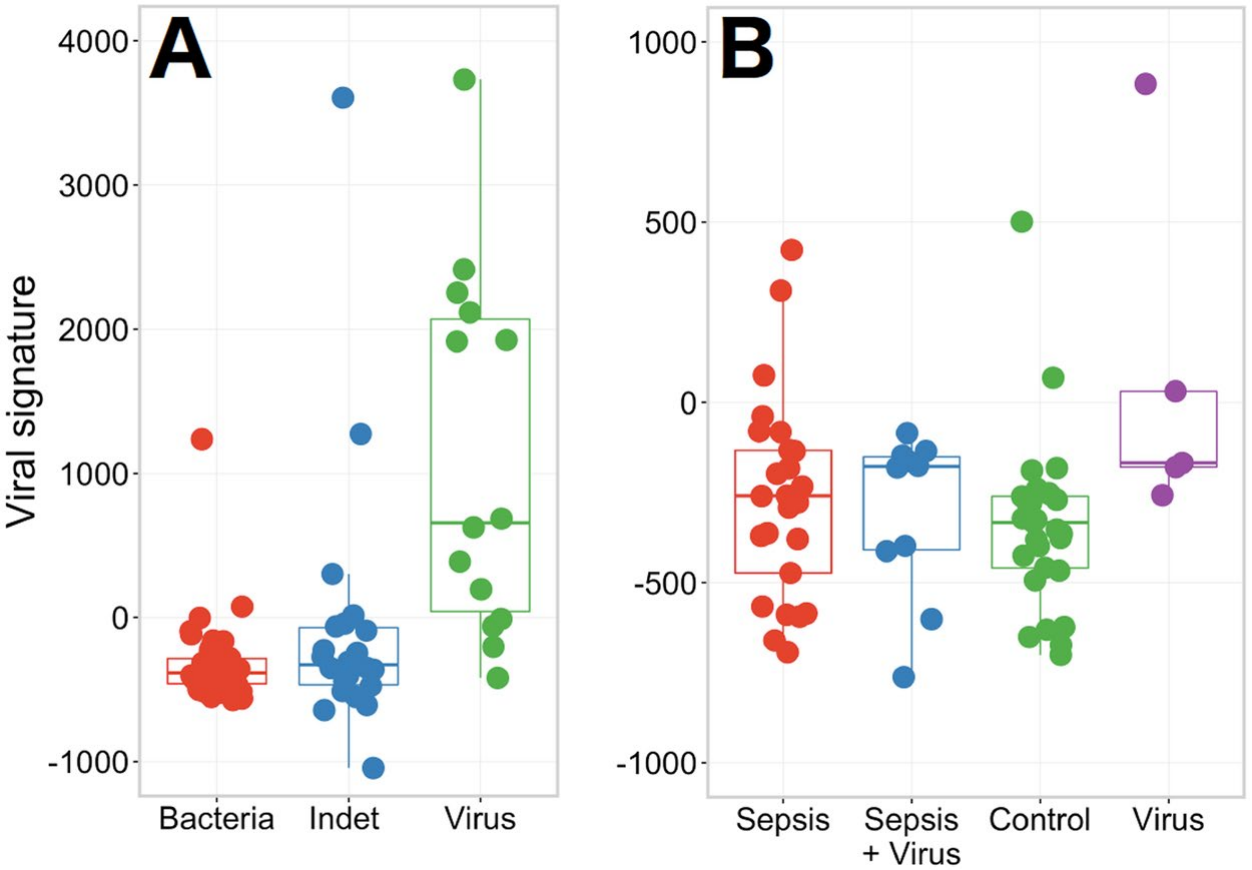
Pan-viral signature score in two clinical studies. Panel (A): Adult patients presenting to the emergency department with fever. Box and whisker plots for 90 patients in the FEVER study retrospectively diagnosed with bacterial sepsis (n = 54; red points), indeterminate status (Indet, n = 22; blue points), or viral infection (n = 14; green points). Panel (B): Pediatric intensive care patients. Box-and-whisker plot of pan-viral signature scores for 69 children retrospectively diagnosed as sepsis (n = 25), sepsis with an identified viral coinfection (Sepsis + Virus, n = 10), post-surgical systemic inflammation (Control, n = 29) and viral-associated systemic inflammation (Virus, n = 5).

This study involved adult patients presenting to a UK emergency department with fever (see the Supplementary Text S1, Figure S6 and Table S1 for study details, and Table S2 for line data). All patients included in the study were admitted to hospital and received retrospective physician diagnosis (RPD), using all available clinical information at discharge, including any results of clinical microbiology and virus testing, to determine the presumptive etiology of the fever. Of the 90 patients comprising the FEVER study cohort, those with confirmed bacterial infections (N = 54) were identified by microbial culture of pathogenic bacteria from sterile sites. Confirmed viral infections (N = 14) were identified by positive nucleic acid detection or serological tests as ordered by the attending clinician (see Text S1). Patients who had no positive microbiological tests and recovered without empirical antimicrobial treatment (N = 22) were designated as indeterminate cases. Positively identified viruses in the ‘virally infected’ patients included Baltimore group I (herpes virus, varicella-zoster virus, Epstein-Barr virus, cytomegalovirus), Baltimore group IV (dengue virus), and Baltimore group V (Influenza A and B viruses). Figure 7, panel A shows box and whisker plots of the pan-viral signature, assayed in blood samples from the three patient groups. The pan-viral signature effectively separated febrile patients of confirmed viral etiology from those of confirmed bacterial etiology with AUC 0.93. All patients in this study had a fever (temperature >38.5 °C) at the time of presentation and blood sampling. The fact that the indeterminate cases recovered spontaneously may be most consistent with self-limiting viral illnesses, but interestingly only 2–3 of 22 indeterminate cases had pan-virus signature scores significantly higher than the proven cases of bacterial infection, suggesting that the majority of these indeterminate cases did not represent acute viral infections.

#### Internal Validation Dataset #2: GAPPSS study

A second clinical study^40^ (clinicaltrials.gov reference # NCT02728401) was undertaken that involved pediatric patients (age range: 38 weeks estimated gestational age – 18 years) in intensive care (see Supplementary Text S2 and Table S3 for study details, and Table S4 for line data). Using all available clinical information, including clinical microbiology and virus testing, the patients were retrospectively diagnosed with bacterial sepsis (n = 25), bacterial sepsis with a viral coinfection (n = 10), viral SI (n = 5), or sterile post-surgical SI (n = 29). Testing of respiratory samples from the cohort, using the BioFire FilmArray Respiratory Panel (Biofire Diagnostics, Utah, USA), identified viruses in Baltimore group I (varicella-zoster virus; herpes simplex virus), Baltimore group IV (rhinovirus/enterovirus; coronavirus HKU1; norovirus Type 2) and Baltimore group V (parainfluenza 3; respiratory syncytial virus; metapneumovirus). Results are displayed graphically in Fig. 7, Panel B and summarized in Table 3.Whilst only a limited number of viral patients were included in this study (n = 5), the pan-viral signature resolved viral SI from non-infectious SI with AUC 0.91, and resolved viral SI from bacterial sepsis with AUC 0.76. Similar to our observation in the adult study (Fig. 7, panel A above), the pan-viral signature was much less effective at separating bacterial sepsis from non-infectious SI (AUC 0.60) demonstrating that the signature is specific for viral systemic inflammation and not bacterial systemic inflammation. Discordance between RPD and the pan-viral score in some cases suggests the possibility that either some patients had undetected viral infections, that the pan-viral signature had reduced specificity in children, or the study was not sufficiently powered to draw definitive conclusions.

### Resolution of viral vs. bacterial SI using two specific signatures

We have previously discovered and validated a four-gene host response signature (*SeptiCyte* ^*TM*^ *LAB*) for differentiating SI due to either bacterial or non-infectious etiology^41^. Given that the pan-viral signature was developed to be specific for discrimination of viral vs. non-infectious SI, and appears to be largely unaffected by bacterial infection, we hypothesized it would be possible to apply the two signatures simultaneously to allow a three-way discrimination between non-infectious SI, viral SI, and bacterial SI.

**Figure 8:**
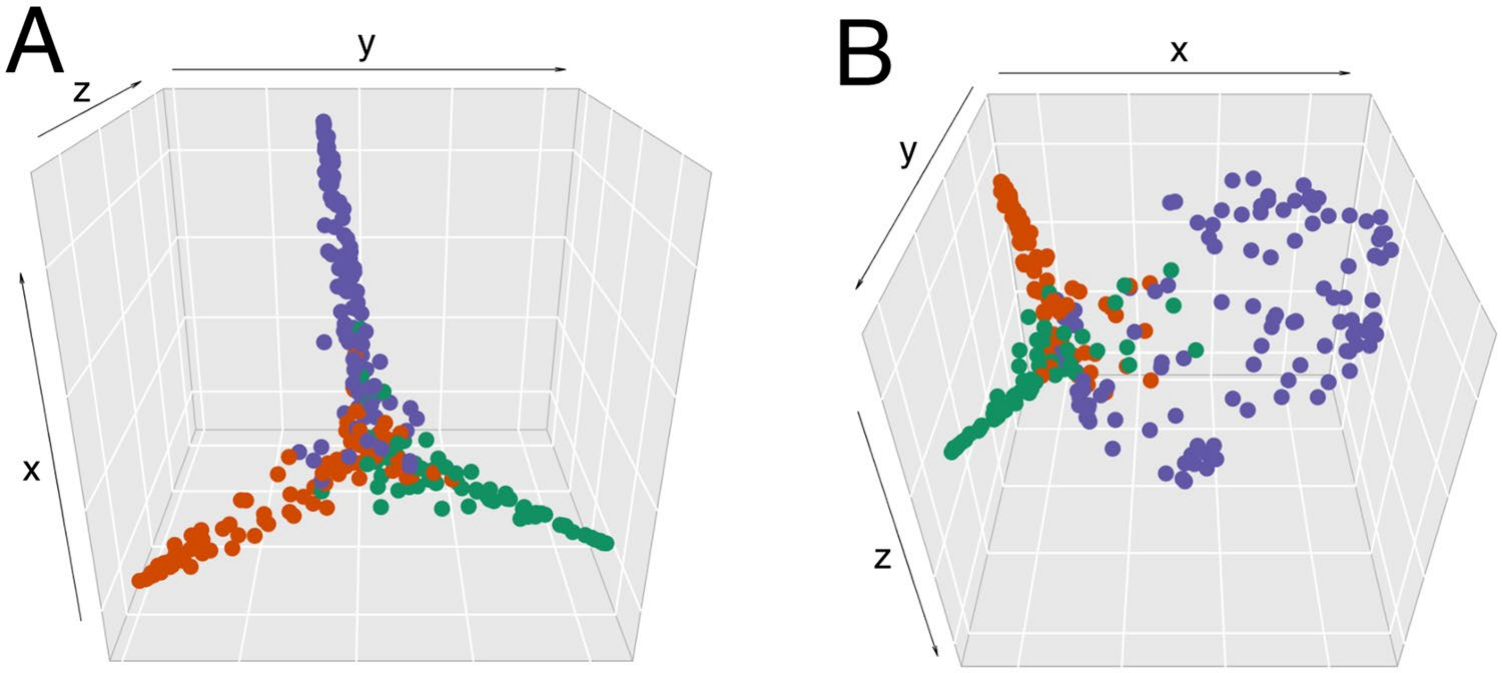
Resolution of patients with acute respiratory illness (ARI) into three clusters corresponding to bacterial infection, viral infection, and non-infectious illness (GSE63990). A cohort (GSE63990) having multiple types of pathogen infections has been analyzed using a Random Forest - multidimensional scaling (RF-MDS) process that combines the pan-viral signature and the *SeptiCyte* ^*TM*^ *LAB* signature^41^. Panel (A): A three-dimensional projection of points from an 8-dimensional space defined by the expression levels of the 8 individual genes comprising the two signatures. This projection was chosen to show maximal visual separation between the three clinical groups (bacterial, viral, and non-infectious illness). Panel (B): Another three-dimensional projection, chosen to show the relatively high dispersion of the virally infected samples. Key: bacterial, green points; viral, purple points; non-infectious illness, orange points.

As an initial test of this hypothesis, we reanalyzed a dataset (GSE63990) from a study^42^ of patients with acute respiratory illness (ARI). This study enrolled 273 patients of which 115, 70 and 88 received retrospective clinical diagnoses of bacterial infection, viral infection, and non-infectious illness, respectively. We analysed GSE63990 using an 8-gene classifier consisting of the four pan-viral signature genes (*IL16, ISG15, OASL, ADGRE5*) combined with the four genes (*CEACAM4, LAMP1, PLA2G7, PLAC8*) from *SeptiCyte* ^*TM*^ *LAB*. The line data used in our analysis is given in Supplementary Table S5. We applied a Random Forest - multidimensional scaling (RF-MDS) analysis^43–45^ using the combined eight genes. Figure 8 (Panels A, B) presents two different visual representations of the analysis, which show that the GSE63990 dataset has been resolved into the three patient subgroups of bacterial infection (green), viral infection (purple), and non-infectious illness (orange). An animated representation of this analysis, in which the figure is rotated in three dimensions, is provided in Supplementary Animation S1. To assess whether these 8 genes were contributing materially to the underlying biology, and thus to the clinical diagnoses of viral, bacterial or non-infectious illness, we used the resampling method described by Li *et al*.^46^ and created 2,000 permutations of GSE63990 in which the group labels were randomly shuffled. Application of the Random Forest model to the permuted datasets failed to resolve the three groups, after group label randomization. Thus the classifier was found to be significant under the null hypothesis. That is, the results presented in Fig. 8 illustrate a true dependency between the 8 genes and the response labels, at a significance level of p < 0.001. Additional details of the permutation test are provided in Supplementary Figures S7 and S8.

We note the GSE63990 dataset was not used in the initial discovery or validation of either the pan-viral signature or *SeptiCyte* ^*TM*^ *LAB* signature. Also, the possibility of bacterial or viral co-infection was not considered in our analysis. Furthermore, the diagnostic performance of *SeptiCyte* ^*TM*^ LAB and the pan-viral signature is dependent upon the accuracy of retrospective physician diagnoses of acute respiratory illness cases. There is some degree of discordance between the retrospective physician diagnoses and our two signatures, a finding that was also reported in the original publication^42^ when classifiers reported in that paper were used (35 of 273 patients had a discordant result (12.8%)). Clearly further validation work is required to demonstrate the clinical utility of combining both signatures, but these data provide a valuable insight into the potential of an assay that combines viral and bacterial host responses.

## Discussion

In this paper we identify and validate a peripheral-blood signature based on the expression of four genes (*ISG15, OASL, IL16, ADGRE5*), which exhibits high AUC for discriminating viral from bacterial and non-infectious causes of SI. This signature has been validated using publicly available GEO datasets, and in our own clinical studies in adults and children. We have termed the signature “pan-viral” because it has demonstrable diagnostic power across six mammalian species (human, macaque, chimpanzee, pig, rat and mouse), in multiple tissue types, *in vivo* and *in vitro*, and in infections caused by viruses representing all seven Baltimore classification groups.

Because the direct sensing of different classes of viruses is mediated through different Pathogen-Associated Molecular Patterns ﻿(PAMPs), we hypothesize that the pan-viral signature most likely reflects some type of integrated downstream response^47, 48^. A plausible hypothesis regarding the functional significance of three of the genes in the pan-viral signature (*ISG15, OASL, IL16*) is that they relate to type 1 interferon signaling. *ISG1*5, a well-studied component of the type 1 interferon-mediated response to viral infection, is a mediator of ISGylation, a protein modification similar to ubiquination^49–52^. *OASL* is a non-enzymatic member of the highly conserved OAS gene family^53^ and is also a component of the Type 1 interferon response to viral infection^54, 55^. *IL16* is a cytokine with multiple functions, having been linked to inhibition of HIV-1 infection^56, 57^, modulation of HBV infection^58^, lentiviral infection^59^ and autoimmune and allergic disorders^60, 61^. A paper from some years ago^62^ demonstrated that IFN-α induces the secretion of IL-16 by several cell types. A more recent paper^63^ reported a negative effect of IFN-β1a (a type 1 interferon) on the expression level of IL-16; thus IL16 may also be functionally related to the Type 1 interferon pathway, although the linkage is not especially well studied or documented. Finally, although *ADGRE5* has not been linked to interferon Type 1 signaling, this gene has previously been directly associated with host response to infection by human papilloma virus^64^ and HIV^65^. Additionally, the *ADGRE5* ligand DAF (decay accelerating factor) is the cellular receptor for both echovirus^66, 67^ and coxsackie virus^68, 69^.

Context for our work is found in prior studies describing transcriptional signatures that were designed to distinguish between some viral, bacterial, and non-infectious SI conditions. However, we have found that prior work was limited by either a large number of genes/probes required, a lack of specificity of the signatures in light of other possible causes of SI, or a lack of validation across a broad range of virus types. For example, Zaas *et al*.^19^ identified a 30-gene signature from microarray analysis of symptomatic vs. asymptomatic subjects infected with rhinovirus, respiratory syncytial virus, or influenza A; this signature was able to discriminate symptomatic influenza A-infected subjects from both healthy subjects and bacterially-infected subjects in a second independent cohort. Other researchers^17, 18, 20, 70^ have described signatures for discriminating between viral infections and other conditions, but with limitations relating to the large number of biomarkers in the signature (>18), a limited number of viruses examined, or a lack of demonstrated specificity with respect to possible bacterial co-infection or SI due to non-infectious causes. Tsalik *et al*.^42^ identified host gene expression signatures for viral, bacterial and non-infectious causes of acute respiratory inflammation. Whilst respiratory illness accounts for a large proportion of patients presenting to emergency clinics, the viral signature identified in this study consisted of a large number of genes (n = 33) and was not validated on patients with SI as a result of viral infection of body systems other than respiratory. Sweeney *et al*.^22, 71^ described an 11-gene signature for differentiating infectious and non-infectious SI, and also a 7-gene signature for differentiating bacterial and viral SI, but not non-infectious SI. Used in succession the authors claimed that such signatures could be used as an “integrated antibiotics decision model”. Finally, Herberg *et al*.^23^ described a two-gene signature for differentiating viral and bacterial infection in febrile children. This signature was developed without using a cohort of non-infectious SI and therefore the output is binary and assumes that patients have a viral or bacterial infection.

Our approach to discovery of host response viral biomarkers is novel in comparison to the prior studies because we have: (1) included representative pathogenic viruses from all seven Baltimore viral classification groups, thus providing evidence that innate immune response commonalities may be potentially harnessed for broad diagnostic utility across diverse viral infection; (2) incorporated datasets from multiple mammalian species to demonstrate the robustness that host response -based methods offer; (3) used non-infectious SI as our control group, recognizing the fact that discriminating viral, bacterial, and non-infectious causes of SI is a highly critical and difficult distinction to make on the basis of clinical features alone^6, 41, 71, 72^; (4) applied a comprehensive specificity screen to eliminate biomarkers that respond to potentially confounding medical conditions or demographic variables; and (5) applied strong selection pressure towards minimizing the number of biomarkers in the pan-viral signature to avoid overfitting and to enable a straightforward conversion to a practical assay format, such as a format employing reverse transcription - quantitative polymerase chain reaction (RT-qPCR).

A number of the discovery and validation datasets in our study (Tables 1 and 3 respectively) were derived from time course and/or challenge experiments. The use of such datasets is important because samples taken from subjects early in the viral pathogenesis, or from otherwise healthy subjects undergoing vaccine challenge, are most likely to reflect an infection with a single type of virus, rather than an infection with multiple virus types, or co-infection with bacteria. Analysis of the time-course datasets revealed that, in general, it took up to three days post-exposure for the pan-viral signature to first register a significant difference compared to pre-exposure samples; the pan-viral signature response coincided with the ability to first detect virus in tissue but preceded viremia, clinical signs and antibody response.

Our study has several limitations. First, the validation datasets we employed were generated from multiple sample types (blood, liver biopsy, cultured hepatocytes) using multiple experimental methods (microarrays, RNA-seq). This diversity of sample types and methods could contribute a significant amount of noise which would tend to obscure relevant signals. Once the assay has been translated to a single assay technology and sample type, then more precise comparisons between different viral infections and disease severities can be made. Second, Baltimore Group II is under-represented in our validation data. The dataset that we analyzed (GSE14790, porcine circovirus infection) did not include OASL. Because the genes comprising the pan-viral signature were discovered by a process in which gene pairs were linearly combined, we present results for the linear combination of ISG15 and IL16, which still carries significant diagnostic power in the cohort tested (GSE14790). We expect that eventually additional Baltimore group II datasets will become available, which will allow a more in-depth validation of the pan-viral signature performance in this viral group.

Third, the FEVER and GAPPSS studies we have described in Fig. 7 are limited with respect to the size of the viral infection groups (n = 14 for FEVER, and n = 5 for GAPPSS). These studies are ongoing, and additional recruitment is expected over the coming months.

Fourth, definitive clinical utility of the pan-viral signature remains to be determined. Our observations from a variety of validation datasets suggest that the pan-viral signature could potentially have multiple clinical applications: as an early diagnostic tool, in monitoring recovery from viral infections, in monitoring host response to therapeutic interventions, in monitoring host response to vaccines, and/or in surveillance of populations at risk. For example, in combination with a bacterial signature that has inherent high negative predictive value, the pan-viral signature could potentially be a useful tool in an antibiotic stewardship program, or in providing guidance for ongoing diagnostic testing. It could also prove useful in identifying patients early in the course of a viral infection, which in turn could affect decisions on infection control and patient isolation, especially in disease outbreaks. Additional clinical studies will be needed to determine if the pan-viral signature has clinical utility for these or other purposes.

We believe a particular strength of our discovery approach was the resultant specificity of the pan-viral signature when compared to bacterial and non-infectious causes of SI. Such specificity allows this signature to be combined with our *SeptiCyte* ^*TM*^ *LAB* signature, which has specificity for bacterial SI. The combination of virus-specific and bacteria-specific host SI signatures may provide clinicians with timely information to aid in informed decision making in patients presenting with SI, for example in deciding whether to initiate or cease antibiotics. Ultimately, clinical utility for a “pan-viral” signature may be found in combination with an infection status classifier, like that we have previously described^40^ whereby together, both the probability of systemic infection, along with infection type (i.e. bacterial vs. viral) can be rapidly determined and factored into patient management and treatment decisions.

## Methods

### Statistical Tests

Several different statistical tests were used to evaluate the performance of classifiers. (1) When sufficient numbers of samples were available, ROC curve analysis was performed and AUCs were calculated. A resampling method was used to estimate the AUC 95% confidence interval (CI) associated with each ROC curve. Venkatraman’s method^73^, as implemented in the pROC package in R, was used to compare the AUC values between different biomarker combinations with p < 0.05 considered statistically significant. (2) For some performance estimates the Mann-Whitney U test was used, which gives an equivalent statistic to AUC^74^. (3) For some analyses with very small sample sizes, Student’s t-test was used, following appropriate small-sample guidelines^75^.

### Discovery of the pan-viral signature

In the discovery phase we searched for RNA transcripts or transcript combinations with expression levels that varied during a host response to viral infection. The initial search was conducted across 13 datasets from human adult and pediatric subjects, plus one set of data from macaques. We expected there to be some variability between datasets in quantification of the levels of particular RNA transcripts because different studies used different sample types, sample collection tubes, experimental platforms (microarrays, RNA-seq), and data reduction/processing methods to estimate gene expression levels. A considerable literature has arisen on comparing gene expression results across platforms^76–79^ and on estimating the biases that may arise specifically within microarray-based approaches^80, 81^ and RNA-seq -based approaches^82–84^. For each GEO dataset, we represented each gene’s RNA transcript family by the single microarray probe that gave the maximal average intensity for that gene, across all samples used in the analysis. Probe identities are listed in Supplementary Table S6.

We began the search using four core datasets (GSE40366, GSE51808, GSE52428 and GSE41752). To decrease the dimensionality of the search space and to ensure that only those transcripts with moderate to high expression levels were examined, we applied a mean expression filter that allowed only the top 6,000 RNA transcripts from each of the core datasets to be retained. Regression analysis was then applied across the search space, with RNA transcripts combined in pairs, using a linear objective function with coefficients set to −1 or +1 for the log_2_ expression value of each transcript in a pair. In theory, each core dataset produced 36,000,000 transcript pairs to examine (not taking into account reciprocal pairs). Setting the coefficients to −1 or +1 (instead of allowing the coefficients to vary) reduced the computational effort to a manageable level. ROC curve analysis on each transcript pair then allowed the transcript pairs to be ranked by AUC for their ability to separate the case and control groups in each of the core datasets.

The RNA transcript pairs were then filtered by the following two-step process: (1) those with average AUC <0.92 across the four core datasets were discarded; and (2) those with average AUC < 0.92 across ten additional viral-based “sensitivity” datasets (Table 1) were discarded. This resulted in a severely reduced pool of transcript pairs (N = 856) with AUC ≥ 0.92. Next, the four “core” and ten “sensitivity” datasets (Table 1) were individually normalized, as follows. (1) The mean expression level of each RNA transcript was calculated across all samples in that dataset. (2) The expression level of this transcript in each sample was then adjusted by subtracting its mean value. (3) All expression values were then scaled to unit variance. This procedure was performed for every transcript in each individual dataset. All 14 viral datasets were then merged into a single expression matrix.

### Specificity screen with independent GEO datasets

To ensure that candidate transcript pairs were associated uniquely with a viral host response and not a host response due to confounding phenotypes, they were individually assessed against 19 “specificity” datasets. The specificity datasets were derived from bacterial-positive patients, some of whom were classified as septic (GSE3341, GSE16129, GSE40396), patients with SIRS (GSE40012), healthy subjects ranging in age from childhood to nonagenarian (GSE40366), patients with inflammation not associated with positive viral infection (GSE42834, GSE17755, GSE19301, GSE47655, GSE38485, GSE36809, GSE29532, GSE61672), neonatal and pediatric bacterial sepsis patients (GSE25504, GSE30119, GSE6269), patients with anxiety (GSE61672), subjects administered dexamethasone (GSE46743), and healthy subjects displaying demographic confounders such as age, ethnicity and gender (GSE35846). Candidate transcript pairs having AUC >0.80 in more than 3 of the 19 specificity datasets were discarded. A total of 473 candidate transcript pairs remained after this step.

### Final selection step

Finally, a greedy forward search was performed on the reduced pool of highest-ranked RNA transcript pairs according to previously described methods^41^. The end product of this search was the final pan-viral signature containing two upregulated and two down regulated RNA transcripts as a linear sum (*ISG15* + *OASL*) - (*IL16* + *ADGRE5*).

### Validation in independent GEO datasets

The pan-viral signature was then tested against 11 independent “validation” datasets (Table 3). These datasets were derived from six mammalian species (human, macaque, chimpanzee, pig, mouse and rat), all seven Baltimore groups, and various tissue types (blood, liver biopsies, *in vitro* primary hepatocytes), and included time course and vaccination studies in humans. It should be noted that differences in the y-axis scale (pan-viral signature value) between various studies, as indicated in figures in the text and Supplementary Material, result from differences in the various gene expression measurement platforms across studies.

### Validation in independent clinical studies

The pan-viral signature received additional validation from two independent clinical studies, FEVER and GAPPSS, which were conducted on adult and pediatric patients respectively. Details of the FEVER study are provided in Supplementary Tables S1 and S2, Figure S6 and Text S1, and details of the GAPPSS study are provided in Supplementary Tables S3 and S4, Text S2, and the publication by Zimmerman *et al*.^40^ The GAPPSS study was an institutional review board-approved prospective, observational study (Seattle Children’s Hospital IRB #14761). Parental informed permission was obtained prior to sample and data collection. All sample and data collection was carried out in accordance with approved protocols and procedures. The FEVER study was also an institutional review board-approved prospective, observational study (UK National Research Ethics services reference number: 09/H0701/103). All participants provided written informed consent, prior to sample and data collection. All sample and data collection was carried out in accordance with approved protocols and procedures.

The FEVER study cohort consisted of adult patients presenting with fever to the Emergency Department, and then admitted to hospital. A comparison was made between those retrospectively diagnosed with a viral infection (n = 15), with bacterial sepsis (n = 55) or with infection-negative SI (n = 22). In the FEVER study, testing for viral infections was only performed on those patients suspected of a viral infection, and involved use of one or more single-virus diagnostic tests based on the clinician’s judgment and according to hospital procedures^85^ (e.g. PCR for influenza, serology for dengue, etc.). The GAPPSS study cohort consisted of pediatric intensive care patients retrospectively diagnosed with a viral infection (n = 5), bacterial sepsis (n = 25), or bacterial sepsis with a viral co-infection (n = 10), as well as infection-negative SI controls undergoing cardio-pulmonary bypass surgery (n = 29). All patients in the GAPPSS study, except for one bacterial sepsis patient who was omitted from the analysis, were tested for the presence of viral nucleic acid sequences in nasal swabs using the Biofire FilmArray Respiratory Panel (Biofire Diagnostics, Utah, USA). Supplementary Tables S3 and S4 present the relative gene expression values for *ISG15, IL16, OASL, ADGRE5* derived from RNA-seq data for the FEVER and GAPPSS patients, respectively. For each of the two datasets (FEVER or GAPPSS), we represented the expression level of a gene of interest by Fragments Per Kilobase of transcript per Million mapped reads (FPKM)^86^. This measure of gene expression should be independent of whether the data are in the form of single-end reads (FEVER) or paired-end reads (GAPPSS).

### Combination of *SeptiCyte™ LAB* and pan-viral signature

To demonstrate utility of a combined bacterial and viral host response assay, we analysed GEO dataset GSE63990 using an 8-gene classifier consisting of the four pan-viral signature genes (*IL16, ISG15, OASL, ADGRE5*) combined with the four genes (*CEACAM4*, *LAMP1*, *PLA2G7, PLAC8*) from *SeptiCyte* ^*TM*^ *LAB*. The class labels used in GSE63990 were: bacterial infection, viral infection, and non-infectious illness. Line data from GSE63990 are presented in Supplementary Table S5. To assess whether a significant biological response exists from the eight genes, we performed a permutation test. Under this statistical framework the dependency between the feature space and the response (class labels) is broken thus allowing us to understand the behavior of the model under the null hypothesis that the explanatory variables and response labels are independent. The model, in this case, consisted of a supervised Random Forest analysis^43^ constructed from 1000 trees and allowing √*f* features to be selected randomly at each split, where *f* = *8* and represents the number of gene targets. The class labels were then randomly permuted 2,000 times which allowed for a 0.05 alpha level with a 0.01 precision^87^. The data were then modeled using Random Forests. For each null model the multiclass log-loss was calculated to construct the null distribution before assessing the true response labels against the final null model.

## Electronic supplementary material

Supplementary Material

Supplementary Animation S1

## References

[1] Comstedt P, Storgaard M, Lassen AT (2009). The Systemic Inflammatory Response Syndrome (SIRS) in acutely hospitalised medical patients: a cohort study. Scand. J. Trauma Resusc. Emerg. Med..

[2] Pavare J, Grope I, Gardovska D (2009). Prevalence of systemic inflammatory response syndrome (SIRS) in hospitalized children: a point prevalence study. BMC Pediatr..

[3] Munro N (2014). Fever in acute and critical care: a diagnostic approach. AACN Adv. Crit. Care.

[4] Niska R, Bhuiya F, Xu J (2010). National hospital ambulatory medical care survey: 2007 emergency department summary. Natl. Health Stat. Report.

[5] Braykov NP (2014). Assessment of empirical antibiotic therapy optimisation in six hospitals: an observational cohort study. The Lancet Infectious Diseases.

[6] Coburn B, Morris AM, Tomlinson G, Detsky AS (2012). Does this adult patient with suspected bacteremia require blood cultures?. JAMA.

[7] Centers for Disease Control and Prevention (CDC). Antibiotic resistance threats in the United States, 2013. Atlanta: CDC. http://www.cdc.gov/drugresistance/threat-report-2013/pdf/ar-threats-2013-508.pdf (2013).

[8] Hament JM, Kimpen JL, Fleer A, Wolfs TF (1999). Respiratory viral infection predisposing for bacterial disease: a concise review. FEMS Immunol. Med. Microbiol..

[9] Zaas AK (2009). Gene expression signatures diagnose influenza and other symptomatic respiratory viral infections in humans. Cell Host & Microbe.

[10] Zhai Y (2015). Host transcriptional response to influenza and other acute respiratory viral infections – a prospective cohort study. PLOS Pathog..

[11] Storch GA (2000). Diagnostic virology. Clin. Infect. Dis..

[12] Cobo F (2012). Application of molecular diagnostic techniques for viral testing. Open Virol. J..

[13] Jansen RR (2011). Frequent detection of respiratory viruses without symptoms: toward defining clinically relevant cutoff values. J. Clin. Microbiol..

[14] Yu C (2010). Pathogenesis of hepatitis E virus and hepatitis C virus in chimpanzees: similarities and differences. J. Virol..

[15] Huang Y (2011). Temporal Dynamics of Host Molecular Responses Differentiate Symptomatic and Asymptomatic Influenza A Infection. PLOS Genet..

[16] Parnell GP (2012). A distinct influenza infection signature in the blood transcriptome of patients with severe community-acquired pneumonia. Crit. Care.

[17] Hu X, Yu J, Crosby SD, Storch GA (2013). Gene expression profiles in febrile children with defined viral and bacterial infection. Proc. Natl. Acad. Sci. USA.

[18] Woods CW (2013). A host transcriptional signature for presymptomatic detection of infection in humans exposed to influenza H1N1 or H3N2. PLOS ONE.

[19] Zaas AK (2013). A host-based RT-PCR gene expression signature to identify acute respiratory viral infection. Sci. Transl. Med..

[20] Andres-Terre M (2015). Integrated, multi-cohort analysis identifies conserved transcriptional signatures across multiple respiratory viruses. Immunity.

[21] Heinonen S (2016). Rhinovirus detection in symptomatic and asymptomatic children: value of host transcriptome analysis. Am. J. Respir. Crit. Care Med..

[22] Sweeney TE, Wong HR, Khatri P (2016). Robust classification of bacterial and viral infections via integrated host gene expression diagnostics. Sci. Transl. Med..

[23] Herberg JA (2016). Diagnostic test accuracy of a 2-transcript host RNA signature for discriminating bacterial vs viral infection in febrile children. JAMA.

[24] Robinson, C. & M. Echavarria, M. Adenoviruses In *Manual of Clinical Microbiology*, *9*^*th*^*edition* (ed. Murray, P.R. *et al*.) 1589 (ASM Press, 2007).

[25] Wold, W. S. M. & Horwitz, M. S. Adenoviruses In *Fields Virology, 5*^*th*^*edition* (eds. Knipe D. M. & Howley, P. M.) 2395–2436 (Lippincott Williams & Wilkins, 2007).

[26] Lenaerts L, De Clercq E, Naesens L (2008). Clinical features and treatment of adenovirus infections. Revs. Med. Virol..

[27] Flomenberg P (2009). Adenovirus infections. Medicine.

[28] Kiang A (2006). Multiple innate inflammatory responses induced after systemic adenovirus vector delivery depend on a functional complement system. Mol. Ther..

[29] Eskildsen S, Justesen J, Schierup MH, Hartmann R (2003). Characterization of the 2′–5′-oligoadenylate synthetase ubiquitin-like family. Nucleic Acids Res..

[30] Tomas A, Fernandes LT, Sanchez A, Segales J (2012). Time course differential gene expression in response to porcine circovirus type 2 subclinical infection. Vet. Res..

[31] Yen C (2014). Rotavirus vaccines. Human Vaccines.

[32] Tsuge M (2014). Gene expression analysis in children with complex seizures due to influenza A(H1N1)pdm09 or rotavirus gastroenteritis. J. Neurovirol..

[33] Daep CA, Muñoz-Jordán JL, Eugenin EA (2014). Flaviviruses, an expanding threat in public health: focus on dengue, West Nile, and Japanese encephalitis virus. J. Neurovirol..

[34] Garske T (2014). Yellow fever in Africa: estimating the burden of disease and impact of mass vaccination from outbreak and serological data. PLOS Med..

[35] Gaucher D (2008). Yellow fever vaccine induces integrated multilineage and polyfunctional immune responses. J. Exp. Med..

[36] Nair H (2010). Global burden of acute lower respiratory infections due to respiratory syncytial virus in young children: a systematic review and meta-analysis. Lancet.

[37] Brand HK (2015). Olfactomedin 4 serves as a marker for disease severity in pediatric respiratory syncytial virus (RSV) infection. PLOS ONE.

[38] Skinner J (2009). P01-01. The blood transcriptional response to early acute HIV infection is transient and responsive to antiretroviral therapy. Retrovirology.

[39] Lamontagne J, Mell JC, Bouchard MJ, Siddiqui A (2016). Transcriptome-wide analysis of hepatitis B virus-mediated changes to normal hepatocyte gene expression. PLOS Pathog..

[40] Zimmerman JJ (2017). Diagnostic accuracy of a host gene expression signature that discriminates clinical severe sepsis syndrome and infection-negative systemic inflammation among critically ill children. Crit. Care. Med..

[41] McHugh L (2015). A molecular host response assay to discriminate between sepsis and infection-negative systemic inflammation in critically ill patients: discovery and validation in independent cohorts. PLOS Med..

[42] Tsalik EL (2016). Host gene expression classifiers diagnose acute respiratory illness etiology. Sci. Transl. Med..

[43] Breiman L (2001). Random forests. Mach. Learn..

[44] Mardia KV (1978). Some properties of classical multidimensional scaling. Commun. Stat. Theory Methods A.

[45] Cox, T. F. & Cox, M. A. A. Multidimensional Scaling, *2*^*nd*^*edition* (Chapman and Hall, 2001).

[46] Li J (2010). Identification of high-quality cancer prognostic markers and metastasis network modules. Nat. Commun..

[47] Brennan K, Bowie AG (2010). Activation of host pattern recognition receptors by viruses. Curr. Opin. Microbiol..

[48] Thompson MR, Kaminski JJ, Kurt-Jones EA, Fitzgerald KA (2011). Pattern recognition receptors and the innate immune response to viral infection. Viruses.

[49] Ritchie KJ (2004). Role of ISG15 protease UBP43 (USP18) in innate immunity to viral infection. Nat. Med..

[50] Malakhova OA, Zhang DE (2008). ISG15 inhibits Nedd4 ubiquitin E3 activity and enhances the innate antiviral response. J. Biol. Chem..

[51] Chen L, Li S, McGilvray I (2011). The ISG15/USP18 ubiquitin-like pathway (ISGylation system) in hepatitis C virus infection and resistance to interferon therapy. Int. J. Biochem. Cell Biol..

[52] Zhang X (2014). Human intracellular ISG15 prevents interferon-α/β over-amplification and auto-inflammation. Nature.

[53] Choi UY, Kang J-S, Hwang YS, Kim Y-J (2015). Oligoadenylate synthase-like (OASL) proteins: dual functions and associations with diseases. Exp. Mol. Med..

[54] Schoggins JW (2011). A diverse range of gene products are effectors of the type I interferon antiviral response. Nature.

[55] Strouts FR (2016). Early transcriptional signatures of the immune response to a live attenuated tetravalent dengue vaccine candidate in non-human primates. PLOS Negl. Trop. Dis..

[56] Baier M, Werner A, Bannert N, Metzner K, Kurth R (1995). HIV suppression by interleukin-16. Nature.

[57] Truong MJ (1999). Interleukin-16 inhibits human immunodeficiency virus type 1 entry and replication in macrophages and in dendritic cells. J Virol..

[58] Romani S (2014). Interleukin-16 gene polymorphisms are considerable host genetic factors for patients’ susceptibility to chronic hepatitis B infection. Hepat. Res. Treat..

[59] Nimmanapalli R, Sharmila C, Reddy PG (2010). Immunomodulation of caprine lentiviral infection by interleukin-16. Comp. Immunol. Microbiol. Infect. Dis..

[60] Glass WG, Sarisky RT, Vecchio AM (2006). Not-so-sweet sixteen: the role of IL-16 in infectious and immune-mediated inflammatory diseases. J. Interferon Cytokine Res..

[61] Bowler RP (2013). Integrative omics approach identifies interleukin-16 as a biomarker of emphysema. OMICS.

[62] Ludwiczek O (2001). Activation of caspase-3 by interferon alpha causes interleukin-16 secretion but fails to modulate activation induced cell death. Eur. Cytokine Netw..

[63] Nischwitz S (2014). Interferon β-1a reduces increased interleukin-16 levels in multiple sclerosis patients. Acta. Neurol. Scand..

[64] Santin AD (2005). Gene expression profiles of primary HPV16- and HPV18-infected early stage cervical cancers and normal cervical epithelium: identification of novel candidate molecular markers for cervical cancer diagnosis and therapy. Virology.

[65] Zhou H (2008). Genome-scale RNAi screen for host factors required for HIV replication. Cell Host Microbe.

[66] Sobo K, Rubbia-Brandt L, Brown TD, Stuart AD, McKee TA (2011). Decay-accelerating factor binding determines the entry route of echovirus 11 in polarized epithelial cells. J. Virol..

[67] Plevka P (2010). Interaction of decay-accelerating factor with echovirus 7. J. Virol..

[68] S. Hafenstein S (2007). Interaction of decay-accelerating factor with coxsackievirus B3. J. Virol..

[69] Yoder JD, Cifuente JO, Pan J, Bergelson JM, Hafenstein S (2012). The crystal structure of a coxsackievirus B3-RD variant and a refined 9-angstrom cryo-electron microscopy reconstruction of the virus complexed with decay-accelerating factor (DAF) provide a new footprint of DAF on the virus surface. J Virol..

[70] Ramilo O (2007). Gene expression patterns in blood leukocytes discriminate patients with acute infections. Blood.

[71] Sweeney TE, Shidham A, Wong HR, Khatri P (2015). A comprehensive time-course-based multicohort analysis of sepsis and sterile inflammation reveals a robust diagnostic gene set. Sci Transl. Med..

[72] Han JH (2015). Use of a combination biomarker algorithm to identify medical intensive care unit patients with suspected sepsis at very low likelihood of bacterial infection. Antimicrob. Agents Chemother..

[73] Venkatraman ES (2000). A permutation test to compare receiver operating characteristic curves. Biometrics.

[74] Hanley JA, McNeil BJ (1982). The meaning and use of the area under a receiver operating characteristic (ROC) curve. Radiol..

[75] de Winter JCF (2013). Using the Student’s t-test with extremely small sample sizes. *Pract. Assess*. Res. Eval..

[76] Raghavachari N (2012). A systematic comparison and evaluation of high density exon arrays and RNA-seq technology used to unravel the peripheral blood transcriptome of sickle cell disease. BMC Med. Genomics.

[77] Zhao S, Fung-Leung WP, Bittner A, Ngo K, Liu X (2014). Comparison of RNA-seq and microarray in transcriptome profiling of activated T cells. PLOS One.

[78] Lê Cao KA, Rohart F, McHugh L, Korn O, Wells CA (2014). YuGene: a simple approach to scale gene expression data derived from different platforms for integrated analyses. Genomics.

[79] W. Zhang W (2015). Comparison of RNA-seq and microarray-based models for clinical endpoint prediction. Genome Biol..

[80] F. F. Millenaar FF (2006). How to decide? Different methods of calculating gene expression from short oligonucleotide array data will give different results. BMC Bioinformatics.

[81] Jiang N (2008). Methods for evaluating gene expression from Affymetrix microarray datasets. BMC Bioinformatics.

[82] Fonseca NA, Marioni J, Brazma A (2014). RNA-seq gene profiling - a systematic empirical comparison. PLOS One.

[83] Williams CR, Baccarella A, Parrish JZ, Kim CC (2016). Trimming of sequence reads alters RNA-seq gene expression estimates. BMC Bioinformatics.

[84] Xu, J. *et al*. Comprehensive assessments of RNA-seq by the SEQC Consortium: FDA-led efforts advance precision medicine. *Pharmaceutics* **8**, pii: E8, doi:10.3390/pharmaceutics8010008 (2016). 10.3390/pharmaceutics8010008 PMC4810084 26999190

[85] Macrae, B. & Nastouli, E. University College London Hospitals (UCHL) Virology User Manual version 16.0. Policy Unique Reference # 35-52429909. Authorization date 03-feb-2015. https://www.uclh.nhs.uk/OurServices/ServiceA-Z/PATH/PATHMICRO/VIRO/Documents/Virology_user_manual.pdf.

[86] Dillies MA (2013). A comprehensive evaluation of normalization methods for Illumina high-throughput RNA sequencing data analysis. Brief. Bioinform..

[87] Ojala M, Garriga GC (2010). Permutation Tests for Studying Classifier Performance. J. Mach. Learn. Res..

[88] Kuparinen T (2013). Cytomegalovirus (CMV)-dependent and -independent changes in the aging of the human immune system: a transcriptomic analysis. Exp. Gerontol..

[89] Kwissa M (2014). Dengue virus infection induces expansion of a CD14(+)CD16(+) monocyte population that stimulates plasmablast differentiation. Cell Host & Microbe.

[90] S. Malhotra S (2013). Transcriptional profiling of the circulating immune response to Lassa virus in an aerosol model of exposure. PLOS Negl. Trop. Dis..

[91] Nascimento EJM (2009). Gene expression profiling during early acute febrile stage of dengue infection can predict the disease outcome. PLOS ONE.

[92] Huang Y (2011). Temporal dynamics of host molecular responses differentiate symptomatic and asymptomatic influenza a infection. PLOS Genet..

[93] Bolen CR (2013). The blood transcriptional signature of chronic hepatitis C virus Is consistent with an ongoing interferon-mediated antiviral response. J. Interferon Cytokine Res..

[94] Djavani MM (2007). Early blood profiles of virus infection in a monkey model for Lassa fever. J. Virol..

[95] Ioannidis I (2012). Plasticity and virus specificity of the airway epithelial cell immune response during respiratory virus infection. J. Virol..

[96] Zilliox MJ, Moss WJ, Griffin DE (2007). Gene expression changes in peripheral blood mononuclear cells during measles virus infection. Clin. Vaccine Immunol..

[97] Wang Y (2007). Rotavirus infection alters peripheral T-cell homeostasis in children with acute diarrhea. J. Virol..

[98] Ahn SH (2013). Gene expression-based classifiers identify *Staphylococcus aureus* infection in mice and humans. PLOS ONE.

[99] Bloom CI (2013). Transcriptional blood signatures distinguish pulmonary tuberculosis, pulmonary sarcoidosis, pneumonias and lung cancers. PLOS ONE.

[100] Dickinson P (2015). Whole blood gene expression profiling of neonates with confirmed bacterial sepsis. Genom. Data.

[101] Banchereau R (2012). Host immune transcriptional profiles reflect the variability in clinical disease manifestations in patients with *Staphylococcus aureus* infections. PLoS ONE.

[102] Lee HM, Sugino H, Aoki C, Nishimoto N (2011). Underexpression of mitochondrial-DNA encoded ATP synthesis-related genes and DNA repair genes in systemic lupus erythematosus. Arthritis Res. Ther..

[103] Bjornsdottir US (2011). Pathways activated during human asthma exacerbation as revealed by gene expression patterns in blood. PLOS ONE.

[104] de Jong S (2012). A gene co-expression network in whole blood of schizophrenia patients is independent of antipsychotic-use and enriched for brain-expressed genes. PLOS ONE.

[105] Xiao W (2011). A genomic storm in critically injured humans. J. Exp. Med..

[106] Wingo AP, Gibson G (2015). Blood gene expression profiles suggest altered immune function associated with symptoms of generalized anxiety disorder. Brain Behav. Immun..

[107] Ardura MI (2009). Enhanced monocyte response and decreased central memory T cells in children with invasive *Staphylococcus aureus* infections. PLOS ONE.

[108] Preininger M (2013). Blood-informative transcripts define nine common axes of peripheral blood gene expression. PLOS Genet..

[109] Bogunovic D (2012). Mycobacterial disease and impaired IFN-γ immunity in humans with inherited ISG15 deficiency. Science.

[110] X. Zhang X (2015). Human intracellular ISG15 prevents interferon-α/β over-amplification and auto-inflammation. Nature.

[111] Okumura A, Lu G, Pitha-Rowe I, Pitha PM (2006). Innate antiviral response targets HIV-1 release by the induction of ubiquitin-like protein ISG15. Proc. Natl. Acad. Sci. USA..

[112] Okumura A, Pitha PM, Harty RN (2008). ISG15 inhibits Ebola VP40 VLP budding in an L-domain-dependent manner by blocking Nedd4 ligase activity. Proc. Natl. Acad. Sci. USA..

[113] Zhou P, Goldstein S, Devadas K, Tewari D, Notkins AL (1997). Human CD4+ cells transfected with IL-16 cDNA are resistant to HIV-1 infection: inhibition of mRNA expression. Nat. Med..

[114] Zhou P, Devadas K, Tewari D, Jegorow A, Notkins AL (1999). Processing, secretion, and anti-HIV-1 activity of IL-16 with or without a signal peptide in CD4+ T cells. J. Immunol..

[115] Zhu J (2014). Antiviral activity of human OASL protein is mediated by enhancing signaling of the RIG-I RNA sensor. Immunity.

[116] Alcorn JF, Sarkar SN (2014). What is the oligoadenylate synthetases-like protein and does it have therapeutic potential for influenza?. Expert Rev. Respir. Med..

[117] Gray JX (1996). CD97 is a processed, seven-transmembrane, heterodimeric receptor associated with inflammation. J. Immunol..

[118] Leemans JC (2004). The epidermal growth factor-seven transmembrane (EGF-TM7) receptor CD97 is required for neutrophil migration and host defense. J. Immunol..

[119] Qiu X (2015). Diversity in compartmental dynamics of gene regulatory networks: the immune response in primary influenza A infection in mice. PLOS One.

[120] Connor JH (2015). Transcriptional profiling of the immune response to Marburg virus infection. J. Virol..

[121] Lin KL (2015). Temporal characterization of Marburg virus Angola infection following aerosol challenge in rhesus macaques. J. Virol..

