# Supplementary material for: A Four-Biomarker Blood Signature Discriminates Systemic Inflammation Due to Viral Infection Versus Other Etiologies
Source: Sci Rep. 2017 Jun 6;7:2914. doi: 10.1038/s41598-017-02325-8 (PMC5460227; doi:10.1038/s41598-017-02325-8)
Supplement: Supplementary file 1 — Supplementary Material [file 41598_2017_2325_MOESM1_ESM.pdf]

Manuscript: A Four-Biomarker Blood Signature Discriminates Systemic Inflammation Due to Viral Infection Versus Other Etiologies

Authors: D.L. Sampson, B.A. Fox[1], T.D. Yager, S. Bhide, S. Cermelli, L.C. McHugh, T.A. Seldon, R.A. Brandon, E. Sullivan, J.J. Zimmerman, M. Noursadeghi, R.B. Brandon.

Item	Title
Supplementary Figure S1	Pan-viral signature scores for children with and without detectable human rhinovirus (Baltimore Group IV)
Supplementary Figure S2	Time-course of pan-viral signature in humans, after intra-nasal infection with H3N2 influenza A (Baltimore Group V)
Supplementary Figure S3	Time-course of pan-viral signature in mice after intra-nasal infection with H3N2 influenza A (Baltimore Group V)
Supplementary Figure S4	Pan-viral signature score over time, for liver biopsy tissue from chimpanzees inoculated with Hepatitis C Virus or Hepatitis E Virus (Baltimore Group IV)
Supplementary Figure S5	Time-course of the pan-viral signature, for macaque monkeys infected with Marburg virus (Baltimore Group V)
Supplementary Figure S6	Consort diagram for the FEVER study
Supplementary Figure S7	Examples of applying the 1,000 tree Random Forest model to the GSE63990 dataset with randomly shuffled group labels
Supplementary Figure S8	A plot of the distribution of null results from a permutation test applied to the GSE63990 dataset
Supplementary Text S1	Description of the FEVER study
Supplementary Text S2	Description of the GAPPSS study
Supplementary Table S1	Summary of clinical data for patients from the FEVER study
Supplementary Table S2	FEVER study line data (gene expression values from RNA-seq)

Item	Title
Supplementary Table S3	Characteristics of the GAPPSS Cohort
Supplementary Table S4	GAPPSS study line data (gene expression values from RNA-seq)
Supplementary Table S5	Line data from publication of Tsalik et al. (2016), used in Random Forest analysis
Supplementary Table S6	Identities of probes used in analysis of GEO datasets
Supplementary Animation S1	Resolution of patients with acute respiratory illness (ARI) into three clusters corresponding to bacterial infection, viral infection, and non-infectious illness (GSE63990).

Supplementary Figure S1 | Pan-viral signature scores for children with and without detectable human rhinovirus (Baltimore Group IV)

Human rhinoviruses (HRV, Baltimore Group IV, positive-sense single-stranded RNA) are the most frequent etiological agents of respiratory tract infections in both adults and children (Heinonen et al., 2016). Children infected with HRV display a broad spectrum of clinical signs, and questions have been raised over the etiological role of HRV in respiratory disease.

Dataset GSE67059 was generated in a study (Heinonen *et al.*, 2016) that identified a whole blood gene expression signature for differentiating pediatric subjects with or without detectable human rhinovirus (HRV). The study included respiratory symptomatic and asymptomatic subjects as either inpatients or outpatients. Asymptomatic subjects included healthy children, and also those presenting for minor surgery that did not involve the respiratory system. GSE67059 comprised one of our validation datasets, and was *not* used in discovery of the pan-viral signature.

We observed differences in the pan-viral signature when comparing subjects that were HRV(-) to subjects that were HRV(+), irrespective of whether the infection-positive subjects had symptoms or were inpatients or outpatients (**Supplementary Figure S1**).

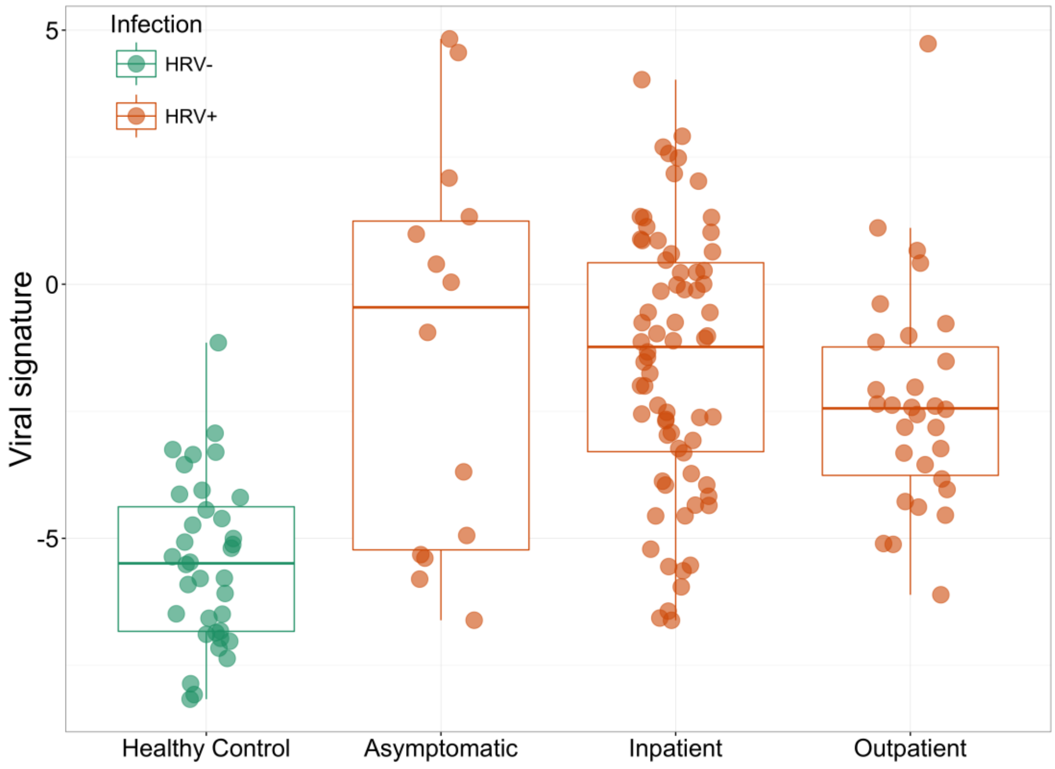

Supplementary Figure S1 | Pan-viral signature scores for children with and without detectable human rhinovirus (Baltimore Group IV). The pan-viral signature AUCs were: 0.905 for healthy (HRV-) versus all HRV+ subjects, 0.896 for healthy versus HRV+ inpatients, 0.894 for healthy versus HRV+ outpatients, and 0.806 for healthy versus HRV+ asymptomatic subjects. In this dataset, the pan-viral signature appears therefore not related to severity of respiratory symptoms, but rather to the presence of HRV.

<u>Reference</u>

Heinonen, S. *et al*. Rhinovirus Detection in Symptomatic and Asymptomatic Children: Value of Host Transcriptome Analysis. *Am. J. Respir. Crit. Care Med.* **193**, 772–782 (2016).

Supplementary Figure S2 | Time-course of pan-viral signature in humans, after intra-nasal infection with H3N2 influenza A (Baltimore Group V)

Dataset GSE30550 (Huang *et al.*, 2011) was used in the discovery of the pan-viral signature. The following samples from GSE30550 were used in the discovery process: infection-negative samples taken pre-inoculation (0 hours); and infection-positive samples taken at times corresponding to peak symptom presentation (21-69 hours). **Supplementary Figure S2** presents a box-and-whisker plot of the the pan-viral signature score for symptomatic human subjects, sampled over a 108 hour period following intra-nasal inoculation of influenza H3N2. A significant increase in pan-viral signature score relative to its pre-inoculation value can be seen as early as 36 hours, and reaches a maximum at 69 hours.

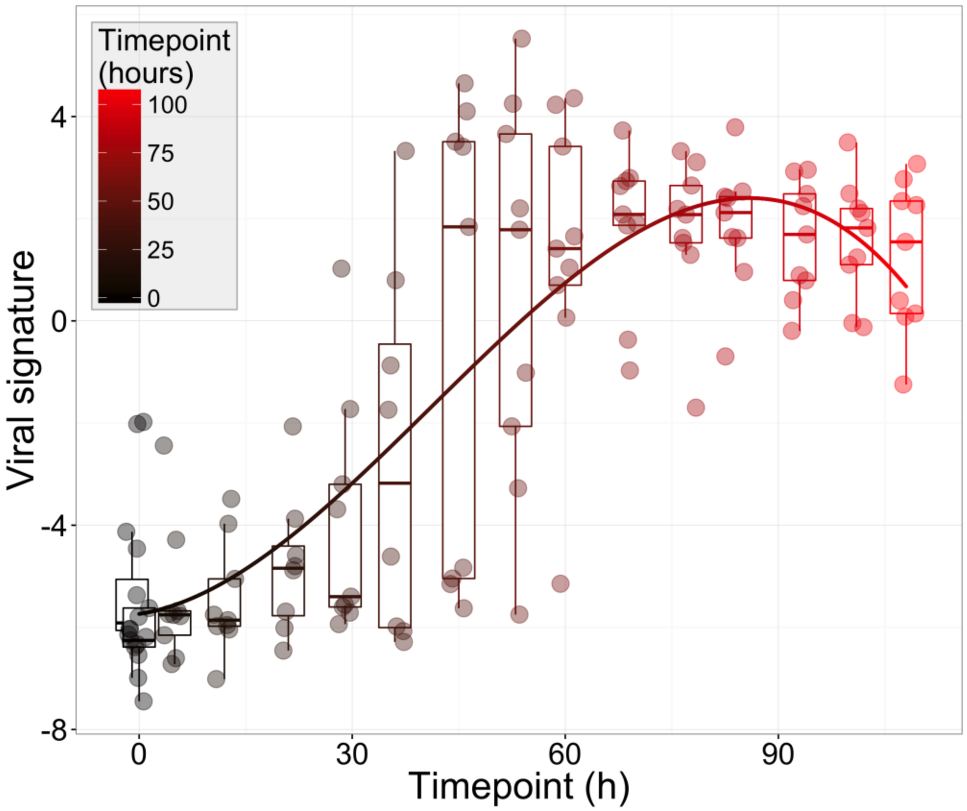

Supplementary Figure S2 | Time-course of pan-viral signature in humans, after intra-nasal infection with H3N2 influenza A (Baltimore Group V). Box-and-whisker plot of the pan-viral signature score for symptomatic human subjects, based on sampling over a 108-hour period following intra-nasal inoculation of influenza H3N2. The time-course was visualized by fitting the observed points to a third-order polynomial.

A parallel time-course for earliest detection and peak score in mice (**Supplementary Figure S3**) in response to experimental inoculation with influenza H3N2 suggests that this virus produces a similar pan-viral signature response in the two species.

<u>Reference</u>

Huang, Y. *et al.* Temporal Dynamics of Host Molecular Responses Differentiate Symptomatic and Asymptomatic Influenza A Infection. *PLOS Genet.* **7**, e1002234–17 (2011).

Supplementary Figure S3 | Time-course of pan-viral signature in mice after intra-nasal infection with H3N2 influenza A (Baltimore Group V)

GSE57384 is a validation dataset that was _not_ used in discovery of the pan-viral signature. In the study represented by GSE57384 (Qiu *et al.*, 2015), mice were intra-nasally inoculated with influenza A virus (H3N2) and followed over time. Three mice were sacrificed at 9 days prior to inoculation as a control, and then daily from day 0 to day 10 post-inoculation. As shown in **Supplementary Figure S3**, the pan-viral signature first showed a consistent increase on day 2, and peaked on day 3. It then decreased to pre-inoculation levels by day 9. A similar response curve was observed in experimental human inoculation with the same virus strain (**Supplementary Figure S2**). Note: OASL1 is the mouse ortholog of the human OASL gene (Elkhateeb *et al.*, 2016) and was used here.

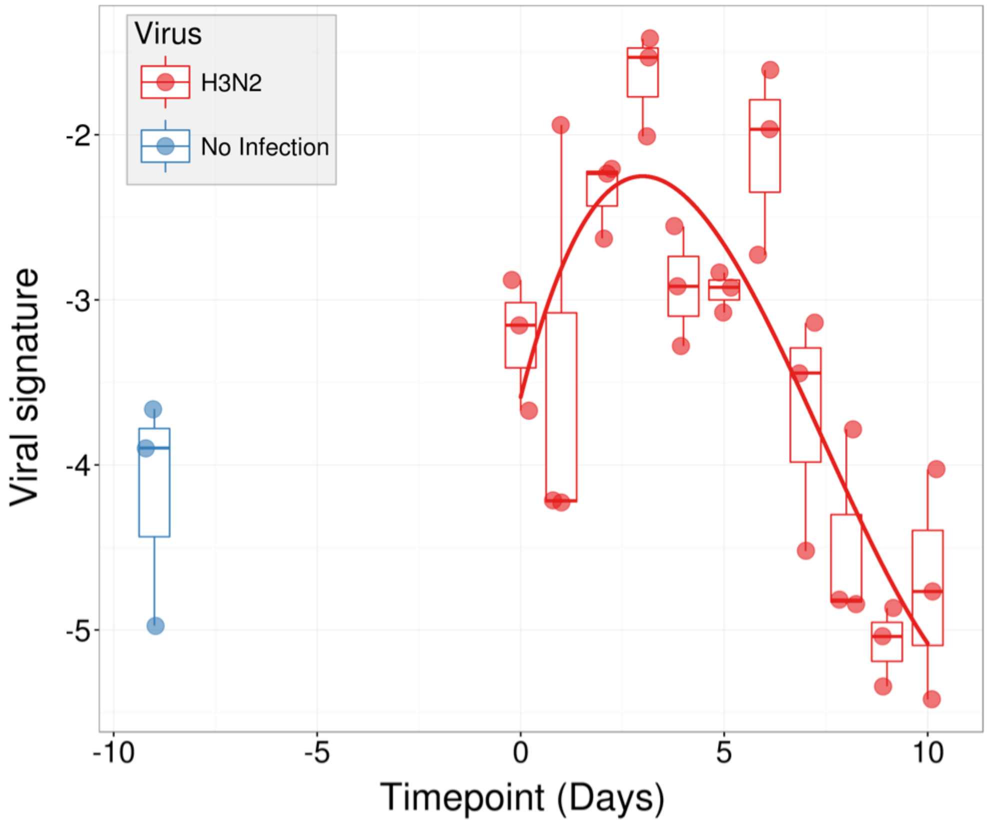

Supplementary Figure S3 | Time-course of pan-viral signature in mice after intra-nasal infection with H3N2 influenza A (Baltimore Group V). Mice were intra-nasally inoculated with influenza A virus (H3N2). Three mice were sacrificed at 9 days prior to inoculation as a control, and then daily from day 0 to day 10 post-inoculation. The time-course was visualized by fitting the observed points to a third-order polynomial. OASL1 is the mouse ortholog of the human OASL gene (Elkhateeb *et al.*, 2016) and was used in this plot.

<u>References</u>

Qiu, X. *et al.* Diversity in Compartmental Dynamics of Gene Regulatory Networks: The Immune Response in Primary Influenza A Infection in Mice. *PLoS One* **10**, e0138110 (2015).

Elkhateeb, E. *et al.* The role of mouse 2',5'-oligoadenylate synthetase 1 paralogs. Infect. Genet. Evol. 45, 393-401 (2016).

Supplementary Figure S4 | Pan-viral signature score over time, for liver biopsy tissue from chimpanzees inoculated with Hepatitis C Virus or Hepatitis E Virus (Baltimore Group IV)

To date, the chimpanzee is the only animal model for Hepatitis C (HCV) and Hepatitis E (HEV) infection in humans. Dataset GSE22160, used in validation of the pan-viral signature, was generated in a study of the temporal gene expression responses in liver biopsies from chimpanzees inoculated with either HCV or HEV (Yu *et al.*, 2014). **Supplementary Figure S4** presents the pan-viral signature in the chimpanzee liver biopsy samples, as a function of time after inoculation with HCV (**Panel A**) or HEV (**Panel B**). The pan-viral signature score increased roughly in proportion to the extent of viremia detected in plasma using virus-specific RT-PCR assays (Figure 1 in Yu *et al.*, 2014) the peak of which preceded both the antibody response and peak liver histological activity index by 1 to 4 weeks for both viruses. The gene expression responses reported by Yu *et al.* were different for each virus, but in both cases included many interferon-induced genes. For the pan-viral signature, the AUC values were: 1.00 for weeks 1, 3 or 4 vs. week 0; and 0.96 for week 2 vs. week 0.

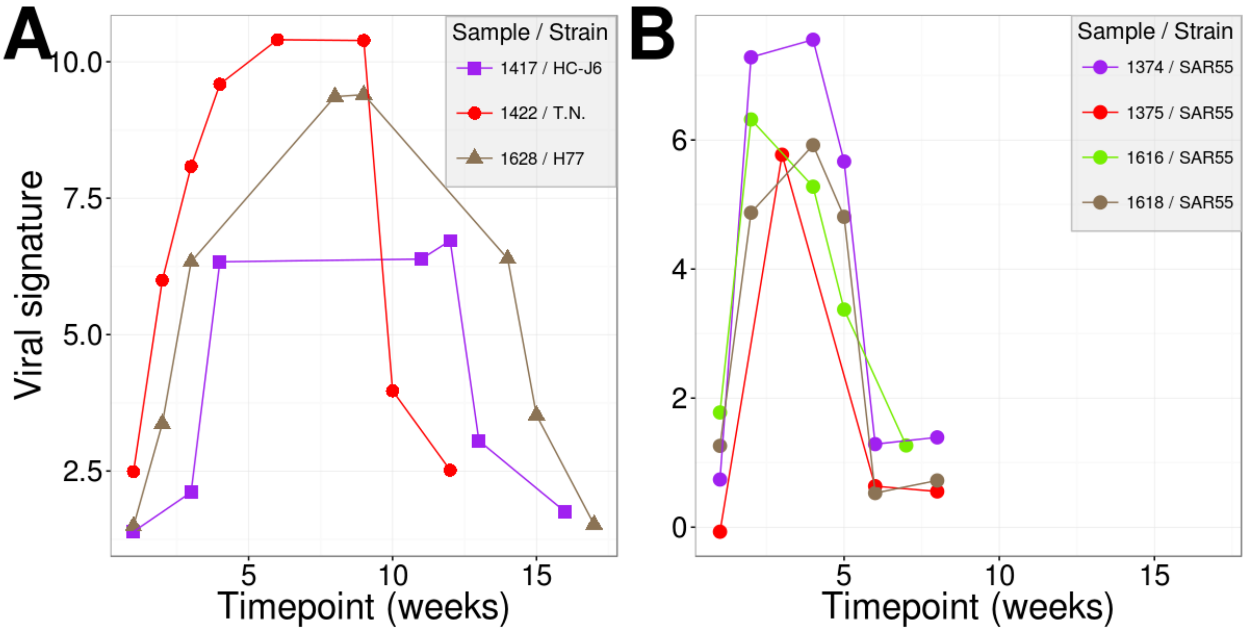

Supplementary Figure S4 | Pan-viral signature score over time, for liver biopsy tissue from chimpanzees inoculated with Hepatitis C Virus or Hepatitis E Virus (Baltimore Group IV). Panel (A): Pan-viral signature in the chimpanzee liver biopsy samples, after inoculation with HCV (n=3). Panel (B): Pan-viral signature in the chimpanzee liver biopsy samples, after inoculation with HEV (n=4). For the pan-viral signature, the AUC values were: 1.00 for weeks 1, 3 or 4 vs. week 0; and 0.96 for week 2 vs. week 0.

<u>Reference</u>

Yu, C. *et al.* Pathogenesis of Hepatitis E Virus and Hepatitis C Virus in Chimpanzees: Similarities and Differences. *J. Virol.* **84**, 11264–11278 (2010).

Supplementary Figure S5 | Time-course of the pan-viral signature, for macaque monkeys infected with Marburg virus (Baltimore Group V)

Validation dataset GSE58287 was derived from studies (Conner et al., 2015; Lin et al., 2015) in which 15 cynomolgus macaque monkeys (*Macaca fascicularis*) were infected with Marburg virus (Baltimore Group V, negative-sense single-stranded RNA; 1,000 pfu/inoculation) as a model for Ebola virus infection in humans. The macaques were followed over a nine-day period, with three animals sacrificed at each two-day interval. Results are shown in **Supplementary Figure S5**. The first major elevation of the pan-viral signature value was observed on Day 3 post-exposure, which correlated with the first detection of viral antigen in regional lymph nodes, but preceded the first detectable viremia (Day 4) or elevated body temperature (Day 5) as detailed in Table 3 and Figure 1 of Lin *et al.* (2015). The peak cytokine and blood gene expression values reported in Lin *et al.* (2015) coincided with a peak in the value of the pan-viral signature on day 7. The AUC obtained for the pan-viral signature when comparing pre- and post-infection samples was 0.98.

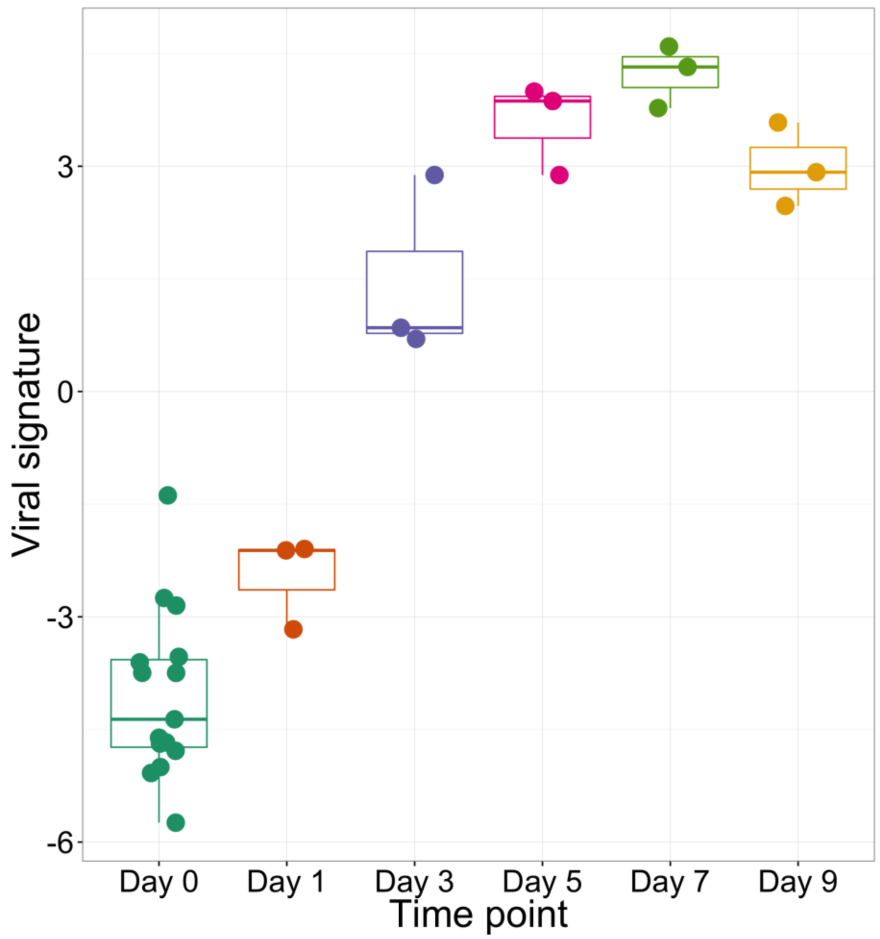

Supplementary Figure S5 | Time-course of the pan-viral signature, for macaque monkeys infected with Marburg virus (Baltimore Group V). Fifteen cynomolgus macaque monkeys (*Macaca fascicularis*) were infected with Marburg virus (Baltimore Group V, negative-sense single-stranded RNA; 1,000 pfu/inoculation). The macaques were followed over a nine-day period, with three animals sacrificed at each two-day interval. The AUC obtained for the pan-viral signature when comparing pre- and post-infection samples was 0.98.

References

Connor, J.H. *et al.* Transcriptional Profiling of the Immune Response to Marburg Virus Infection. *J. Virol.* **89**, 9865–9874 (2015).

Lin, K.L. *et al.* Temporal Characterization of Marburg Virus Angola Infection following Aerosol Challenge in Rhesus Macaques. *J. Virol.* **89**, 9875–9885 (2015).

Supplementary Figure S6 | Consort diagram for the FEVER study

See **Supplementary Text 1** for further details.

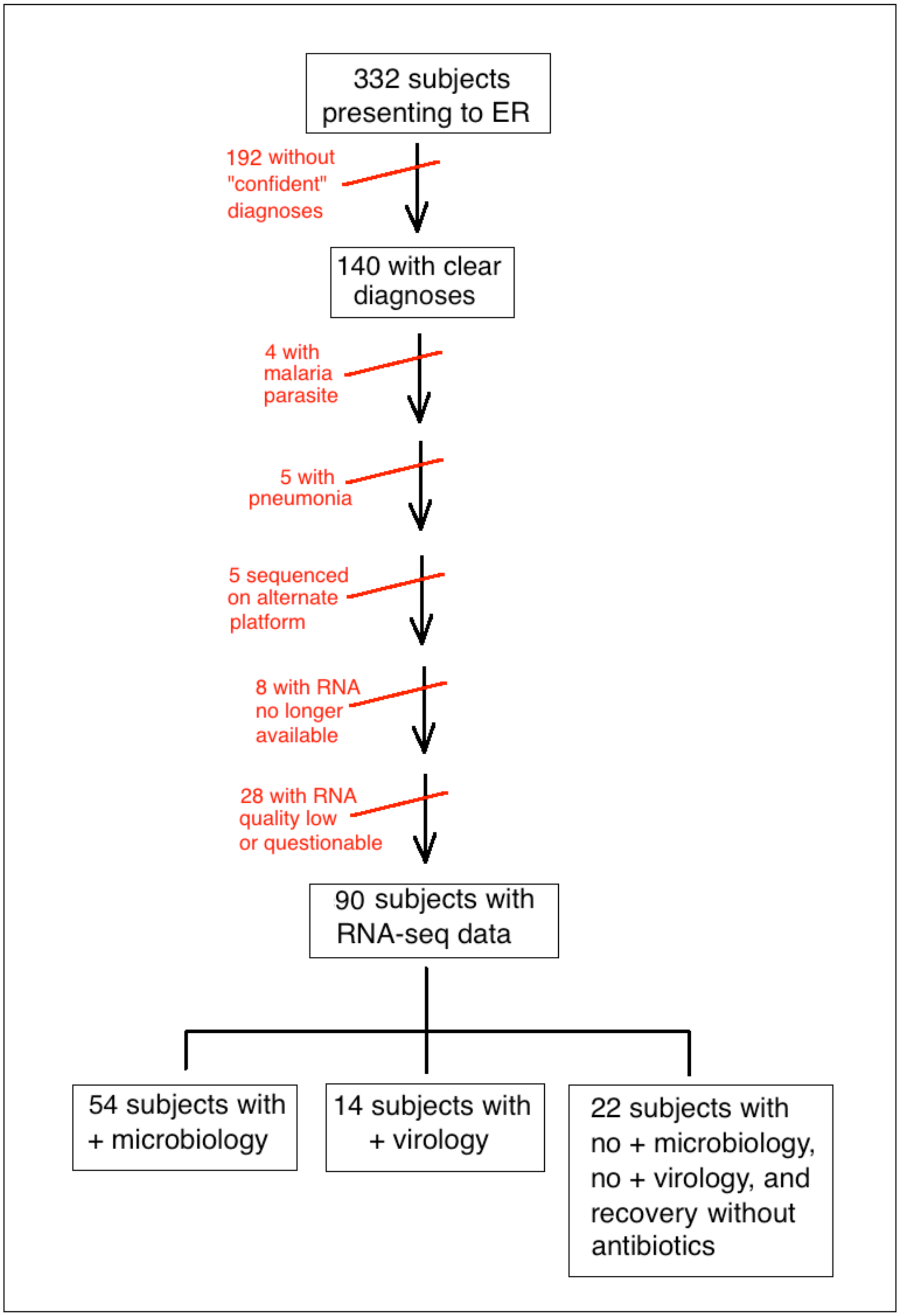

Supplementary Figure S7 | Examples of applying the 1,000 tree Random Forest model to the GSE63990 dataset with randomly shuffled group labels

We conducted 2,000 iterations of a permutation analysis of dataset GSE63990. Each permutation iteration consisted of randomly shuffling the dataset's group/response labels, before inputting the data into a Random Forest model (see Methods section and Figure 8 in the manuscript). Each panel in Supplementary Figure S7 shows a 2-dimensional projection of a randomly selected permutation of the GFE63990 dataset. As expected, each of the permutations shows no significant group separation.

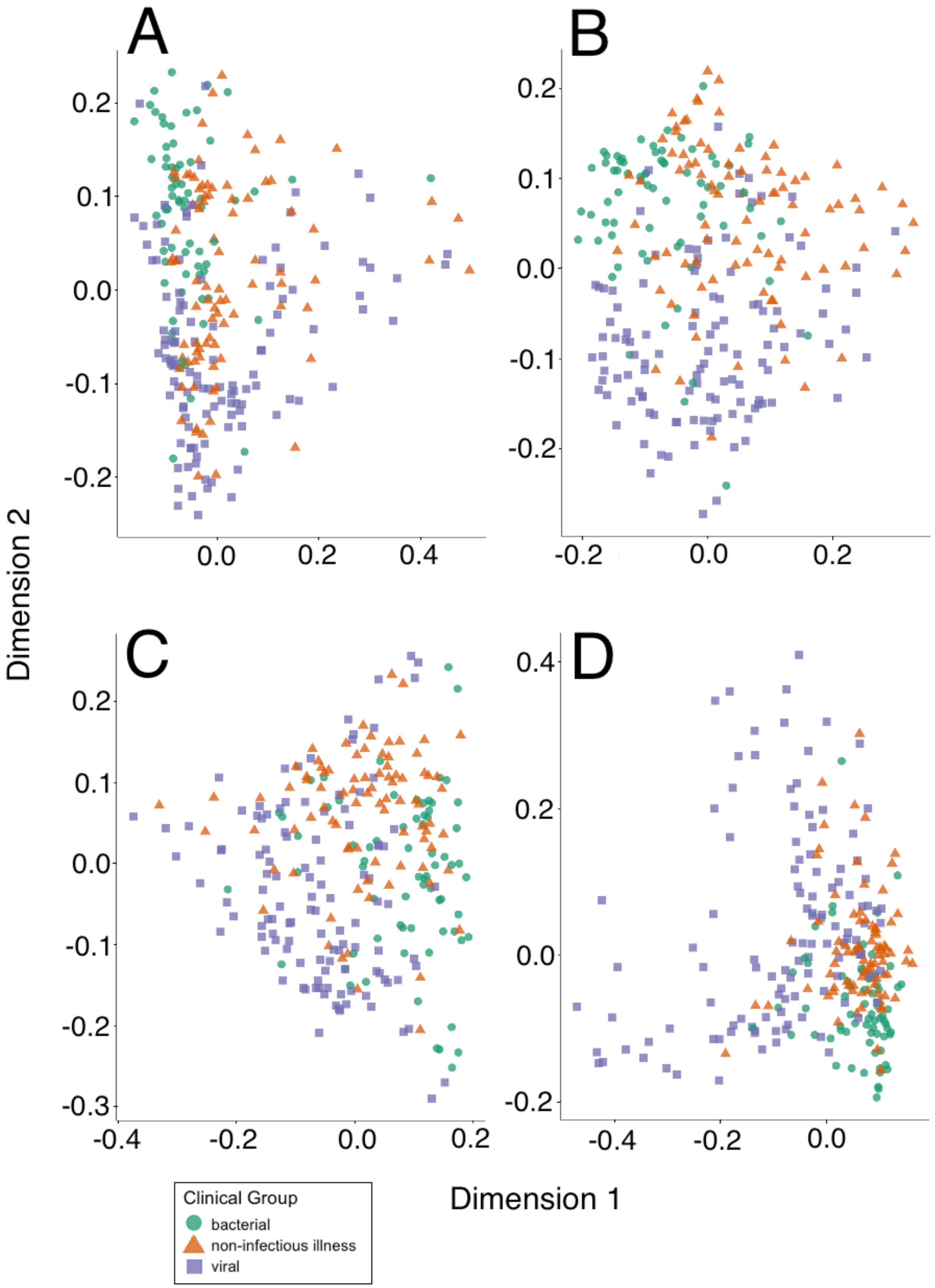

A
B
C
D
Dimension 2
Dimension 1
Clinical Group
bacterial
non-infectious illness
viral

Supplementary Figure S8 | A plot of the distribution of null results from a permutation test applied to the GSE63990 dataset

Statistical H_0 distribution of the logloss function produced via permutation analysis. Following the method described in Li et al. (2010), the group labels in GSE63990 were randomly shuffled in each of 2,000 permutation iterations. Each permutation iteration was then reanalyzed using a Random Forest model, as described in Supplementary Figure S7 and the Methods section of the manuscript. The solid red vertical bar indicates placement of the logloss function for the un-shuffled analysis of the original GSE63990 dataset.

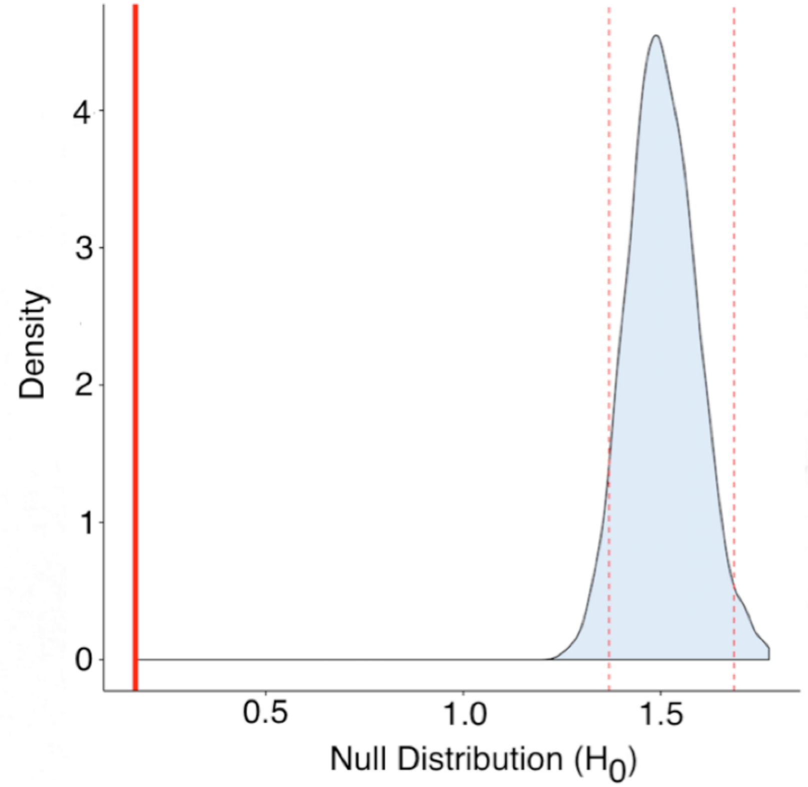

Reference

Li, J. *et al.* Identification of high-quality cancer prognostic markers and metastasis network modules. *Nat. Commun.* **1**, 34 (2010).

Supplementary Text S1 | Description of the FEVER study

Study Identifiers:

- Title: Expression profiling in patients presenting to hospital with fever.
- Sponsorship ID: University College London sponsorship Ref No: 09/H701/103.
- Principal Investigator: Mahdad Noursadeghi, University College London, WC1E 6BT, United Kingdom.

Study Site:

- Accident and Emergency Department, University College London Hospital, 235 Euston Road, London, NW1 2BU

Ethics:

- UK National Research Ethics services (reference number: 09/H0701/103)
- All participants provided written informed consent.

Inclusion/Exclusion Criteria:

- Inclusion criteria: Admitted to hospital, >16 years of age, fever (>37.5C), suspicion of infection.
- Exclusion criteria: Known immunodeficiency, immunomodulatory therapy (e.g. steroids, chemotherapy, IFN), or immunization within 2 weeks prior to presentation.

Demographics:

- Final cohort size N=90 (43 male, 47 female).
- 50 Caucasian, 13 Asian British, 10 Black British, 17 other or not stated.
- Age range 17-91 years (mean 48.7 years).
- A consort diagram for the FEVER study is given in **Supplementary Figure S6**.

Clinical Workup:

- Specimens collected: blood, stool, urine, blood film, throat swab for clinical microbiology. All patients had blood culture performed. Virology testing was only performed on those patients suspected of a viral infection, and involved use of one or more single-virus diagnostic tests (e.g. PCR for influenza, serology for dengue, etc.) based on the attending clinician's judgment.
- Virology testing was performed at the University College London Hospitals (UCHL) Clinical Virology Laboratory, as described in UCHLVirology User Manual version 16.0 (Macrae & Nastouli, 2015; reference # 122 in manuscript).
- Confirmed viral infections: Baltimore Group I (Herpeviridae: Varicella-Zoster, Epstein-Barr, Cytomegalovirus); Baltimore Group IV (Flaviviridae: Dengue virus); Baltimore Group V (Orthomyxoviridae: Influenza A, Influenza B)
- See **Supplementary Table S1** for summary of clinical data.

Diagnosis:

- Information considered: clinical data gained at time of presentation; clinical outcomes at 3 months; microbiology results; molecular virology test results.

Reference:

Macrae, B. & Nastouli, E. University College London Hospitals (UCHL) Virology User Manual version 16.0. Policy Unique Reference # 35-52429909. Authorization date 03-feb-2015. Accessed at the following website: https://www.uclh.nhs.uk/OurServices/ServiceA-Z/PATH/PATHMICRO/VIRO/Documents/Virology_user_manual.pdf]

Supplementary Text S2 | Description of the GAPPSS study

Study Identifiers:

- Title: Genotypes and Phenotypes in Pediatric SIRS and Sepsis (GAPPSS).
- ClinicalTrials.gov identifier: NCT02728401.
- Principal Investigator: Dr. Jerry J. Zimmerman, Seattle Children's Hospital, Seattle WA.

Study Sites:

- Pediatric Intensive Care Unit (PICU), Seattle Children's Hospital, Seattle WA.
- Cardiac Intensive Care Unit (CICU), Seattle Children's Hospital, Seattle WA.

Ethics:

- Ethics approval gained through the hospital IRB (docket #14761).
- Signed consent from English-speaking parents.

Inclusion/Exclusion Criteria:

Control Group

- Inclusion criteria: Admitted to intensive care, 1-18 years of age, at least 2 SIRS criteria, in-dwelling vascular catheter, not previously enrolled in the study, > 10kg, open heart surgery requiring bypass.
- Exclusion criteria: Not expected to survive, pre- or post-operative positive microbiology, malignancy or immune disorder, corticosteroid use (pre-, post- or intra-operative).

Sepsis Group

- Inclusion criteria: Admitted to intensive care, 38 weeks Estimated Gestational Age -18 years of age, at least 2 SIRS criteria and one organ dysfunction, in-dwelling vascular catheter, not previously enrolled in the study, strongly suspected or documented bacterial infection, > 4kg, severe respiratory dysfunction requiring invasive or non-invasive positive pressure mechanical ventilation.
- Exclusion criteria: Not expected to survive, PICU nosocomial infection, ward of the state, corticosteroid use (pre-, post- or intra-operative).

Viral Group

- Inclusion criteria: Admitted to intensive care, 38 weeks Estimated Gestational Age -18 years of age, in-dwelling vascular catheter, not previously enrolled in the study, Positive PCR verifying a viral infection, > 4kg, severe respiratory dysfunction requiring invasive or non-invasive positive pressure mechanical ventilation.
- Exclusion criteria: Not expected to survive, PICU nosocomial infection, ward of the state, corticosteroid use (pre-, post- or intra-operative).

Demographics:

- 69 children in intensive care diagnosed with sterile systemic inflammation (post-cardiac surgery, n=29, 12 female), sepsis (n=25, 12 female), sepsis with an identified viral coinfection (n=10, 5 female) or viral-associated systemic inflammation (n=5, 3 female). Overall gender ratio 37 males / 32 females.

Clinical Workup:

- Specimens collected: blood and nasal swab for clinical microbiology and virology.

- Nasal swabs were tested using a FilmArray Respiratory Panel (BioFire Diagnostics, Salt Lake City UT). Viruses and bacteria tested for on this panel included: Adenovirus, Coronavirus 229E, Coronavirus HKU1, Coronavirus NL63, Coronavirus OC43, Human Metapneumovirus, Human Rhinovirus/Enterovirus, Influenza A, Influenza A subtype H1, Influenza A subtype 2009 H1, Influenza A subtype H3, Influenza B, Parainfluenza virus 1, Parainfluenza virus 2, Parainfluenza virus 3, Parainfluenza virus 4, Respiratory syncytial virus, *B. pertussis*, *C. pneumoniae*, and *M. pneumoniae.*

Diagnosis:

- Information considered: Diagnosis based on clinical data gained at time of presentation and all clinical diagnostic and microbiology results.
- Final diagnosis: patients were diagnosed retrospectively using all available information, and were classified into the following categories: post-surgical systemic inflammation (Control; n=29); sepsis (n=25); sepsis with a virus identified (n=10), and viral-associated systemic inflammation (n=5).

Supplementary Table S1 | Summary of clinical data for patients from the FEVER study

The entire cohort consisted of 54 patients with confirmed bacterial infection, 14 patients with confirmed viral infection, and 22 patients of indeterminate status.

Covariate	Clinical group	N	Mean	Median	SD	Range
Temperature (°C)	Bacterial	54	38.7	38.7	0.8	37.5 - 40.6
	Viral	14	38.8	38.5	0.8	37.7 - 40.1
	Indeterminate	21	38.3	38.1	0.5	37.5 - 39.4
C-reactive protein (mg/L)	Bacterial	47	106	67.9	91.9	3.9 - 355
	Viral	12	37.9	17.1	43.1	3.9 - 146.7
	Indeterminate	20	67.6	50.6	74.5	0.6 - 282.5
White cell count (10^6 cells/mL)	Bacterial	47	13.9	13.7	5.2	3.3 - 27.1
	Viral	12	8.6	8.0	3.8	3.7 - 15.0
	Indeterminate	20	10.1	10.8	4.2	0.8 - 17.6
Neutrophil count (10^6 cells/mL)	Bacterial	47	83.0	87.7	15.0	5.2 - 98.0
	Viral	12	68.6	75.6	16.3	41.4 - 88.0
	Indeterminate	20	79.1	78.7	9.6	50.9 - 94.1
Lymphocyte count (10^6 cells/mL)	Bacterial	47	8.7	5.5	8.1	1.1 - 38.7
	Viral	12	18.7	13.8	12.5	5.7 - 42.8
	Indeterminate	20	11.8	11.5	7.1	1.9 - 32.8
Monocyte count (10^6 cells/mL)	Bacterial	47	6.7	6.5	3.8	0.5 - 23.6
	Viral	12	11.01	8.90	5.44	4.3 - 23.7
	Indeterminate	20	7.7	7.0	4.1	1.4 - 17.3
Eosinophil count (10^6 cells/mL)	Bacterial	47	0.6	0.1	2.4	0 - 15.6
	Viral	12	0.3	0.1	0.4	0 - 1.0
	Indeterminate	20	0.5	0.1	1.3	0 - 5.6
LOS ≤ 30 days*	Bacterial	43	7	6	5.4	1 - 26
	Viral	9	4	3	4.9	1-17
	Indeterminate	19	3.8	2	3.8	1-14

* Three bacterially-infected patients and two virally-infected patients had hospital length of stay (LOS) >30 days, and are not included in this tabulation of results by virtue of being outliers

Supplementary Table S2 | FEVER study line data (gene expression values from RNA-seq)

Key	Subject	Class	IL16	OASL	ISG15	ADGRE5	Viral Signature
1	PN031	Bacterial	67.45	25.51	28.89	229.08	-242.13
2	PN071	Bacterial	63.16	46.51	122.43	527.5	-421.72
3	PN091	Bacterial	57.07	7.44	20.15	434.31	-463.79
4	PN094	Bacterial	26.87	17.82	28.67	388.54	-368.92
5	PN099	Bacterial	54.94	8.41	30.21	208.41	-224.73
6	PN112	Bacterial	51.38	13.53	54.59	301.25	-284.51
7	PN114	Bacterial	65.02	21.37	9.47	260.47	-294.65
8	PN122	Bacterial	60.43	10.09	11.44	373	-411.9
9	PN126	Bacterial	70.44	21.42	10.07	352.48	-391.43
10	PN138	Bacterial	55.21	4.33	8.62	528.21	-570.47
11	PN139	Bacterial	90.37	7.05	12.1	156.04	-227.26
12	PN141	Bacterial	102.94	22.82	50.7	328.33	-357.75
13	PN146	Bacterial	55.07	15.16	32.24	438.63	-446.3
14	PN150	Bacterial	72.24	8.36	8.79	458.21	-513.3
15	PN156	Bacterial	69.51	33.68	71.14	500.61	-465.3
16	PN157	Bacterial	111.69	6.61	4.82	430.46	-530.72
17	PN162	Bacterial	87.93	22.1	31.73	425	-459.1
18	PN164	Bacterial	74.18	24.31	43.56	475.11	-481.42
19	PN166	Bacterial	103.69	36.74	321.08	348.08	-93.95
20	PN169	Bacterial	62.49	206.33	1342.66	249.35	1237.15
21	PN176	Bacterial	57.88	12.83	13.47	383.7	-415.28
22	PN177	Bacterial	61.04	15.79	50.78	456.74	-451.21
23	PN183	Bacterial	58.83	38.65	134.96	675.51	-560.73
24	PN184	Bacterial	63.49	21.82	65.13	188.07	-164.61
25	PN185	Bacterial	60.38	10.86	33.06	417.39	-433.85
26	PN190	Bacterial	38.29	73.66	143.92	495.36	-316.07
27	PN191	Bacterial	78.81	14.93	6.34	368.15	-425.69
28	PN195	Bacterial	70.78	56.72	75.31	177.57	-116.32
29	PN196	Bacterial	97.15	12.15	24.72	365.3	-425.58
30	PN200	Bacterial	56.63	12.97	22.22	230.67	-252.11
31	PN201	Bacterial	76.97	6.63	25.99	301.09	-345.44
32	PN202	Bacterial	73.41	16.56	80.27	338.76	-315.34
33	PN209	Bacterial	87.04	47.35	177.94	649.84	-511.59
34	PN212	Bacterial	51.12	10.07	14.06	229.46	-256.45
35	PN218	Bacterial	64.43	13.54	33.16	359.36	-377.09

Key	Subject	Class	IL16	OASL	ISG15	ADGRE5	Viral Signature
36	PN220	Bacterial	53.06	35.66	77.49	463.45	-403.36
37	PN221	Bacterial	74.82	55.5	369.68	509.58	-159.22
38	PN237	Bacterial	101.67	23.15	27.97	315.24	-365.79
39	PN239	Bacterial	61.29	40.14	125.26	314.97	-210.86
40	PN253	Bacterial	44.65	7.78	23.42	320.94	-334.39
41	PN255	Bacterial	78.2	8.35	8.52	311.5	-372.83
42	PN257	Bacterial	96.37	130.47	287.35	246.04	75.41
43	PN267	Bacterial	63.33	16.26	21.34	257.65	-283.38
44	PN272	Bacterial	55.37	5.56	15.4	326.38	-360.79
45	PN276	Bacterial	63.6	7.29	9.67	451.37	-498.01
46	PN288	Bacterial	76.81	12.92	15.9	501.04	-549.03
47	PN299	Bacterial	95.67	17.16	12.16	404.48	-470.83
48	PN305	Bacterial	67.11	11.58	16.23	354.81	-394.11
49	PN308	Bacterial	44.32	4.29	9.65	489.9	-520.28
50	PN309	Bacterial	71.42	3.95	12.98	447.69	-502.18
51	PN312	Bacterial	56.86	3.08	17.12	277.39	-314.05
52	PN317	Bacterial	47.67	32.52	124.18	563.47	-454.44
53	PN320	Bacterial	46.36	4.07	8.13	366.79	-400.95
54	PN335	Bacterial	49.63	89.55	308.83	351.21	-2.46
55	PN069	Indeterminate	69.89	229.8	1555.9	442.15	1273.66
56	PN070	Indeterminate	89.84	139.98	584.27	332.04	302.37
57	PN081	Indeterminate	67.85	9.04	8.05	459.49	-510.25
58	PN105	Indeterminate	107.44	16.8	18.4	569.69	-641.93
59	PN120	Indeterminate	112.42	9.57	10.38	213.26	-305.73
60	PN121	Indeterminate	77.26	53.6	146.81	472.26	-349.11
61	PN128	Indeterminate	52.73	20.64	21.05	215.71	-226.75
62	PN130	Indeterminate	92.05	15.6	14.08	543.63	-606
63	PN159	Indeterminate	105.67	82.27	168.79	235.8	-90.41
64	PN161	Indeterminate	78.6	20.96	39.52	330.69	-348.81
65	PN171	Indeterminate	77.84	49.91	84.19	327.83	-271.57
66	PN175	Indeterminate	49.45	55.41	128.85	1178.33	-1043.52
67	PN187	Indeterminate	83.6	59.07	241.46	259.07	-42.14
68	PN189.2	Indeterminate	78.26	23.39	53.94	244.25	-245.18
69	PN224	Indeterminate	71.59	3.83	6.86	413.17	-474.07
70	PN242	Indeterminate	114.46	8.88	16.33	350.27	-439.52
71	PN252	Indeterminate	53.19	536.25	3490.23	367.96	3605.33
72	PN260	Indeterminate	103.75	25.49	26.38	348.9	-400.78
73	PN285	Indeterminate	78.61	13.69	31.9	327.53	-360.55
74	PN298	Indeterminate	83.47	32.73	52.38	550.95	-549.31

Key	Subject	Class	IL16	OASL	ISG15	ADGRE5	Viral Signature
75	PN302	Indeterminate	96.05	96.21	378.21	440.75	-62.38
76	PN341	Indeterminate	49.47	118.8	448.32	502.82	14.83
77	PN074	Viral	61.58	130.67	851.08	294.47	625.7
78	PN088	Viral	89.98	170.59	485.99	370.6	196
79	PN098	Viral	48.15	422.25	2230.32	351.31	2253.11
80	PN102	Viral	97.5	290.38	2137.46	415.26	1915.08
81	PN193	Viral	57.35	508.05	3702.62	421.86	3731.46
82	PN223	Viral	64.32	319.43	2137.52	468.17	1924.46
83	PN227	Viral	66.89	211.04	949.42	407.91	685.66
84	PN228	Viral	63.97	78.08	121.92	196.27	-60.24
85	PN235	Viral	50.13	392.53	2580.46	508.94	2413.92
86	PN243	Viral	94.06	11.78	15.93	352.31	-418.66
87	PN247	Viral	62.68	75.11	315.32	337.63	-9.88
88	PN262	Viral	72.32	178.16	547.23	265.57	387.5
89	PN287	Viral	54.51	47.14	131.56	326.75	-202.56
90	PN319	Viral	69.11	355.08	2126.43	295.75	2116.65

Supplementary Table S3 | Characteristics of the GAPPSS Cohort

The calculations were derived from the supplemental material provided in Zimmerman *et al.* (2016).

**Variable**	**Sepsis (N=25)**	**Sepsis + Viral (N=10)**	**SIRS (N=29)**	**Viral (N=5)**
**Age** (years)<br>Median<br>Interquartile Range<br>Range	 14.1<br>6.5-15.2<br>0.4-17.5	 1.0<br>0.4-3.6<br>0.1-16.0	 5.3<br>3.1 – 13.4<br>0.5 – 16.6	 14.4<br>6.8 – 15.2<br>6.6 – 16.6
**Gender** (n, %)<br>Female	 12 (48.0%)	 5 (50.0%)	 12 (41.4%)	 3 (60.0%)
**PRISM III** (Admission Score)	7.6 ± 5.8	11.2 ± 7.5	7.0 ± 4.6	2.6 ± 1.7
**PELOD Score** (Day 1)	4.8 ± 2.5	4.9 ± 3.7	5.1 ± 2.2	3.0 ± 2.0
**Immune Competent** (N, % immune competent)	19 (76.0%)	3 (30.0%)	28 (96.6%)	5 (100.0%)
**Culture Results** (N, % Positive)	16 (64.0%)	9 (90.0%)	2 (6.9%)[2]	0 (0%)[3]
**Vasoactive-Inotropic Score** (Maximum, Day 1)	19.1 ± 24.8	22.2 ± 20.8	12.6 ± 15.3	N/A
**Oxygenation Index**[1] (Maximum, Day 1)	8.6 ± 9.9	5.0 ± 2.9	4.9 ± 5.1	N/A
**Serum Creatinine** (Maximum, day 1; mg/dL)	0.9 ± 0.7	0.4 ± 0.4	0.5 ± 0.4	0.6 ± 0.3
**Vasoactive-Inotropic Infusion** (Duration, Days)	2.2 ± 4.1	1.1 ± 1.5	3.5 ± 9.4	N/A
**Mechanical Ventilation** (Duration, Days)	1.8 ± 3.0	3.1 ± 4.1	3.7 ± 10.8	2.9 ± 1.6

Variable	Sepsis (N=25)	Sepsis + Viral (N=10)	SIRS (N=29)	Viral (N=5)
**PICU Stay** (Duration, Days)	6.1 ± 7.0	5.3 ± 3.6	5.6 ± 11.1	4.2 ± 0.8
**Hospital Stay** (Duration, Days)	21.4 ± 23.2	15.8 ± 6.2	11.8 ± 20.8	9.3 ± 6.5
**Mortality** (n, %)	0 (0.0%)	0 (0.0%)	1 (3.4%)	0 (0%)

[1]Oxygenation Index data available for only 10/25 sepsis patients, 3/10 sepsis+viral patients, and 22/29 SIRS patients.

[2]Two SIRS patients tested positive for MRSA at PICU admission (as an aspect of routine MRSA surveillance screening), but nonetheless did not display signs or symptoms of sepsis.

[3]All five virally infected patients tested negative for MRSA at PICU admission. Four of the patients also had blood cultures taken, which were negative. The fifth patient (VIR_005GAPPSS) did not have a blood culture taken, as this was deemed unnecessary by the attending physician.

<u>Reference</u>

Zimmerman, J.J. *et al*. Diagnostic Accuracy of a Host Gene Expression Signature That Discriminates Clinical Severe Sepsis Syndrome and Infection-Negative Systemic Inflammation Among Critically Ill Children. *Crit. Care. Med.* **45**, e418-e425 (2017)

Supplementary Table S4 | GAPPSS study line data (gene expression values from RNA-seq)

Subject	Class	ADGRE5	OASL	IL16	ISG15	Viral Signature
CPB_001	Control	176.39	7.28	133.13	47.57	-254.67
CPB_002	Control	280.42	23.83	119.86	50.77	-325.68
CPB_003	Control	302.47	26.02	90.19	127.1	-239.54
CPB_004	Control	240.26	11.93	83.5	41.67	-270.16
CPB_005	Control	223.17	15.69	116.21	42.69	-281
CPB_006	Control	209.59	7.58	89.39	30.58	-260.82
CPB_007	Control	246.76	10.89	90.32	51.94	-274.25
CPB_008	Control	318.92	13.64	84.82	37.17	-352.93
CPB_009	Control	317.32	6.69	104.2	34.43	-380.4
CPB_010	Control	169.97	18.14	107.57	70.69	-188.71
CPB_011	Control	457.23	8.43	54.89	10.18	-493.51
CPB_012	Control	620.43	23.2	115.85	39.67	-673.41
CPB_013	Control	332.75	17.9	85.16	77.79	-322.22
CPB_014	Control	725.22	149.58	65.05	709.15	68.46
CPB_015	Control	355	25.65	124.73	89.36	-364.72
CPB_017	Control	322.9	14.16	121.35	31.28	-398.81
CPB_018	Control	197.73	128.06	106.34	677.06	501.05
CPB_019	Control	340.38	30.49	66.26	193.94	-182.21
CPB_020	Control	608.92	42.5	79.69	312.64	-333.47
CPB_021	Control	387.71	10.5	78.72	30.86	-425.07
CPB_022	Control	581.26	3.98	92.82	19.39	-650.71
CPB_023	Control	301.12	32.68	97.96	90.38	-276.02
CPB_024	Control	559.9	14.44	91.63	13.69	-623.4
CPB_025	Control	348.41	23.62	67.15	140.38	-251.56
CPB_026	Control	391.56	16.37	81.35	80.27	-376.27
CPB_027	Control	690.4	6.27	43.34	27.2	-700.27
CPB_028	Control	482.12	19.55	73.77	77.36	-458.98
CPB_029	Control	459.74	25.66	87.87	54.95	-467
CPB_030	Control	576.02	15.08	94.58	24.71	-630.81
SEP_001	Sepsis	734.23	42.91	72.78	103.7	-660.4
SEP_002	Sepsis	323.16	80.95	96.77	300.08	-38.9
SEP_006	Sepsis	587.01	42.04	44.84	226.75	-363.06
SEP_007	Sepsis	615.62	44.54	65.19	70.05	-566.22
SEP_008	Sepsis	201.46	33.1	79.52	112.64	-135.24

Subject	Class	ADGRE5	OASL	IL16	ISG15	Viral Signature
SEP_009	Sepsis	546.2	2.98	79.73	34.01	-588.94
SEP_010	Sepsis	516.49	9.53	108.12	21.03	-594.05
SEP_011	Sepsis	269.19	45.89	69.62	212.9	-80.02
SEP_014	Sepsis	318.35	14.69	109.28	42.69	-370.25
SEP_015	Sepsis	708.23	61.55	41.23	454.82	-233.09
SEP_016	Sepsis	161.53	11.25	70.66	22.97	-197.97
SEP_018	Sepsis	382.84	5.97	125.4	29.11	-473.16
SEP_020	Sepsis	239.93	10.55	90.42	27.99	-291.81
SEP_021	Sepsis	361.91	53.8	89.16	138.66	-258.61
SEP_023	Sepsis	274.18	126.03	96.77	555.2	310.28
SEP_024	Sepsis	209.76	65.63	21.63	588.67	422.91
SEP_025	Sepsis	111.02	19.84	66.5	75.49	-82.19
SEP_026	Sepsis	618.22	16.16	100.35	9.35	-693.06
SEP_027	Sepsis	258.99	15.76	91.88	57.62	-277.49
SEP_030	Sepsis	482.48	66.08	55.32	212.13	-259.59
SEP_032	Sepsis	20.48	4.32	12.29	103.6	75.15
SEP_033	Sepsis	121.4	4.23	99.62	34.07	-182.72
SEP_034	Sepsis	132.63	1.9	55.26	53.23	-132.76
SEP_035	Sepsis	336.71	28.69	89.98	18.89	-379.11
SEP_038	Sepsis	653.71	21.68	73.58	120.27	-585.34
SEP_004	Sepsis + VIR	278.49	25.27	63.09	140.37	-175.94
SEP_005	Sepsis + VIR	147.36	2.66	29.31	39.02	-134.99
SEP_012	Sepsis + VIR	547.04	83.34	86.02	137.19	-412.53
SEP_013	Sepsis + VIR	223.61	17.38	61.35	104.99	-162.59
SEP_019	Sepsis + VIR	762.82	12.35	22.63	11.11	-761.99
SEP_022	Sepsis + VIR	91.46	4.28	23.22	25.33	-85.07
SEP_029	Sepsis + VIR	625	27.95	45.7	41.48	-601.27
SEP_031	Sepsis + VIR	219.06	24.22	67.77	82.42	-180.19
SEP_036	Sepsis + VIR	382.89	13.17	74.5	46.11	-398.11
VIR_001	Sepsis + VIR	121.06	2.4	70.88	42.55	-146.99
VIR_002	Virus	135.81	10.02	121.82	68.08	-179.53
VIR_003	Virus	132.96	13.03	133.68	85.74	-167.87
VIR_004	Virus	158.27	153.17	90.71	979.34	883.53
VIR_005	Virus	326.68	87.35	86.8	356.97	30.84
VIR_006	Virus	242.85	17.6	82.16	50	-257.41

Supplementary Table S5 | Line Data from Publication of Tsalik et al. (2016)

Reference: Tsalik EL, Henao R, Nichols M, Burke T, Ko ER, McClain MT, Hudson LL, Mazur A, Freeman DH, Veldman T, Langley RJ, Quackenbush EB, Glickman SW, Cairns CB, Jaehne AK, Rivers EP, Otero RM, Zaas AK, Kingsmore SF, Lucas J, Fowler VG Jr, Carin L, Ginsburg GS, Woods CW. Host gene expression classifiers diagnose acute respiratory illness etiology. Sci Transl Med. 2016 Jan 20;8(322):322ra11. doi: 10.1126/scitranslmed.aad6873. PMID: 26791949

**Sample ID**	**IL16**	**LAMP1**	**OASL**	**ADGRE5**	**ISG15**	**PLA2G7**	**CEACAM4**	**PLAC8**	**Class**
GSM1561860	0.059815585	0.274158407	0.50891091	-0.776560669	-0.682263467	0.223726943	0.706357352	0.316661843	non-infectious illness
GSM1561861	0.179763471	0.389000161	-1.103723546	-0.262358877	-0.692893241	0.122253647	0.925265702	-0.011855649	non-infectious illness
GSM1561862	-0.030925531	0.265241563	-0.373336745	0.516804976	-1.556452047	-0.882908754	2.479717818	-1.233795252	non-infectious illness
GSM1561863	-1.05533669	0.140891783	-0.864408007	-0.166554059	-1.208798893	-0.678267009	1.105347207	-2.920079114	non-infectious illness
GSM1561864	-1.094348801	0.173521055	-0.619448509	-1.26580111	0.159651722	0.582763771	1.46857306	0.510243068	bacterial
GSM1561865	-0.713155043	0.260460692	0.043321397	-0.142623852	0.695551108	0.216810362	1.297664315	1.446174338	non-infectious illness
GSM1561866	-0.146616569	0.246766312	-2.912627287	-0.150123383	-2.153702916	-0.653770311	-0.816774276	-0.644551662	non-infectious illness
GSM1561867	-9.50E-05	0.164191235	1.915168926	-0.020566124	2.88248745	-0.37184727	0.11490453	0.659526597	non-infectious illness
GSM1561868	-0.908662721	0.646113743	-0.958758438	0.54909464	-1.549606286	0.49624242	-1.026865943	0.85727899	bacterial
GSM1561869	-0.747548581	0.231601862	-0.963673785	0.124063406	-1.456188819	-0.377049717	1.640747752	1.264295762	bacterial
GSM1561870	-0.390410057	0.228851664	3.268729474	0.15228986	3.821574773	-0.636970686	0.881153563	1.142557236	viral
GSM1561871	0.288603221	0.330173678	-2.236217383	-0.771193875	-1.861209483	-0.708363297	0.010251072	1.652438804	bacterial
GSM1561872	-0.380133983	0.570590448	0.644524012	-0.037288519	1.360398266	1.004854833	0.759447697	1.000343576	bacterial
GSM1561873	-1.043331597	0.361938301	2.563753608	-0.008310878	3.640462841	1.069529923	0.665977591	1.562135319	viral
GSM1561874	-0.603488134	0.379509796	-0.440320561	-0.259112119	-1.339022006	-0.526398058	0.470389695	1.446580271	bacterial
GSM1561875	-0.502193471	0.465646067	-0.564837822	0.070275565	-0.262188619	-0.616792452	0.792552759	-2.305901295	bacterial
GSM1561876	-0.736615173	0.369382726	1.524674661	-0.485916491	1.562484862	-0.644332869	2.417970448	1.721581555	bacterial
GSM1561877	-0.055648609	0.263927612	1.481270617	-0.272171498	2.853630459	-0.062263768	-0.704966728	0.613936163	bacterial
GSM1561878	-0.274481268	0.467374407	1.558868667	0.656902835	1.504452941	-0.702687518	1.035813649	1.535084554	bacterial

Sample ID	IL16	LAMP1	OASL	ADGRE5	ISG15	PLA2G7	CEACAM4	PLAC8	Class
GSM1561879	-0.408535532	0.246634635	0.273630022	-0.552582083	0.797804115	-0.728983441	0.639835611	1.414220731	bacterial
GSM1561880	-0.956562356	0.038308164	-1.251461549	-0.673906442	0.083883973	-0.632490565	-0.438950064	-1.405561318	bacterial
GSM1561881	-0.551596467	0.317823024	-0.259330265	-0.12454135	-0.669024776	0.192943904	-0.167954739	0.161444602	bacterial
GSM1561882	-0.503136291	0.210590115	-1.895395676	0.062815764	-2.78399681	-0.393941221	1.992707584	-0.598819819	bacterial
GSM1561883	-0.417899583	0.257549051	-1.261856908	-0.432230098	-2.51383694	-0.392585056	-0.462504218	0.301344055	bacterial
GSM1561884	0.599121479	0.939661606	-1.588693574	0.76952388	-3.098518126	-0.495139694	0.75593203	-0.891352301	bacterial
GSM1561886	-0.337651171	0.818012757	-2.834750468	-1.546977593	-1.54855532	-0.70902106	0.784212969	1.778140402	bacterial
GSM1561887	0.236622531	0.645393768	-0.697871311	1.007049052	-2.263098907	-0.969037552	1.845041037	-0.447340945	bacterial
GSM1561888	-0.38723098	0.950726984	-1.404005589	0.384856748	-2.432063954	-0.762579926	-0.223971894	1.501708429	bacterial
GSM1561889	-0.094481274	0.804263645	-2.347224504	-0.029543022	-3.458456805	0.514907461	-0.158067356	1.247751983	bacterial
GSM1561890	-0.412919865	0.760255786	-1.02780692	0.268080134	-0.798714615	0.199512181	-0.103087847	0.229512573	bacterial
GSM1561891	-0.367533238	0.830428547	-0.647006007	-0.296498179	-0.280569639	-0.832919623	-0.635166606	1.52047341	bacterial
GSM1561892	-0.233651235	0.632798156	-0.792548292	-0.828247124	-1.318822405	-0.21025737	-1.179324797	0.092961562	bacterial
GSM1561893	0.336671347	0.867260695	-1.951934734	0.08640853	-3.009618625	-0.809681243	-1.109070386	-0.554037608	bacterial
GSM1561894	-0.417402463	0.894417704	-2.075272143	0.433677707	-3.559938975	-0.666526988	1.614862431	1.947602339	bacterial
GSM1561895	-0.090402806	0.834355751	0.849835934	0.285260437	1.427063781	-0.337270441	-0.006035402	0.412008569	bacterial
GSM1561896	-0.863653605	0.77472328	2.303797269	0.672363974	3.495083138	0.903928154	-0.420367343	0.893779555	bacterial
GSM1561897	-0.245777673	0.405971843	-2.167913818	-0.471724508	-2.833476863	-0.126345457	0.548789084	-1.229931944	bacterial
GSM1561898	0.184432714	0.763750802	-1.053527423	-0.027766243	-1.629604166	-0.52462154	-0.720607061	-0.505865882	bacterial
GSM1561900	-0.883032006	0.855944218	-2.02176698	0.537015327	-2.470079496	1.503985831	0.621524663	0.389071787	bacterial
GSM1561901	-0.208181087	0.78175992	0.803354979	0.309699828	1.107417999	-0.579429334	1.524065206	1.913407948	bacterial
GSM1561903	-0.021299531	0.534587758	-1.531611089	0.245553146	-2.767478034	-0.678056525	0.136117717	0.450162054	bacterial
GSM1561904	-0.316550584	1.074947802	-2.340956274	-0.087658231	-3.056163231	-0.56869605	0.890624732	1.502167191	bacterial
GSM1561905	0.000330827	0.623831836	-0.906286556	0.468986387	-1.8649192	-0.90638714	1.416033045	0.405102	bacterial
GSM1561906	-1.274248482	0.799655848	-0.222226609	-0.180236423	0.699537765	-0.223772481	0.040118476	1.648686727	bacterial
GSM1561907	-1.081569893	1.045396704	-2.142367743	0.563970944	-3.908024109	0.537645065	0.862017573	1.120610619	bacterial
GSM1561908	-0.38361029	0.55233709	-0.103406462	0.017410141	-2.182516753	-0.219721179	0.515040929	0.140228418	bacterial
GSM1561909	0.059528862	0.339566234	0.201702324	0.086399637	0.183738586	-0.735571768	0.306991694	1.185885752	bacterial
GSM1561910	0.2477717	0.647225178	-1.759132203	-0.819087309	-1.934828704	-0.279729112	0.146772185	-1.510443769	bacterial

Sample ID	IL16	LAMP1	OASL	ADGRE5	ISG15	PLA2G7	CEACAM4	PLAC8	Class
GSM1561911	-0.920031738	0.578559163	-2.296680037	-1.574743257	-1.73278101	-0.364561006	-0.596759353	1.851945007	bacterial
GSM1561912	-0.216855855	0.72141186	-0.315847718	0.258666202	-0.74678652	-0.370013242	0.594157347	1.018090112	bacterial
GSM1561913	-0.761680057	0.719850099	-1.424255698	-0.285331769	-0.551951437	-0.457815414	0.922786376	1.374405924	bacterial
GSM1561914	-0.367506812	0.856539572	-1.221892251	-0.029473502	-3.281072887	-0.781267405	0.980984843	0.907499319	bacterial
GSM1561915	-0.120555803	0.724948052	0.123088249	0.75467191	0.032127235	-0.777644714	0.445124499	1.029479983	bacterial
GSM1561916	0.495756832	0.246935824	0.889098898	-0.2140423	2.007634591	-0.788586945	-0.808552072	-0.944443694	bacterial
GSM1561917	-0.519856994	0.658906106	-1.512867739	0.774839257	-2.182654405	-0.813508137	0.43030932	1.514242378	bacterial
GSM1561918	-0.37178704	0.904007763	1.15282663	-0.368888608	0.803511097	-0.786346676	1.268182132	1.764688218	bacterial
GSM1561919	-0.045682358	0.5424476	-0.719691094	-0.129043089	-2.89267036	-0.655559599	1.219454871	1.756362533	bacterial
GSM1561920	-0.458384114	0.501216698	-1.137943183	0.619155906	-0.879614575	-0.615427326	0.12998428	-0.889200649	bacterial
GSM1561921	-0.680746335	0.447703078	-0.16430331	-0.286357993	0.014385595	-0.405292126	-0.456643349	0.050706378	bacterial
GSM1561922	-0.496986131	0.585423799	-2.146355114	-0.57489464	-2.397828459	0.006575514	0.293185135	1.179404009	bacterial
GSM1561923	-0.513087908	0.354865396	-2.135173523	-1.066486297	-1.748137657	0.774353489	0.561981822	1.312006589	bacterial
GSM1561924	-0.227418622	0.289620926	-0.252024837	-0.844568353	-0.791723106	-0.570996173	0.026700752	1.159323191	viral
GSM1561925	0.245835969	0.501964978	1.751117287	-0.144977654	1.932962902	0.323342944	-0.284217068	-0.479942726	viral
GSM1561926	0.23111164	0.684908258	2.300245254	-0.369342468	2.66274804	0.277063008	-0.852924593	0.305136951	viral
GSM1561927	0.740029131	0.125279585	2.002002866	-0.277404643	2.463692095	1.637966148	-0.260140233	0.090845341	viral
GSM1561928	0.476514271	0.69605804	1.249918972	-0.303390549	1.676047052	0.251283639	-0.249047848	0.045493746	viral
GSM1561929	0.126226463	0.66795411	2.184226633	-0.61675659	3.009286665	0.300065782	-0.461447493	0.327861889	viral
GSM1561930	-0.044862574	0.551678309	0.53509434	-0.591275427	1.301412993	0.095407141	-0.554584068	-0.213670967	viral
GSM1561931	0.333985769	0.666583284	1.610955121	-0.241014547	1.913695492	0.124831398	-0.22165998	-1.318521647	viral
GSM1561932	0.22061472	0.720034976	1.572376963	-0.142099217	2.134746677	0.723652102	-0.414369011	-0.112537168	viral
GSM1561933	0.599831671	0.602008929	0.438888019	0.149807341	0.498327635	0.438081079	-0.100515004	-0.837845219	viral
GSM1561934	0.380666934	0.722165245	-1.414126784	-0.241381417	-1.562600628	-0.085596762	-0.230576355	-1.092867838	viral
GSM1561935	0.057583777	0.711493136	1.349532794	-0.41522971	1.81364173	1.01057994	-0.517967826	-0.066238113	viral
GSM1561936	0.619786357	0.750940096	0.235354816	0.320945841	0.593524693	0.05785449	-0.373318593	-0.876653577	viral
GSM1561937	0.254549032	0.696768014	-0.669233183	0.295448625	-0.696645433	0.058991112	-0.534149697	-1.737497939	viral
GSM1561938	0.38436991	0.440000523	-0.392992606	0.074587219	-1.15024133	-0.368628397	0.050880445	-0.925757364	viral
GSM1561939	0.476809816	0.618817667	-0.284785316	-0.300297463	-0.893570982	-0.414430869	0.294889587	-0.749790274	viral

Sample ID	IL16	LAMP1	OASL	ADGRE5	ISG15	PLA2G7	CEACAM4	PLAC8	Class
GSM1561940	0.468687455	0.564661991	-0.596667425	-0.277477185	-0.524306388	0.242016521	0.116441681	-0.579105551	viral
GSM1561941	0.117736791	0.641151927	0.184069035	-0.737749314	0.580930774	0.486876075	-0.355990473	-0.033178686	viral
GSM1561942	0.302522512	0.516539605	0.212123224	-0.08871276	1.009155993	0.399108004	-0.532066342	0.026563624	viral
GSM1561943	0.386733532	0.64444307	-0.353835323	0.212442283	-0.658296731	-0.530584005	-0.719455227	-0.817507658	viral
GSM1561944	0.45803718	0.333012116	1.355598645	0.179607753	1.844252899	-0.025504312	-0.815853009	0.143301684	viral
GSM1561945	0.463811234	0.71288201	1.69739316	0.31362784	1.377678814	0.176854174	-0.357419043	-0.762253029	viral
GSM1561946	0.437711858	0.633489519	0.021525176	-0.420047503	-0.186646564	-0.152919543	-0.143975103	-0.727683998	viral
GSM1561947	0.545412128	0.451458308	1.308425241	0.120633895	0.856568113	-0.302452378	0.019306788	-1.024943334	viral
GSM1561948	0.259847452	0.726390167	-1.197529245	-0.189006954	-1.13598459	0.416019423	0.180014879	-0.938141456	viral
GSM1561949	0.079736748	0.763024466	1.845860951	-0.130152484	2.035267736	0.505079247	-0.703684405	0.124547137	viral
GSM1561950	-0.767001477	0.475424996	-0.427471909	-0.184647943	-0.351913202	-0.334092789	-0.433190365	-0.010232665	viral
GSM1561951	0.378530366	0.310270162	0.597086558	0.287420214	0.621054262	0.093545089	-0.539813568	-0.885622309	viral
GSM1561952	0.487447479	0.718153165	-1.021604027	-0.483576766	-1.370082118	0.343774597	-0.559556958	-0.355677976	viral
GSM1561953	-0.423293316	0.771939013	3.302870095	-0.150883105	3.539589353	0.170057176	-0.422596907	1.456217815	viral
GSM1561954	0.649974506	0.795612475	0.907412646	0.52686856	0.825938286	0.123021768	0.862215001	-0.796706255	viral
GSM1561955	0.032055776	0.616500637	2.477392747	0.642816108	3.019785503	0.186566865	-0.076371658	-0.184578287	viral
GSM1561956	-0.33014399	0.722496252	1.42314333	0.246531369	1.392783343	-0.000616066	-0.258100918	-0.431845491	viral
GSM1561957	0.425594407	0.339804974	1.350612594	0.609294955	1.080204418	0.289778214	-0.253333335	-0.16340583	viral
GSM1561958	0.49236789	0.61751574	1.626800149	0.131116192	2.00709034	0.230149605	0.099529558	0.048512792	viral
GSM1561959	0.06916934	0.678282876	1.77168803	-0.080916893	2.65647167	0.100365353	0.605859232	0.412736726	viral
GSM1561960	0.719580312	0.287285662	1.55039428	0.366452202	1.682129407	-0.350894186	-0.183482086	-0.930206375	viral
GSM1561961	0.190873332	0.66747288	1.864208641	0.271305526	1.779235958	-0.310056571	-0.218041397	-0.08913373	viral
GSM1561962	0.177860786	0.459641428	0.369065226	0.479024819	0.956395563	0.199202884	-0.266398971	-0.829755156	viral
GSM1561963	0.341987178	0.711726229	0.925189	-0.793314567	1.77600471	0.202403071	-1.214653601	0.455469369	viral
GSM1561964	-0.179887199	0.898168913	2.710620989	0.005047085	2.886427242	0.113952339	-0.902612998	1.458047621	viral
GSM1561965	0.130790351	0.621027002	0.684087935	-0.615050193	0.530476331	-0.098843301	-0.109325598	-0.474779279	viral
GSM1561966	0.326134629	0.683067618	1.382904087	-0.5699218	2.173302221	0.536592627	-0.327470238	0.271066559	viral
GSM1561967	0.236502606	0.795599871	1.447037931	-0.977851132	2.053202531	-0.055983917	-0.199800119	-0.006859468	viral
GSM1561968	0.302953466	0.583050592	2.040586461	-0.776021467	2.703440452	-0.449537665	-1.972947907	0.455344704	viral

Sample ID	IL16	LAMP1	OASL	ADGRE5	ISG15	PLA2G7	CEACAM4	PLAC8	Class
GSM1561969	0.29416408	0.858200395	2.497241302	-0.375921469	3.198274938	0.438378541	-0.608501833	1.041870591	viral
GSM1561970	0.629985395	0.502276435	-0.440007863	0.359275085	-1.342596822	0.034489809	1.091851421	-0.466944074	bacterial
GSM1561971	0.097545306	0.685650888	2.492461358	-0.103762782	3.361724056	-0.132398587	-0.439648723	1.612039996	viral
GSM1561974	0.166147015	0.912072519	3.263760334	0.460925087	3.646942482	0.214023885	0.394875364	1.264295762	viral
GSM1561975	-0.205099222	0.435085195	-0.279305349	-0.585611195	-1.20868881	-0.095925627	-0.07736924	-0.321021135	bacterial
GSM1561976	0.289994138	0.498162864	0.060955068	-0.313327883	0.447906205	0.07134447	-0.467458453	0.174663509	non-infectious illness
GSM1561978	0.807226802	0.500278753	0.845665016	-0.021407991	1.082489902	-0.477310126	-0.590780417	-0.575703797	viral
GSM1561979	-0.432752184	0.812277379	2.341987513	0.289857711	2.931578186	0.149823569	0.329754696	1.153642925	viral
GSM1561980	-0.412899099	0.564860298	1.288989772	0.101061783	2.711036357	-0.482231519	-0.792546076	-0.223626587	viral
GSM1561981	0.516058553	0.440715714	2.510428259	0.322977861	3.608300477	-0.446354764	-1.16412968	0.988834682	viral
GSM1561982	-0.011940221	0.292834032	2.891795012	1.04445315	3.569950713	-0.082971798	-0.658378572	1.158741051	viral
GSM1561984	-0.409287282	0.582678646	2.916545096	0.204100372	3.548988414	-0.509307231	-0.249014905	1.558398607	viral
GSM1561985	0.194957376	0.703254161	0.963477708	-0.882432498	1.930771824	-0.585370855	-0.939114658	-0.129285586	viral
GSM1561986	0.06876023	0.668061232	0.106476701	0.140409842	-0.101227436	0.555587521	-0.416708692	0.89592869	viral
GSM1561987	-0.078964936	0.49412011	0.812145049	-0.043995648	1.21199768	0.342774185	-0.510107925	-0.493062963	viral
GSM1561988	-0.831249647	0.862005505	-1.417019386	1.212351123	-2.025684021	0.448304537	0.403726501	0.350063433	viral
GSM1561989	0.027567272	0.727776768	2.517055398	0.429043595	3.337446132	0.475326489	-0.353023388	0.121246042	viral
GSM1561990	-0.00100833	0.866399377	1.105582438	0.662181068	1.485439326	0.386430796	-0.301175404	-0.096631115	viral
GSM1561991	0.514253505	0.551264883	0.881257565	-0.640396016	2.283655103	-0.49408951	-1.453532518	0.77622386	viral
GSM1561992	0.301677755	0.74090258	2.636744252	0.37110577	3.728008895	-0.544952539	-0.576437024	1.025590703	viral
GSM1561993	0.15423811	0.323262809	2.677021321	0.292190028	3.598581759	0.418739803	-0.935597058	1.256769797	viral
GSM1561994	-0.666977751	0.309319187	2.611495788	-0.564731419	3.356229236	-0.40368744	-1.361313073	1.195936331	viral
GSM1561995	0.52446309	0.665376551	0.95607408	-0.246066925	2.24536792	-0.111476595	-1.577384506	0.84562432	viral
GSM1561996	-0.037942396	0.636191017	1.926389482	0.243773421	2.61681603	0.420956372	-0.666658543	0.093227924	viral
GSM1561997	-0.358843322	0.450344408	2.080539088	-1.201386846	2.696088583	-0.096309041	-0.351495894	0.761196315	viral
GSM1561998	-0.262317707	0.711903617	0.92643325	0.839866106	1.386222347	-0.623425851	-0.567607122	1.063119665	bacterial
GSM1561999	0.033377433	0.539354045	2.12003442	1.019643256	3.170009884	-0.075058165	0.253882271	0.827754027	viral
GSM1562000	0.091902682	0.416465146	2.46622141	0.922429413	3.582257033	0.137435337	-0.168035716	0.58599772	viral
GSM1562001	0.353464893	0.767788676	2.829121329	-0.090556822	3.4986358	0.420292677	-0.541322916	0.955575834	viral

Sample ID	IL16	LAMP1	OASL	ADGRE5	ISG15	PLA2G7	CEACAM4	PLAC8	Class
GSM1562002	-0.229484051	0.55799716	2.897040501	0.262218047	3.421365708	-0.088877328	-1.30493452	0.872290493	viral
GSM1562003	0.38528249	0.711335501	1.847590237	-0.074441752	2.739636262	0.280002963	-0.604521579	0.158768857	viral
GSM1562004	0.35351771	0.653175052	1.146498027	-0.069041731	1.789174831	-0.096541697	-0.800071113	-0.22243121	viral
GSM1562005	-0.663403144	0.609201564	0.897719044	-0.043398449	1.671523778	-0.464079133	0.749894239	1.555049095	viral
GSM1562006	-0.041883306	0.399157834	-0.01104949	-0.616949055	0.720159837	-0.087057857	-0.844037959	-0.503567626	viral
GSM1562007	0.385033152	0.414632606	-1.052130405	0.244292545	-1.06623703	0.444429458	-0.346274591	-1.191695291	viral
GSM1562008	0.208448057	0.641567375	1.887332417	0.17863024	2.558138561	0.236836522	-0.72545589	1.181322476	viral
GSM1562009	0.336855682	0.668668476	2.864015937	0.176853494	3.593455052	-0.258135309	-0.519304248	1.004549221	viral
GSM1562010	-0.098464037	0.487258378	0.179116009	-0.057176248	0.556978231	-0.541086457	0.062059097	0.414075466	viral
GSM1562011	0.014207025	0.554024329	3.186632841	0.291056561	3.700771037	-0.483456208	0.898425091	1.264306852	viral
GSM1562012	-0.121164596	0.564345921	1.074731145	-0.997396509	1.227847066	0.117460808	0.544770739	0.644521243	viral
GSM1562013	0.453948644	0.76819399	-0.139263272	-0.076054645	0.542217781	0.503177639	-0.102431805	-0.252440321	viral
GSM1562014	-0.111547367	0.792612167	2.797117823	0.873973622	3.438249377	-0.692818357	0.780282057	0.728158493	viral
GSM1562015	0.125717903	0.770572889	2.180452407	0.971207062	2.595597243	-0.699968084	1.319833906	-0.751896107	viral
GSM1562016	0.23723718	0.267161396	1.204910812	0.320421574	1.347500316	-0.334518549	-0.638248041	0.72817259	non-infectious illness
GSM1562017	0.396966184	0.614378002	1.131601868	0.215717671	1.519960872	0.543390634	-0.965389802	0.732879926	bacterial
GSM1562018	-0.598326479	0.169990566	-0.115141334	0.879000065	0.605803959	0.583963754	-0.257603242	0.782315542	non-infectious illness
GSM1562019	0.340775693	-0.242073986	-1.129147949	-0.86304343	-0.245556362	-0.619073508	-1.022415712	-0.085833262	non-infectious illness
GSM1562020	0.240823949	0.927058247	-1.120903435	0.405612347	-2.359720715	-0.454628584	-0.008631303	-1.190365504	non-infectious illness
GSM1562021	0.11352178	0.459570912	-0.053360405	0.0820173	-0.604757794	-0.379539607	-0.828138902	0.226128866	non-infectious illness
GSM1562022	0.410403455	-0.362531635	-1.072143974	0.187203762	-1.81673766	-0.538625357	0.098222182	-0.662723254	non-infectious illness
GSM1562023	-0.236687933	-0.38331792	2.66996151	-0.012520435	2.957684986	-0.134949801	-0.008270814	0.580557157	bacterial
GSM1562024	0.089451993	-0.551127407	-1.197164001	-0.647556904	-1.24544669	0.818830373	0.198282645	-1.214254092	non-infectious illness
GSM1562025	-0.45600537	0.113273648	-0.22375721	-0.55384219	-0.507238873	0.185707452	-0.393739297	-0.304568957	non-infectious illness
GSM1562026	0.477574552	1.037987736	-0.336330833	0.855211613	-1.855633267	-0.533928988	0.42037432	-1.000622462	non-infectious illness
GSM1562027	0.623768056	0.081019428	-0.917823257	0.360966084	-2.951206692	-0.197882292	-0.348620965	-0.437912653	non-infectious illness
GSM1562028	-0.355434214	-0.875294658	-1.191890182	-0.531038883	-1.351507672	0.208547475	-0.209174707	-1.015240381	non-infectious illness
GSM1562029	0.112022127	0.322673825	-0.156238975	0.268617031	-0.40519286	0.178134801	-0.72234781	-0.660402964	non-infectious illness
GSM1562030	-0.079069888	0.660717991	0.232496796	0.148987171	-0.1603942	0.166621568	0.157997534	1.565453817	viral

Sample ID	IL16	LAMP1	OASL	ADGRE5	ISG15	PLA2G7	CEACAM4	PLAC8	Class
GSM1562031	-0.20101956	0.233341244	2.878767548	0.265291569	3.31603574	0.734111669	-0.631167009	1.053015043	viral
GSM1562032	0.181555952	-1.532358143	0.570552406	-0.575492504	1.555005543	-0.708732722	-0.701056077	0.880430509	viral
GSM1562033	-0.336573874	0.200431851	3.321884689	0.201334355	3.52964925	0.874963167	-0.716085985	1.430135061	viral
GSM1562034	-0.039166247	-1.148424122	3.179683266	-0.216466549	3.482810489	-0.587668536	0.098419592	0.342665006	non-infectious illness
GSM1562035	0.15322801	-0.801988575	3.591009674	0.227621434	3.779696934	0.067482832	-0.340133264	1.475947668	viral
GSM1562036	-0.011219775	0.56260207	-1.420435371	-0.066918972	-1.496792324	0.041549566	0.43779032	1.173463155	bacterial
GSM1562037	-0.086421763	-0.707490894	3.126794396	0.259832737	3.234637729	0.165244939	-0.485381677	0.777803709	viral
GSM1562038	-0.286595141	0.17846587	0.3811689	-0.092082622	0.525372974	0.258990648	-0.063413217	0.551736166	bacterial
GSM1562039	-0.078450953	0.675437001	-2.111580791	-0.272637966	-2.340041645	-0.119644759	0.545722411	1.322560598	bacterial
GSM1562040	0.309640134	-1.177422497	-0.239031258	-0.366181019	0.00345277	0.115304353	-0.268296792	-0.303450297	non-infectious illness
GSM1562041	-0.57025746	0.079956082	0.498754082	-0.27253783	0.586841099	0.90638912	-0.183675363	-0.237655352	non-infectious illness
GSM1562042	-0.241974627	0.894265202	0.224204598	0.406110165	0.605613224	-0.47117388	-0.649512842	0.415507604	non-infectious illness
GSM1562043	0.412040914	0.28497031	-1.543068352	1.084900257	-2.331182964	-0.195746539	0.554525778	-0.986704539	non-infectious illness
GSM1562044	0.44737264	0.845376995	-0.526941719	0.514131901	0.321381084	-0.577947257	-0.395433023	0.48039942	non-infectious illness
GSM1562045	-0.610640322	-0.443706486	-0.172575416	-0.300601604	-0.070436009	0.354297521	0.501251624	-0.301245402	non-infectious illness
GSM1562046	-0.3984578	-0.085515949	-0.086893501	-0.379425647	-0.420922764	-0.042958376	-0.07836683	-0.604320238	non-infectious illness
GSM1562047	0.249530996	0.133837727	-0.845649185	-0.266015651	-0.23200465	-0.441419718	-1.176176622	-0.204317759	non-infectious illness
GSM1562048	-0.441526209	0.741659191	-0.781754913	0.03446996	-0.59189082	-0.389930365	0.648182318	-0.999640177	non-infectious illness
GSM1562049	-0.155116354	0.637796429	-1.288399162	-0.276549938	-0.939019688	0.511295202	-0.182271554	-0.531282137	non-infectious illness
GSM1562050	-1.252055918	0.744140652	-2.529670181	0.035496113	-2.525520809	0.36601341	0.897997305	0.117366125	non-infectious illness
GSM1562051	-0.620319163	1.339832333	-2.485129776	-0.093967083	-2.996839054	-0.3445698	0.005851195	1.425335218	non-infectious illness
GSM1562052	-0.360469138	-0.172783825	0.20011097	-0.087548855	0.068899624	-0.038123248	-0.282923295	-0.143120478	non-infectious illness
GSM1562053	-0.292613303	0.103437451	0.857036171	0.695100186	0.076947681	-0.422733424	1.206810401	-2.539065073	non-infectious illness
GSM1562054	-0.201843058	0.007395771	-1.109157028	0.647590039	-0.65895129	0.53869808	-0.857369974	-1.460831354	non-infectious illness
GSM1562055	-1.106272971	-0.206289264	0.707146225	-0.665502193	1.406656734	0.42531574	0.859660552	0.073758413	non-infectious illness
GSM1562056	-0.888291632	-0.540725681	1.338483391	0.216297505	2.425779812	-0.524781821	0.654285834	-1.134131144	non-infectious illness
GSM1562057	-0.44245175	1.363948849	0.864525375	0.409420368	1.058886997	-0.288320181	0.314258025	0.602826406	non-infectious illness
GSM1562058	-0.584068095	-1.235488865	-0.421562614	-0.461591366	-1.045679577	-0.188326476	-0.026328638	-1.137935351	non-infectious illness
GSM1562059	0.061160768	0.972788838	-1.474241676	0.393288014	-1.785734661	-0.038684485	0.075484679	0.527385999	non-infectious illness

Sample ID	IL16	LAMP1	OASL	ADGRE5	ISG15	PLA2G7	CEACAM4	PLAC8	Class
GSM1562060	0.186598981	0.523853329	-1.337723198	0.437157986	-1.514266499	0.048381689	0.127276824	0.484080602	bacterial
GSM1562061	-0.016080653	0.261534718	-0.943569603	0.273654339	-1.101830013	0.276175859	-0.296610429	0.632579138	non-infectious illness
GSM1562062	0.160250391	0.015736607	-1.366068306	-0.062072399	-1.255316265	0.190770032	-0.723217561	-0.665643404	non-infectious illness
GSM1562063	0.24712869	0.299109486	-0.66755909	0.339134571	-2.854347714	-0.391788123	0.479978964	-0.331398676	non-infectious illness
GSM1562064	0.634940351	0.224233654	-0.461174431	-0.245438221	-0.732965954	0.059139135	-0.326144769	-0.334711741	non-infectious illness
GSM1562065	0.052465642	-0.163522693	-1.185577812	0.432730076	-1.534718044	-0.686618445	1.064902709	-1.326129626	non-infectious illness
GSM1562066	-0.360216414	0.968057659	0.119006691	0.945042012	-0.288177975	2.51489723	-0.011127069	-0.760869692	non-infectious illness
GSM1562067	0.453406538	0.857996014	-0.903023186	0.14670026	-0.822953709	1.310449483	-0.003901079	0.516611387	viral
GSM1562068	0.436010564	-0.178808185	-0.562284889	-0.186641338	-0.438320645	0.796449068	-0.745893075	-0.063819597	viral
GSM1562069	0.426971639	0.245551136	1.373427925	0.23954427	1.499699108	0.465439203	-0.626301362	-0.172592829	viral
GSM1562070	-0.4722741	-0.866071674	0.323142586	-0.649018886	0.526689998	-0.093416808	-0.720627382	-0.750569056	viral
GSM1562071	-0.496921336	-0.574450466	2.269587097	-0.0107075	2.176646702	-0.189399836	0.400293753	-0.032475184	viral
GSM1562072	0.424388135	0.273926762	-1.341449203	0.394908306	-2.481756695	0.649979475	0.546612189	0.439474394	non-infectious illness
GSM1562073	0.159643377	0.349020031	2.446074066	0.318027851	3.093736164	0.203317049	-0.788704469	0.747544576	viral
GSM1562074	0.171486105	0.557830837	-1.647472324	0.159675953	-2.71563197	0.828272226	-0.506837442	0.036629965	non-infectious illness
GSM1562075	0.371134008	1.427959469	1.017056999	0.225798962	0.690811626	-0.680235026	0.745147154	1.293082525	non-infectious illness
GSM1562076	0.206681455	0.13016322	-1.245826187	-0.2789799	-1.441368588	1.029157522	-0.381693437	-0.348560045	non-infectious illness
GSM1562077	0.513720716	0.420212773	0.300426064	-0.220073345	1.636674396	0.759541395	-1.646520212	-0.035824488	non-infectious illness
GSM1562078	-0.143170153	0.178173092	-1.876066151	-0.12084544	-2.231992253	-0.161523936	0.174686835	-0.787960718	non-infectious illness
GSM1562079	-0.157183444	0.643646267	0.323233185	0.982363102	0.426463596	-0.727424738	1.730046675	1.024346639	bacterial
GSM1562080	-0.124522959	-0.475565883	0.285680351	-0.199330929	0.237017147	-0.479628203	-0.07863672	0.65778475	non-infectious illness
GSM1562081	0.709620679	0.180553463	-1.292022171	0.224884388	-1.492948063	0.361056688	-0.516394212	-1.1677577	non-infectious illness
GSM1562082	0.192173208	0.519466427	1.114366112	0.36717355	1.136822216	1.072227838	0.059451506	0.354977895	non-infectious illness
GSM1562083	0.115283693	0.097885159	0.100747643	0.490414021	-0.830143459	-0.065962908	0.179434226	-0.398663233	non-infectious illness
GSM1562084	-0.053428446	-1.112634632	2.092086372	-0.566974285	2.598308079	-0.161465762	0.015840933	0.038515379	non-infectious illness
GSM1562085	0.38180467	0.565966633	-0.431641045	0.12305772	-1.167039402	0.836972597	0.206839011	-0.103862475	non-infectious illness
GSM1562086	0.033201421	0.042094258	1.904483376	0.767644284	2.335835926	-0.682341216	1.459799216	1.262154867	bacterial
GSM1562087	0.839255537	0.761281994	-1.455828133	0.261327145	-3.140339382	-0.515213129	-0.571096017	-0.466017435	non-infectious illness
GSM1562088	0.231695856	0.141311672	-0.271162453	-0.736315372	-1.162003048	0.395461919	-0.662569597	1.157959272	viral

Sample ID	IL16	LAMP1	OASL	ADGRE5	ISG15	PLA2G7	CEACAM4	PLAC8	Class
GSM1562089	0.42583772	1.113298443	0.522059021	0.394110081	-0.013733395	0.002097013	0.323429511	-0.521347712	viral
GSM1562090	0.094321455	-0.802574131	2.984878121	0.652089474	3.108071796	-0.242220519	-0.400763109	0.915273	viral
GSM1562091	-0.380467688	-0.783649418	-0.192280235	-0.093332889	-0.953847416	-0.501773951	-0.221054628	0.055726339	viral
GSM1562092	-0.122703349	0.015146282	-0.161375465	-0.01385844	-0.391305454	-0.371125338	0.846027777	-0.074931811	viral
GSM1562093	0.255738462	-0.205244991	-0.779456558	0.607971546	-2.008127697	-0.564348814	0.206632063	-0.771446894	non-infectious illness
GSM1562094	0.220550264	0.101073292	-0.433479729	0.230488178	-0.942574032	-0.459877131	0.712857649	0.138650228	bacterial
GSM1562095	0.238411153	0.231298474	0.005040703	-0.060243252	-0.599708088	-0.055114863	-0.390585496	-0.696468066	non-infectious illness
GSM1562096	-0.658753191	1.239833859	-1.272590103	0.332904217	-2.195753582	-0.673453925	0.128220961	1.115626623	non-infectious illness
GSM1562097	-0.241076743	0.591718701	0.505282936	0.350707409	1.102319233	-0.454008696	1.351023123	-2.022740076	non-infectious illness
GSM1562098	-0.364647185	-0.748702976	0.401710091	-0.428746822	1.070219509	0.170184972	-0.210473611	-1.590915392	non-infectious illness
GSM1562099	0.323023046	0.459362057	-1.042956772	0.854185459	-1.50587035	-0.001605627	-0.332678392	-0.690880488	non-infectious illness
GSM1562100	-0.308412825	-0.484743091	0.095177231	-0.851084354	-0.260591992	0.333714368	-0.185808732	-0.702196787	non-infectious illness
GSM1562101	0.132019723	0.49971011	-1.038649025	0.630667088	-1.887392529	-0.336780188	0.952813551	-1.65039892	non-infectious illness
GSM1562102	0.310095991	0.790386227	0.212852059	0.424823162	-0.089452274	-0.239313719	-0.085774972	-0.882534693	non-infectious illness
GSM1562103	0.260725225	-0.23870084	-1.390259209	0.417265293	-1.654985912	0.189103487	0.005704329	-1.023335169	non-infectious illness
GSM1562104	-0.668193137	0.602636364	-0.25215477	-0.508943203	-1.029985912	0.509528997	0.085812018	0.067128469	non-infectious illness
GSM1562105	-0.589790141	-1.099159214	-2.26701683	-1.586915615	-2.792606838	-0.140218375	0.178195403	-2.183051775	non-infectious illness
GSM1562106	-0.027029788	-0.318165752	0.552099762	-0.776036861	1.765716068	0.09137285	-0.514512613	0.953025152	non-infectious illness
GSM1562107	-0.454610621	-0.004152271	-1.283473434	-1.043809536	-2.01949931	0.423472765	-0.261549547	-0.997744272	non-infectious illness
GSM1562108	-0.204847132	0.658044842	-0.328885498	0.186535237	-1.141370304	0.193036916	-0.24921663	1.104165365	non-infectious illness
GSM1562109	-0.500813281	-0.688380214	0.666912613	-1.676165226	0.30868005	-0.210767863	-0.058662012	-1.40552492	non-infectious illness
GSM1562110	-0.011381899	0.690009144	-0.335407677	0.090846417	-0.87496043	-0.47138798	0.170880721	-0.055140208	non-infectious illness
GSM1562111	0.033633436	0.290452984	-1.305135193	-0.122281673	-2.052813061	0.636210325	0.08756964	0.106782247	non-infectious illness
GSM1562112	0.484622205	0.379895237	-1.632350387	0.115477917	-1.835735806	0.512489679	0.186189101	0.117229749	non-infectious illness
GSM1562113	-0.166603838	-0.022727939	1.894084511	0.637270329	2.952773563	-0.076775668	-1.15451391	0.722979833	non-infectious illness
GSM1562114	0.210530485	-0.311255428	1.169255791	-0.412348392	1.213386051	-0.37961447	0.603338645	-0.413144778	non-infectious illness
GSM1562115	-0.406677996	0.841308621	-0.925714912	0.348846791	-1.9577365	-0.539050696	1.424558089	1.422426512	bacterial
GSM1562116	-0.205111777	-1.321566555	-0.834682884	-1.553578975	-0.752851971	0.638679864	-0.04881151	-0.406284998	non-infectious illness
GSM1562117	-0.095186984	-0.152697536	-1.03842205	-0.925792339	-0.720572003	0.196298482	-0.086453988	-0.623089503	non-infectious illness

Sample ID	IL16	LAMP1	OASL	ADGRE5	ISG15	PLA2G7	CEACAM4	PLAC8	Class
GSM1562118	0.312617506	-0.879142734	-1.518636169	-0.450407627	-0.58667899	0.457654836	0.406948493	-0.45624132	non-infectious illness
GSM1562119	-0.038895403	-1.00595853	0.14831501	0.004575131	-0.03750182	-0.398663161	0.266448424	-0.916956614	non-infectious illness
GSM1562120	0.205629076	-0.029660198	1.67119175	-0.229551914	2.27012109	0.051819685	-0.442587449	0.049487402	viral
GSM1562121	0.208617891	-0.921964618	0.774408875	-0.517692211	1.312670223	0.170049551	-0.221065118	-0.110842417	viral
GSM1562122	0.128967489	-1.454940769	2.017092285	-0.497889164	2.931358806	0.009595277	-0.913252428	0.92945986	viral
GSM1562123	0.766773428	-1.052518818	0.889340935	-0.615112903	1.550406925	-0.185809252	-0.197431161	0.220581342	viral
GSM1562124	0.210997785	0.198967961	2.334075508	0.673031208	2.473476879	-0.71132767	0.369192526	1.571229269	viral
GSM1562125	0.301453794	-0.341321918	0.042701302	-0.443418148	0.218554012	-0.542663214	-0.790875032	-0.11817808	viral
GSM1562126	-0.393760954	-0.980674717	3.227397499	0.231219647	3.622131817	-0.575706122	-0.119036271	1.226390173	viral
GSM1562127	-0.255344664	-0.68002412	-2.292624893	0.249126789	-2.059113033	-0.344410059	1.45523207	-1.192551325	non-infectious illness
GSM1562128	-0.244975363	-2.043841335	-2.747966709	-2.652600887	-2.113684185	-0.4522773	0.623549864	1.141133596	bacterial
GSM1562129	-0.095637118	0.349073437	1.416219292	-0.519874218	2.276033871	0.002646806	0.092272208	1.084372808	viral
GSM1562130	-0.091182505	1.024351147	1.590388832	0.134105084	1.423382274	-0.692109702	0.743920729	1.613734533	viral
GSM1562131	0.1007702	0.059925106	2.870504913	0.683191654	3.250223629	-0.394373534	0.491994307	0.397451689	viral
GSM1562132	-0.156651293	1.741758374	-0.55515522	0.979185459	-2.148888119	-0.943761465	1.510594771	1.566176702	bacterial
GSM1562133	0.032513822	0.139323262	-0.351745071	0.292183277	-0.858390339	0.133663538	0.495747969	-0.786557863	non-infectious illness
GSM1562134	-0.060256754	-1.449184868	-0.696263733	0.485365269	-1.724699505	-0.414124606	0.42357199	0.806695272	bacterial
GSM1562135	0.24160024	0.007482556	3.558841286	0.749485371	3.711777202	0.014410856	0.003374503	1.14642172	viral
GSM1562136	0.48557588	0.019156483	2.335845528	0.598403332	2.182032101	-0.432603476	0.077277587	0.978658964	viral
GSM1562137	0.361264432	0.975716618	0.388175545	0.769613621	-0.684286602	-0.23704922	0.152071402	0.634670545	bacterial
GSM1562138	-0.279717242	1.096894291	-0.928498688	0.333408711	-1.41553355	-0.668914912	0.994185851	1.725301077	bacterial
GSM1562139	0.431889738	-2.379863235	-1.36666626	-0.210909488	-1.013714321	0.468544366	-0.156039789	-1.329241465	non-infectious illness

Table S6 | Identities of probes used in analysis of GEO datasets

Dataset	ADGRE5 probe	IL16 probe	ISG15 probe	OASL probe
GSE2729	35625_at	1056_s_at	1107_s_at	34491_at
GSE5790	202910_s_at	209827_s_at	205483_s_at	210797_s_at
GSE5808	202910_s_at	209827_s_at	205483_s_at	205660_at
GSE6269	202910_s_at	209827_s_at	205483_s_at	210797_s_at
GSE16129_GPL96	202910_s_at	209828_s_at	205483_s_at	205660_at
GSE17755	AGhsA140621	AGhsB030606	AGhsA060914	AGhsA091609
GSE18090	202910_s_at	209827_s_at	205483_s_at	205660_at
GSE19301	202910_s_at	209827_s_at	205483_s_at	210797_s_at
GSE25504	ILMN_1673363	ILMN_1813572	ILMN_2054019	ILMN_1674811
GSE30119	ILMN_1673363	ILMN_1813572	ILMN_2054019	ILMN_1674811
GSE30550	976_at	3603_at	9636_at	8638_at
GSE33341	202910_s_at	209827_s_at	205483_s_at	205660_at
GSE34205	202910_s_at	1555016_at	205483_s_at	205660_at
GSE35846	ILMN_1676718	ILMN_1813572	ILMN_2054019	ILMN_1674811
GSE36809	202910_s_at	209827_s_at	205483_s_at	210797_s_at
GSE38485	ILMN_1673363	ILMN_1813572	ILMN_2054019	ILMN_1674811
GSE40012	ILMN_1673363	ILMN_1813572	ILMN_2054019	ILMN_1674811
GSE40224	202910_s_at	209827_s_at	205483_s_at	210797_s_at
GSE40366	HqZ_1TXof9d07f55IE	0lQqAgCgp33VSL1U5I	uF4JJC4mp6Ku5KGF70	ukoevq3qjc6TUIS4R0
GSE40396	ILMN_1673363	ILMN_1813572	ILMN_2054019	ILMN_1674811
GSE41752	38124	28110	44378	36193
GSE42834	ILMN_2413508	ILMN_2290628	ILMN_2054019	ILMN_1681721
GSE46743	ILMN_1673363	ILMN_1813572	ILMN_2054019	ILMN_1674811
GSE51808	202910_PM_s_at	209827_PM_s_at	205483_PM_s_at	205660_PM_at
GSE52428	202910_s_at	209827_s_at	205483_s_at	205660_at

Dataset	ADGRE5 probe	IL16 probe	ISG15 probe	OASL probe
GSE61672	ILMN_1673363	ILMN_1813572	ILMN_2054019	ILMN_1674811
GSE4128	M300000343	M200002878	M300017406	M200016125 (OASL1)
GSE13699_GPL6883	ILMN_2413508	ILMN_2290628	ILMN_2054019	ILMN_1681721
GSE13699_GPL6104	ILMN_1676718	ILMN_1813572	ILMN_1813289	ILMN_1681721
GSE14790	No probes available	Ssc.18652.1.S1_at	Ssc.11557.1.A1_at	No probes available
GSE22160	202910_s_at	1555016_at	205483_s_at	205660_at
GSE29429_GPL6947	ILMN_1676718	ILMN_2290628	ILMN_2054019	ILMN_1681721
GSE29429_GPL10558	ILMN_1676718	ILMN_2290628	ILMN_2054019	ILMN_1681721
E-GEOD-50628	202910_s_at	1555016_at	205483_s_at	210797_s_at
GSE57384	10580033	10565218	10451287	10524631 (OASL1)
GSE58287	A_23_P502312	A_24_P73599	A_23_P819	A_23_P139786
GSE67059_GPL6947	ILMN_1676718	ILMN_2290628	ILMN_2054019	ILMN_1681721
GSE67059_GPL10558	ILMN_1676718	ILMN_2290628	ILMN_2054019	ILMN_1681721
GSE68112	ENSRNOG00000004489	ENSRNOG00000011680	ENSRNOG00000021802	ENSRNOG00000001187
GSE69606	202910_s_at	1555016_at	205483_s_at	205660_at

Animation S1 | Resolution of patients with acute respiratory illness (ARI) into three clusters corresponding to bacterial infection, viral infection, and non-infectious illness (GSE63990).

A cohort (GSE63990) having multiple types of pathogen infections was analyzed using a Random Forest - multidimensional scaling (RF-MDS) process that combined the expression levels of ISG15, ISL16, OASL, and ADGRE5 (the four genes of the pan-viral signature), and the expression levels of CEACAM4, LAMP1, PLAC8, and PLA2G7 (the four genes from the *SeptiCyte*^{TM} *LAB* signature; McHugh et al., 2015; reference #41 in manuscript). Animation S1 consists of 181 frames of a GIF (graphics interchange format) file, wherein each frame presents a three-dimensional projection of points from an 8-dimensional space defined by the expression levels of the 8 individual genes comprising the two signatures. The frames have been arranged in a coherent, continuous sequential order. Animation S1 can be run by opening its icon in a web browser such as Google Chrome. When run, Animation S1 provides a 360 degree rotational view of the three-dimensional projection, about one axis. The frames and rotational orientation have been selected to present an obvious visual separation between the three clinical groups (bacterial, viral, and non-infectious illness). Two of the frames from the GIF file are also shown in Figure 8, Panels A and B, of the manuscript. Legend: green points = subjects with bacterial infection; violet points = subjects with viral infection; orange points = subjects with non-infectious illness.

Reference

McHugh, L. et al. A molecular host response assay to discriminate between sepsis and infection-negative systemic inflammation in critically ill patients: discovery and validation in independent cohorts. PLOS Med. 12, e1001916–35 (2015).
