# Supplementary figures and images for: A Four-Biomarker Blood Signature Discriminates Systemic Inflammation Due to Viral Infection Versus Other Etiologies
Source: Sci Rep. 2017 Jun 6;7:2914. doi: 10.1038/s41598-017-02325-8 (PMC5460227; doi:10.1038/s41598-017-02325-8)

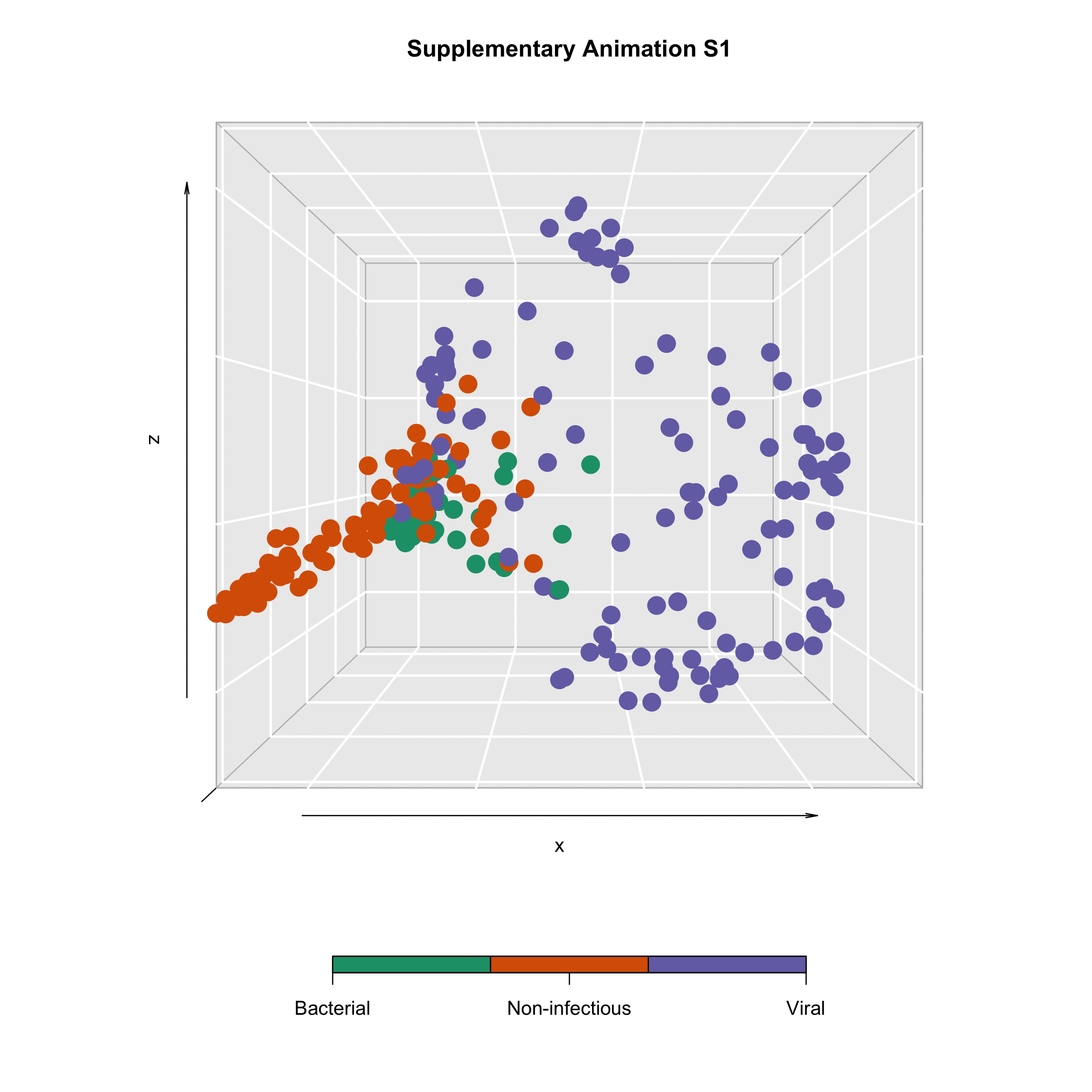

Supplement: Supplementary file 2 — Supplementary Animation S1 [file 41598_2017_2325_MOESM2_ESM.gif]
